# Homeostasis of a representational map in the neocortex

**DOI:** 10.1038/s41593-025-01982-7

**Published:** 2025-06-05

**Authors:** Takahiro Noda, Eike Kienle, Jens-Bastian Eppler, Dominik F. Aschauer, Matthias Kaschube, Yonatan Loewenstein, Simon Rumpel

**Affiliations:** 1 Institute of Physiology, Focus Program Translational Neurosciences, University Medical Center, Johannes Gutenberg University-Mainz, Mainz, Germany; 2 Frankfurt Institute for Advanced Studies (FIAS), Frankfurt, Germany; 3 Department of Comuputer Science and Mathematics, Goethe University Frankfurt, Frankfurt, Germany; 4 The Edmond & Lily Safra Center for Brain Sciences, Department of Cognitive and Brain Sciences, The Alexander Silberman Institute of Life Sciences and The Federmann Center for the Study of Rationality, The Hebrew University of Jerusalem, Jerusalem, Israel

**Keywords:** Neural circuits, Sensory processing

## Abstract

Cortical function, including sensory processing, is surprisingly resilient to neuron loss during aging and neurodegeneration. In this Article, we used the mouse auditory cortex to investigate how homeostatic mechanisms protect the representational map of sounds after neuron loss. We combined two-photon calcium imaging with targeted microablation of 30–40 sound-responsive neurons in layer 2/3. Microablation led to a temporary disturbance of the representational map, but it recovered in the following days. Recovery was primarily driven by neurons that were initially unresponsive to sounds but gained responsiveness and strengthened the network’s correlation structure. By contrast, selective microablation of inhibitory neurons caused prolonged disturbance, characterized by destabilized sound responses. Our results link individual neuron tuning and plasticity to the stability of the population-level representational map, highlighting homeostatic mechanisms that safeguard sensory processing in the neocortex.

## Abstract (web summary)

This study investigates how homeostatic mechanisms endow sensory representations in the auditory cortex with resilience against neuron loss. The map of sounds has the ability to recover after microablation by recruiting previously unresponsive neurons.

## Main

Sensory information is represented in the cortex in the form of a representational map, where the relational properties of sensory stimuli are encoded in the distances between activity patterns^1^. The representational map provides a general framework for describing neuronal population activity along sensory–motor transformations^2^. In sensory areas, the map’s structure primarily encodes perceptual relatedness of stimuli. Experimentally, the map structure can be measured by comparing population responses to different stimuli and has been used to assess neuronal function in various sensory domains^3,4^, including auditory perception^5,6^.

After its formation during development, the stability of a representational map is continuously challenged during adulthood. At the level of sensory-evoked activity, a continuous reformatting of tuning properties of individual neurons has been described as representational drift^7–9^. Moreover, a continuous drop in and drop out of individual neurons from the population response has been demonstrated in various brain regions^4,6,7,10–12^. A possible mechanism for these functional changes in tuning may lie in the substantial plastic changes in the synaptic structure of neocortical circuits that can be observed under environmentally and behaviorally stable conditions^13–15^.

Beyond changes in synaptic connections, which represent network edges, the loss of neurons (the network’s nodes) also challenges the stability of representational maps. Notably, continuous neuronal loss occurs with aging^16^, and this process accelerates in neurodegenerative diseases. Surprisingly, however, cognitive functions remain remarkably robust during healthy aging and even in advanced prodromal neurodegenerative stages, until a tipping point is reached^17,18^.

What mechanisms enable cortical networks to remain robust despite these challenges? One possibility is that specific activity patterns rely on a connectivity architecture, allowing them to tolerate substantial network changes without disrupting activity patterns^19,20^. However, targeted manipulations of a small number of neurons have been shown to notably impact activity^21,22^ and behavior^23,24^. This suggests that neuronal activity patterns may not be inherently robust but are instead stabilized by dynamic feedback mechanisms.

In the body, key physiological parameters like blood pressure and body temperature face various short- and long-term disturbances but are maintained within a functional range through homeostatic regulation^25^. Similarly, homeostasis has been observed in neural systems^26^, including firing rate homeostasis in individual neurons^27^ and homeostasis of neuronal network^28,29^ or pacemaker patterns in the crayfish gastropyloric ganglion^30^. However, the extent to which homeostatic mechanisms influence and stabilize population dynamics in cortical circuits remains less well understood^28^.

Here, we investigated the robustness of a representational map following the experimental removal of functionally selected neurons. We found that although the map was initially impaired, it recovered within a few days. Examining single-neuron tuning changes, we found evidence that cortical circuits actively engage various mechanisms to facilitate homeostatic compensation for this loss.

## Results

### Neurons with volatile tuning form a stable representational map

**Fig. 1:**
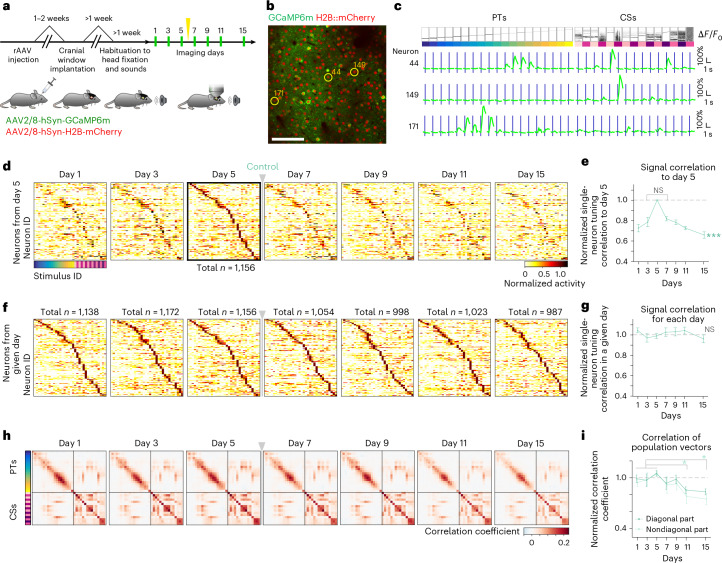
Neurons with unstable tuning properties form a stable representational map. **a**, Schematic of the longitudinal Ca²⁺ imaging experiment in the mouse auditory cortex. On day 6, sham ablation was performed in the control cohort as a reference for other experimental cohorts described later (yellow arrow; see Methods); rAAV, recombinant AAV. **b**, Example imaging plane showing broad GCaMP6m and H2B::mCherry expression in layer 2/3 neurons; scale bar, 100 μm. **c**, Exemplary responses to auditory stimuli (19 PTs, 2–45 kHz; 15 CSs) from the neurons indicated in **b**. *F*, fluorescence. **d**, Normalized response profiles across imaging days for neurons categorized as sound responsive and sorted by stimulus, with the highest response amplitude on day 5. The gray arrow/bar marks the sham procedure in this control cohort. Response profiles of the same 1,156 neurons are shown in all panels (every fourth cell for illustrative purpose); NS, not significant. **e**, Normalized correlation of single-neuron stimulus tuning between day 5 and the other days (data are shown as mean ± s.e.m. across nine mice). Data were analyzed by one-way ANOVA (***P < 0.001) and two-sided paired *t*-test (day 3 versus day 7, day 1 versus day 9; *P* > 0.12). **f**, Same as **d**, but neurons were categorized as sound responsive and sorted by the highest response amplitude on each day (*n* indicates the number of significantly sound-responsive neurons on a given day). **g**, Same as **e** but baseline-normalized single-neuron correlation per day (baseline: average of days 1–5). Data were analyzed by one-way ANOVA; *P* = 0.77. **h**, Similarity matrix of population response vectors across stimuli, averaged across all FOVs per day (*n* = 59 FOVs). **i**, Normalized correlations from **h** averaged across diagonal (dark green) and all nondiagonal (light green) elements relative to baseline (data are shown as mean ± s.e.m. across nine mice). Data were analyzed by two-sided *t*-test with a false discovery rate (FDR) correction (*P < 0.05). See Supplementary Table 1 for details. Source data

**Extended Data Fig. 1:**
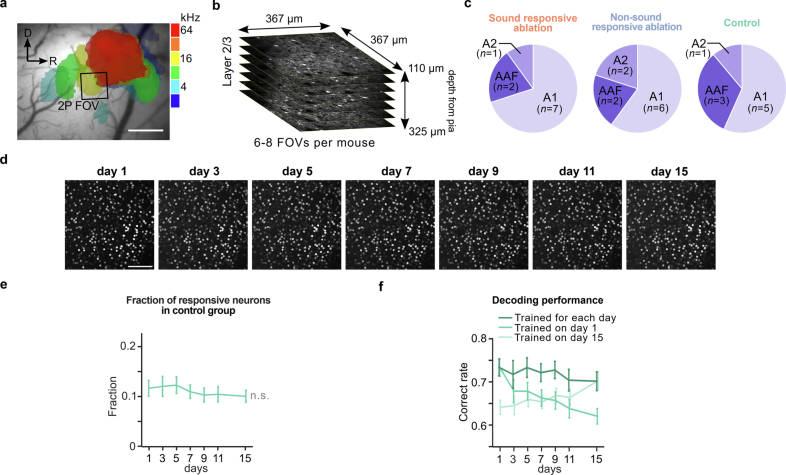
Longitudinal imaging of sound-evoked activity in the mouse auditory cortex. **a**. Example image of intrinsic signals on top of brain surface during burst of pure-tone (2–64 kHz) presentation. Intrinsic signals were recorded to functionally identify auditory fields, within which regions for two-photon calcium imaging were subsequently set. In this example, a region for two-photon imaging was selected as a black square. Scale bar: 500 μm. Intrinsic imaging was repeated 35 times, which corresponds to the total number of mice used for all the 4 experimental cohorts. **b**. Schematics of sequential imaging across layer 2/3 column in the auditory cortex. 6–8 FOVs were imaged per mouse with average z distance of 24.2 μm (± 5.04 standard deviation). **c**. Pie charts show the number of two-photon imaging FOVs in each auditory region (A1: primary auditory cortex; AAF: anterior auditory field; A2: secondary auditory cortex) across mice in each experimental cohort, respectively. Note that although our dataset contains recordings from all of the three major areas, there is a clear bias to A1 in all the main experimental cohorts. The fraction of the imaged auditory regions was comparable across the cohorts. **d**. In vivo two-photon images of H2B::mCherry signal of an example FOV on all the seven imaging days. The distinct labeling in the red channel allows high-fidelity tracking of individual neurons using a signal that is independent of neuronal activity. Scale bar: 100 µm. **e**. Fraction of responsive neurons across days in control cohort without filtering high and low category neurons (*n* = 9, mean ± s.e.m. across mice). **f**. Linear pairwise discriminability calculated by support vector machine averaged across all possible sound pairs and FOVs (*n* = 9, mean ± s.e.m.) plotted across days. The classifier was trained with data from either given (dark green), first (green), or last (light green) imaging day.

To track the long-term dynamics of sound representations in the mouse auditory cortex, we co-injected two adeno-associated virus (AAV) vectors to express GCaMP6m as a neuronal activity marker and H2B::mCherry to label nuclei for precise image registration^6^ (Fig. 1a,b and Methods). Intrinsic signal imaging identified the tonotopic structure for guiding two-photon imaging (Extended Data Fig. 1a). For calcium imaging experiments in awake, head-fixed, passively listening mice, we used a stimulus set of brief (50- to 70-ms) sounds^6^ consisting of 19 sinusoidal pure tones (PTs) and 15 complex sounds (CSs) characterized by temporally modulated power in multiple frequency bands delivered free field using a calibrated speaker at a 70-dB sound pressure level (34 stimuli in total, 10 repetitions each, presented in a pseudorandom order; Fig. 1c). Mice were habituated to head fixation and pre-exposed to stimuli for at least 1 week (refs. ^6,31^). In a given mouse, we imaged neuronal activity in six to eight vertically aligned fields of view (FOVs) within layer 2/3, mainly in the primary auditory cortex (Extended Data Fig. 1b,c). Among the ~140–180 neurons per FOV, a small fraction (~10–14%) showed diverse tuning, whereas most remained unresponsive (Fig. 1c). Neuronal populations were tracked across six time points at 2-day intervals and a final session after 4 days (9,451 neurons, 59 FOVs, 9 mice; Extended Data Fig. 1d).

Consistent with previous reports^6,32^, we observed substantial changes in neuronal responsiveness and tuning over time. Although tuning curves of neurons responsive to any of the 34 stimuli on day 5 tiled the full stimulus set, their responses at earlier and later time points showed a progressively increasing redistribution (Fig. 1d). To quantify tuning changes, we calculated Pearson correlations between tuning curves obtained from individual neurons at different time points by splitting the trials into halves and averaging the responses. For day 5, all trials were used, whereas for other intervals, one half came from day 5 and the other half came from the different imaging days. Tuning correlations showed a gradual, symmetric decline with increasing time intervals (Fig. 1e; data were analyzed by one-way analysis of variance (ANOVA) across days).

Despite tuning volatility in individual neurons, population-level stability was maintained, consistent with previous reports^4,6,10,11^. The number of significantly responsive neurons remained comparable across days, with sorted response profiles showing high similarity (Fig. 1f and Extended Data Fig. 1e). Pearson correlations of tuning curves constructed from split halves of trials within each day were stable, indicating consistent response reliability across trials (Fig. 1g). Similarly, decoding accuracy for pairs of sound stimuli remained stable across imaging sessions when trained and tested on single-trial activity patterns obtained on the same day. However, decoder performance gradually declined when tested using activity patterns recorded at increasing intervals from the training day (Extended Data Fig. 1f).

**Extended Data Fig. 2:**
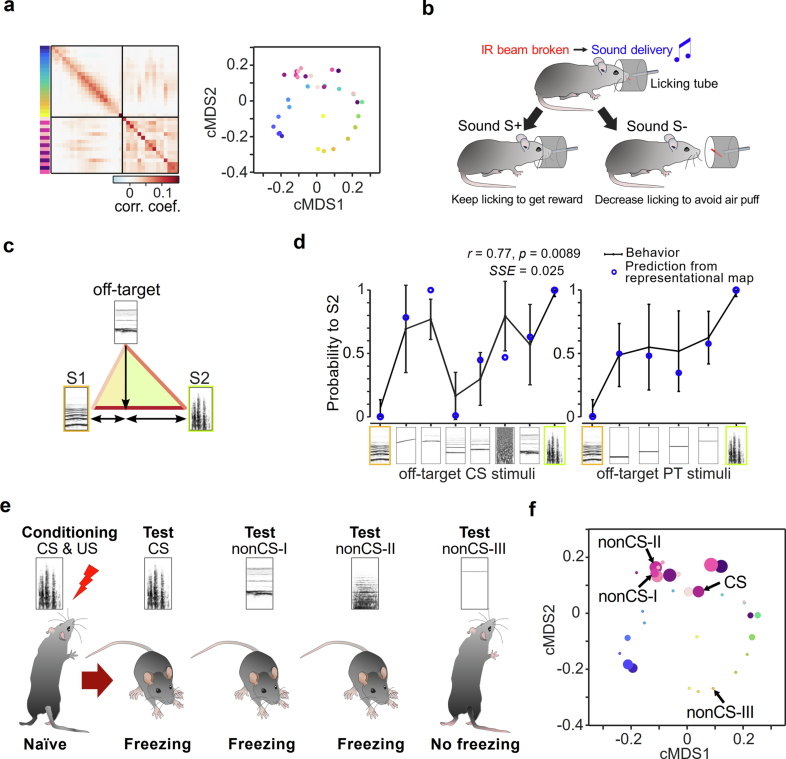
The structure of the representational map in the auditory cortex predicts stimulus generalization in a go/no-go task as well as in classical conditioning. **a**. Grand averaged representational similarity matrix and corresponding dimension-reduced classical Multidimensional scaling (cMDS) display constructed from the dataset acquired in the current study during baseline (n = 29 mice, 3 imaging days). **b**. Schematic of a go/no-go sound discrimination paradigm from a previous study^12^, in which the same set of sound stimuli was used as in the current study. In well-trained mice, licking behavior was measured in response to reinforced S+ and S- stimuli. In addition, spontaneous behavioral categorization of non-reinforced off-target stimuli was used as an estimate of perceptual similarity of a given off-target in relation to the pair of target stimuli. **c**. Schematic of the application of the neurometric similarity matrix to estimate perceptual distances. The length of each side in the triangle of off-target, and reinforced stimuli S1 and S2 is determined by the correlation coefficients. The estimated relative representational similarity of the off-target stimulus to S1 or S2 is defined as the internal dividing point in the line connecting S1 and S2, orthogonally mapped from the off-target point. **d**. Solid black line: Behaviorally evaluated perceptual similarity as the go probability of off-target sounds in go no-go behavioral task (mean ± s.d. across mice, *n* = 12; the data replotted from Bathellier et al., 2012). Blue dots: Prediction of perceptual similarity from the representational similarity matrix in **a** with the metric in **b**. *r* and *p* indicates Pearson correlation and its *p*-value between the behavioral go probability and the prediction from the similarity matrix. *SSE* indicates Sum of squared errors of the similarity matrix-based prediction from the behavioral go probability. **e**. Stimulus generalization to conditioned and non-conditioned sound stimuli from another previously published study^13^, in which the same set of 34 sound stimuli as in the current study was used. One of the stimuli was used as a conditioned stimulus (CS) in a classical auditory-cued fear conditioning paradigm and was paired with a foot shock (US). Four days after conditioning, a generalization test was performed by presenting the CS and the three nonCS sounds (I, II, and III), without foot shock. The conditioned mice exhibited freezing behavior to CS, nonCS-I nonCS-II, but not to nonCS-III. **f**. Dimension-reduced display of the representational map obtained in the current study shown in **a**. Dot diameters depict a measure of neuronal plasticity (increased likelihood of a given sound stimulus to activate the same neuron assembly as the CS) obtained in the previous study^13^. Note, that the proximity between nonCS stimuli and the CS in the representational map allows us to predict the degree of plasticity in sound responses in the auditory cortex as well as the level of behaviorally measured stimulus generalization.

We next assessed sound representation at the population level using a method analogous to representational similarity analysis^1^. In each FOV, we created single-trial population response vectors by averaging calcium signal amplitudes within a 400-ms window after sound presentation. A similarity matrix was constructed by calculating the average Pearson correlation of all pairwise combinations of response vectors, with diagonal elements representing same-stimulus pairs and nondiagonal elements representing different-stimulus pairs^5,6^. To obtain a global measure, we averaged similarity matrices across FOVs for each day (Fig. 1h). This grand-averaged matrix reflects perceptual similarity in mice, predicting generalization in behavioral tasks, and serves as an estimate of the internal representational map of sound relationships^5,6^ (Extended Data Fig. 2). Therefore, the similarity matrix provides a useful estimate of the internal representational map reflecting behaviorally relevant relational information of sounds. Throughout the experiment, similarity matrices remained highly consistent (Fig. 1h). The average correlation of diagonal and nondiagonal elements stayed comparable to the first 5 days, with only a slight gradual decline (Fig. 1i).

Together, these observations showed that the global structure of the representational map is stably maintained despite a substantial drift in single-neuron tuning and a progressive decorrelation of population response vectors for a given stimulus over days, suggesting that plasticity mechanisms constantly preserve higher-order statistics of the population responses.

### Neuron loss temporarily disrupts the representational map

To investigate network mechanisms supporting the dynamic maintenance of the representational map, we examined the effects of permanently removing individual neurons. We considered three possible outcomes: (1) a lasting impairment of the map with no compensation, (2) gradual mitigation driven by baseline tuning volatility (passive compensation) or (3) recovery through specific homeostatic processes (active homeostasis).

**Fig. 2:**
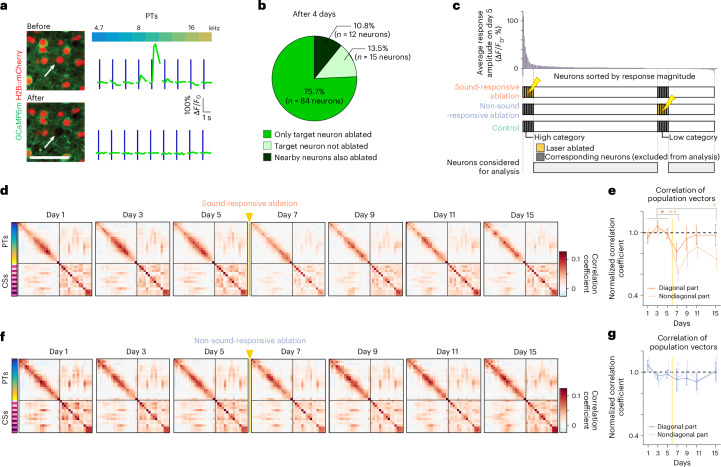
Microablation-induced disturbance and recovery of the representational map. **a**, Two-photon microablation of a functionally characterized neuron with PT tuning. Top: before ablation. Bottom: 2 days after ablation. Microablated neurons showed permanent loss of nuclear and sound-evoked signals; scale bar, 50 μm. White arrow indicates neuron targeted for ablation. **b**, Specificity of the single-neuron ablation protocol obtained in a pilot experiment (two mice). **c**, Target neurons for microablation. Top: sorted sound-evoked amplitudes in a FOV before ablation. In the sound-responsive ablation cohort, four to eight highly responsive neurons per FOV (six to eight vertically aligned FOVs in layer 2/3 per mouse) were targeted (see Methods). In the nonresponsive microablation cohort, a similar number of nonresponsive neurons were targeted. The control cohort had no ablation on day 6. Bottom: targeted or corresponding responsive (high-category) and nonresponsive (low-category) neurons were excluded from the analysis, which focused on the remaining spared neurons (gray bars). **d**, Similarity matrix of population response vectors for all stimuli, averaged across FOVs per day in the sound-responsive ablation cohort (*n* = 69 FOVs; see also Fig. 1h). Yellow arrowhead and vertical line indicate ablation procedure on day 6. **e**, Baseline-normalized correlations averaged across diagonal (dark orange) and nondiagonal (light orange) elements in **d** (data are shown as mean ± s.e.m. across 10 mice). Yellow vertical line indicates ablation procedure on day 6. Data were analyzed by two-sided *t*-test between baseline and postablation days with an FDR correction (*P < 0.05, **P < 0.001). **f**, Same as **d** for the non-sound-responsive ablation cohort (*n* = 73 FOVs). **g**, Same as **e** but baseline-normalized correlations across diagonal (dark blue) and nondiagonal (light blue) elements in the similarity matrices in **f** (*n* = 10 mice). Data were analyzed by two-sided *t*-test for all postablation days; *P* > 0.72. See statistical details in Supplementary Table 1. Source data

**Extended Data Fig. 3:**
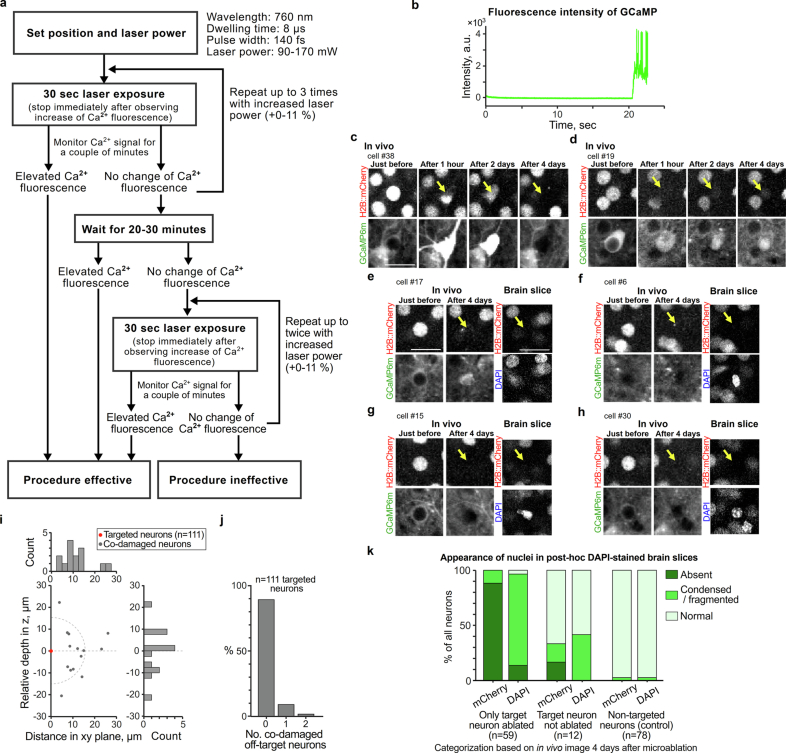
Protocol and validation of the microablation procedure. **a**. Protocol for microablation. See also Methods for further details. **b**. Example trace of Calcium fluorescence during laser exposure on a target neuron when doing point scanning. Ca^2+^ fluorescence was monitored while exposing the laser, and the time-lapse imaging continued for a couple of seconds to a few tens of seconds until we observed abrupt elevation of the signal. In this example, an abrupt increase of fluorescence intensity was observed around 21 sec from the onset of laser exposure. **c**-**d**. Examples of targeted neurons imaged in vivo over the time course of up to 4 days. In **c**, the targeted neuron showed a permanently elevated fluorescence signal one hour after the microablation procedure. After 4 days, both the nucleus fluorescence and the calcium fluorescence were undetectable. In another example targeted neuron in **d**, after 4 days of the microablation procedure, filled calcium fluorescence remained while the nucleus fluorescence was almost eliminated. When target neurons are successfully microablated, in most cases both signals disappeared (**c**, 89.4 %), in rare cases nucleus signal was lost with filled Ca^2+^ fluorescence (**d**, 10.6 %). Scale bar: 20 μm. **e-h**. Evaluation of the effectiveness of the procedure by re-identifying individual targeted neurons in fixed brain sections counter stained by 4’, 6-diamidino-2-phenylindole (DAPI). Four days after the microablation procedure, neurons which lost the H2B::mCherry signal exhibited an absent DAPI signal (**e**) or an abnormally fragmented DAPI morphology (**f**-**h**). Scale bar: 20 μm. **i**. Distribution of collaterally damaged off-target neurons surrounding targeted neurons along xy-axis and z-axis. The red dot indicates the aligned positions of the targeted neurons (*n* = 111). The gray dots are off-target damaged neurons considering a cubic volume of 60 × 60 × 60 μm surrounding the targeted neuron. The majority of neurons suffering collateral damage fall within a 15 μm radius (dashed line) from the targeted neurons (64.3%), which corresponds to the radius that was applied by default to filter out neurons for further image analysis (see Methods). No statistical difference was observed in distributions between xy-distance and absolute z-difference (two-sided Kolmogorov-Smirnov test, *p* = 0.27). **j**. Summary bar plots of the number of co-damaged off-targeted neurons out of 111 ablation experiments targeting a single neuron. **k**. Summary bar plots of nucleus fluorescence signals and post-hoc DAPI signals in targeted neurons categorized based on in vivo images 4 days after the microablation procedure.

Toward this end, we used targeted laser microablation^21,33^ (Fig. 2a and Extended Data Fig. 3a,b), yielding a highly specific and permanent removal of individual, functionally characterized neurons (Fig. 2b, Extended Data Fig. 3c–k and Methods).

**Extended Data Fig. 4:**
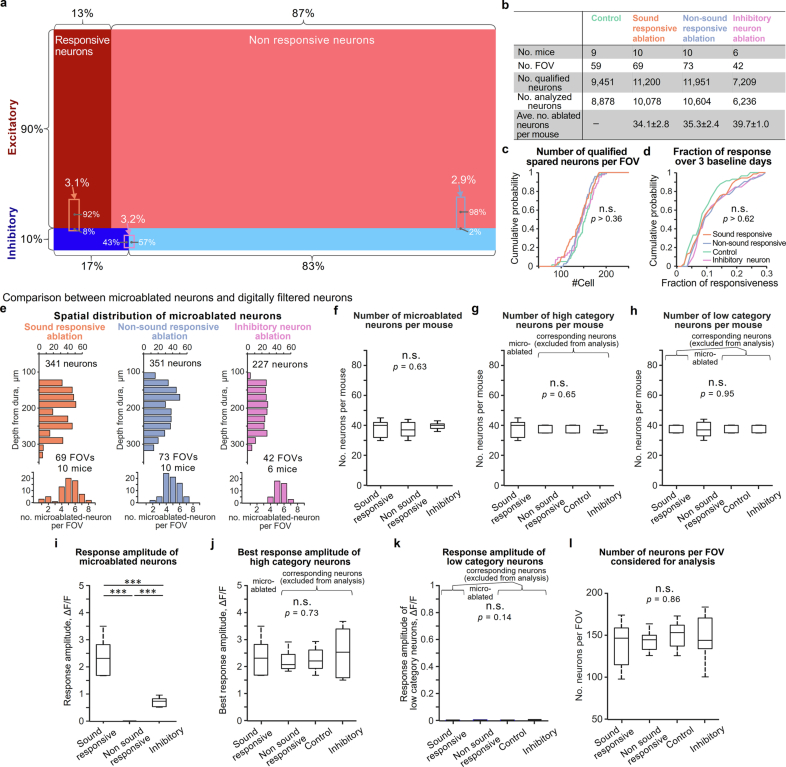
Response statistics of the microablated neurons. **a**. Summary schematic indicating the average fraction of neurons with their categorization of responsiveness and different cell types. For a subset of mice in each experimental cohort (5/10, 5/10, 7/9 mice in sound responsive cohort, non-sound responsive cohort, control cohort, respectively), we also labeled inhibitory cortical neurons by co-injecting an AAV vector encoding the mDlx enhancer system (AAV-mDlx-NLS-tagBFP) with the AAV vectors expressing GCaMP6m and H2B::mCherry (see also Fig. 5 and Extended Data Fig. 8). Therefore, we were able to identify the excitatory and inhibitory types of microablated and spared neurons. Small rectangles indicate the fraction of microablated neurons for each experimental cohort. The length of horizontal side refers to the fraction of microablated neurons out of all qualified neurons per FOV. The fraction along the vertical side refers to the ratio of excitatory and inhibitory neurons in the microablated neurons. The categorization of responsiveness is also given for an additional experimental cohort in which inhibitory neurons were microablated (see also Fig. 6). Orange: sound responsive ablation; Blue: non-sound responsive ablation; Pink: inhibitory neuron ablation. The fraction along the horizontal side in the pink rectangle refers to the ratio of significantly responsive and unresponsive neurons in microablated inhibitory neurons per FOV. **b**. Summary table of number of mice, number of FOV, number of neurons and number of averaged microablated neurons per FOV acquired for each experimental cohort. For sound responsive ablation and non-sound responsive ablation, 41.5 ± 1.75 neurons and 40.3 ± 2.40 neurons were targeted per mouse on average, meaning that 81.9 ± 5.5% and 87.5 ± 2.4% were successfully ablated out of the total number of target neurons respectively, based on the criteria described in Methods. **c**. Cumulative distribution of number of neurons per FOV across all the 7 imaging time points for each experimental cohort. Neurons were included in this analysis after assessing imaging quality (Methods) and removal of high and low category neurons (Fig. 2c, Methods). Two-sided Kolmogorov-Smirnov test between experimental cohorts with FDR correction: *p* > 0.36. **d**. Cumulative distribution of fraction of responsive neurons averaged over the three baseline imaging sessions for each FOV. Two-sided Kolmogorov-Smirnov test for fraction of responsive neurons with FDR correction, all pairwise combination of the experimental cohorts among sound responsive ablation, non-sound responsive ablation, control and inhibitory neuron ablation: *p* > 0.62. **e**. Spatial distribution of microablated neurons for each experimental cohort. Top: distribution along depth from dura. Bottom: Distribution of number of microablated neurons per FOV. **f**. Box plots of number of microablated neurons per mouse across experimental cohorts. One-way ANOVA test, *p* = 0.63. **g**. Number of high category neurons per mouse, which means number of microablated neurons for sound responsive ablation and number of corresponding neurons for the other cohorts. One-way ANOVA test, *p* = 0.65. **h**. Number of low category neurons per mouse, which means number of microablated neurons for non-sound responsive ablation and number of corresponding neurons for the other cohorts. One-way ANOVA test, *p* = 0.95. **i**. Response amplitude of microablated neurons averaged during baseline days (3 and 5). The amplitude of non-sound responsive neurons was defined as the average amplitude across stimuli for each neuron. Two-sided *t*-test with FDR correction between each two of the three experimental cohorts (Sound responsive ablation vs. Non-responsive ablation: *p* = 2.68 × 10^−9^; Sound responsive ablation vs. Inhibitory neuron ablation: 2.99 × 10^−5^; Non-responsive ablation vs. Inhibitory neuron ablation: 3.90 × 10^−9^). *** *p* < 0.001. **j**. Best response amplitude of high category neurons during baseline days. One-way ANOVA test, *p* = 0.73. **k**. Response amplitude of low category neurons during baseline days. The amplitude of non-sound responsive neurons was defined as the average amplitude across stimuli for each neuron. One-way ANOVA test, *p* = 0.14. **l**. Number of spared neurons per FOV considered for analysis. One-way ANOVA test, *p* = 0.86. *n* = 10 mice for sound responsive ablation, *n* = 10 mice for non-sound responsive ablation, *n* = 6 mice for inhibitory neuron ablation for **f**-**l**, *n* = 9 mice for control for **g**,**h**,**j**-**l**. For box plots in **f**-**l**, the middle line, the bottom and top edges of the box indicate median, 25th and 75th percentiles, respectively, and the whiskers are the most extreme data points across mice. See Supplementary Table 2. for detailed statistics.

To select neurons for microablation, we analyzed sound-evoked response amplitudes across neurons in each FOV during baseline (days 3 and 5) before microablation on day 6 (Fig. 2c and Methods). We devised two mouse cohorts, one for targeting sound-responsive neurons (ten mice) and one for targeting nonresponsive neurons (ten mice; Fig. 2c and Extended Data Fig. 4a–d). In each cohort, four to eight neurons per FOV were ablated that were broadly distributed within layer 2/3 (Extended Data Fig. 4e). On average, we ablated 34.1 ± 2.8 neurons per mouse in the sound-responsive cohort and 35.3 ± 2.4 in the non-sound-responsive cohort, ~3% of imaged neurons. This number of individually microablated neurons reflects the maximum achievable in a single session using our procedure.

For the analyses assessing microablation effects on the spared network, we excluded ablated neurons from analysis at all time points. Additionally, we excluded a similar number of neurons with similarly high or low sound-evoked responses to allow comparability across cohorts (‘high-category’ and ‘low-category’ neurons; Fig. 2c). In both experimental cohorts, neuron numbers and response distributions were comparable across FOVs before microablation (Extended Data Fig. 4c,d). We analyzed 10,078 neurons (69 FOVs, ten mice) in the sound-responsive ablation cohort, 10,604 neurons (73 FOVs, ten mice) in the non-sound-responsive ablation cohort and 8,878 neurons (59 FOVs, nine mice) in the control cohort after exclusion of high- and low-category neurons, enabling an unbiased comparison of the effects of microablation on the spared network (Extended Data Fig. 4c–l).

**Extended Data Fig. 5:**
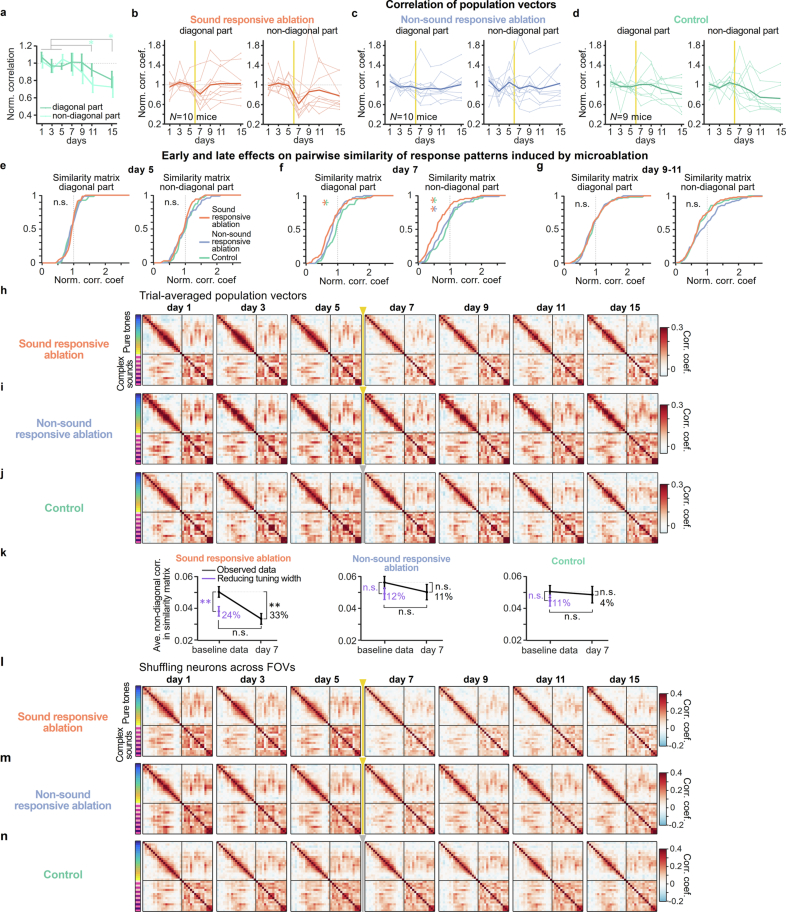
Additional analyses of the effects on the representational maps induced by microablation. **a**. Normalized correlations averaged across diagonal elements (dark green) and non-diagonal elements (light green) in the similarity matrices analogous to Fig. 1i (mean ± s.e.m. across mice, *n* = 9), however, applying the same filter to remove high and low category neurons (Methods) as in the analyses for the other experimental cohorts. Two-sided *t*-test between baseline days vs. days after ablation with FDR correction, in average of diagonal elements (Average of diagonal elements: *p* = 0.93, 0.93, 0.53, 0.084 for day 7, 9, 11, 15). * *p* < 0.01. **b**-**d**. To illustrate observed variability in the data obtained from individual mice, the thick lines show average (as in in Fig. 2e, g) and thin lines show individual normalized correlation of population vectors in diagonal part (left) and in non-diagonal part (right) from individual mice as independent samples, respectively, for sound responsive ablation cohort (**b**), non-sound responsive cohort (**c**) and control cohort (**d**). **e**. Cumulative distribution of baseline normalized correlation coefficient along diagonal elements (left) and along non-diagonal elements (right) on day 5 in the representational map for each experimental cohort (*n* = 69, 73, and 59 FOVs for sound responsive, non-sound responsive and control cohorts, respectively). To make sure that the distributions of normalized correlation in the representational map during baseline were comparable between the experimental cohorts, difference in these cumulative distributions was tested by Dunn’s test: Q_critical_ = 2.39, all Q values between groups for both diagonal and non-diagonal components in the similarity matrices, Q < Q_critical_. Two-sided Mann-Whitney *U* test between groups with FDR correction for both the diagonal and the non-diagonal components, all *p* values, *p* > 0.5. **f**. Cumulative distribution of baseline normalized correlation coefficient on day 7 in the representational map. Difference in these cumulative distributions between the experimental cohorts was again tested by Dunn’s test as well as Two-sided Mann-Whitney *U* test. The cumulative distribution in sound responsive ablation cohort significantly relatively shifted to lower correlation than the other cohorts: For diagonal components, Q_critical_ = 2.39; Sound responsive ablation vs. Control: Q = 2.93; Sound responsive ablation vs. Non-responsive ablation: Q < Q_critical_; Non-responsive ablation vs. Control: Q < Q_critical_; Two-sided Mann-Whitney *U* test with FDR correction between groups (Two-color asterisks, * *p* < 0.05; see Supplementary Table 2.); Sound responsive ablation vs. Non-responsive ablation: *p* = 0.077; Non-responsive ablation vs. Control: *p* = 0.20; For non-diagonal components, Sound responsive ablation vs. Control: Q = 3.44; Sound responsive ablation vs. Non-responsive ablation: Q = 2.57; Non-responsive ablation vs. Control: Q < Q_critical_; Two-sided Mann-Whitney *U* test with FDR correction between groups (* *p* < 0.05). **g**. Cumulative distribution of baseline normalized correlation coefficient during day 9–11 in the map. Dunn’s test: Q_critical_ = 2.39, all Q values between groups for both diagonal and non-diagonal components in the similarity matrices, Q < Q_critical_. Two-sided Mann-Whitney *U* test between groups with FDR correction for both the diagonal and the non-diagonal components, all *p* values, *p* > 0.08. **h**-**j**. Similarity matrices from trial-averaged population response vectors across days in sound responsive ablation condition, non-sound responsive ablation condition and control, respectively. **k**. Simulated non-diagonal correlation of similarity matrix by experimentally derived downscaled tuning curve in individual neurons. Two-sided *t*-test between baseline correlation vs. simulated correlation after downscaling (mean ± s.e.m. across mice; Sound responsive cohort: *p* = 8.3 × 10^−3^; Non-sound responsive cohort: *p* = 0.19; Control: *p* = 0.27). * *p* < 0.05, ** *p* < 0.01; Two-sided *t*-test between simulated correlation after downscaling vs. correlation on day 7 (* *p* < 0.05). *n* = 10 mice for sound responsive ablation, *n* = 10 mice for non-sound responsive ablation, *n* = 9 mice for control. **l-n**. Similarity matrices across days from population response vectors after shuffling neurons across FOVs for each experimental cohort. See Supplementary Table 2. for detailed statistics.

To evaluate the representational map’s sensitivity to neuron loss, we constructed averaged similarity matrices for each time point. During baseline, the map remained stable in both the sound-responsive and non-sound-responsive ablation cohorts, similar to the control cohort (Fig. 2d–g and Extended Data Fig. 5a). Notably, after microablating sound-responsive neurons, the similarity matrix showed reduced contrast, with lower correlation values, but gradually recovered to baseline levels (Fig. 2d). This was reflected in the baseline-normalized average correlation along the diagonal, indicating stable population response reliability (Fig. 2e and Extended Data Fig. 5b). Nondiagonal correlations, representing response similarities between different stimuli, also showed a general recovery, except for day 15 where the correlation did not fully recover but reached a level comparable to that of the control cohort (Fig. 2e and Extended Data Fig. 5b–g).

By contrast, in the cohort of mice in which non-sound-responsive neurons were mircoablated, the structure of the representational map was largely stable throughout the experiment (Fig. 2f,g and Extended Data Fig. 5c–g).

In summary, our analyses revealed that selectively removing 30–40 highly sound-responsive neurons temporarily disrupted the representational map in the spared layer 2/3 neurons. However, the map’s structure recovered to baseline levels within 3 days of microablation.

### Single-neuron response changes underlying map plasticity

**Fig. 3:**
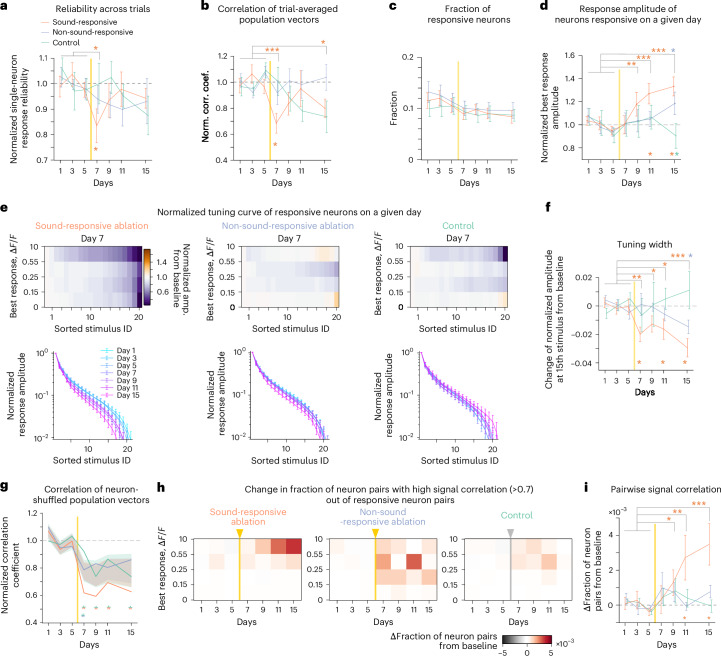
Microablation induces changes in single-neuron response properties at multiple timescales. **a**, Baseline-normalized response reliability across trials of all single neurons, averaged per day (orange, sound-responsive ablation; blue, non-sound-responsive ablation; green, control). Data were analyzed by two-sided *t*-test with an FDR correction (top asterisks; *P < 0.05) and permutation test across groups (bottom asterisk) on day 7 (sound-responsive cohort: *P* = 0.034). **b**, Baseline-normalized correlations averaged across nondiagonal elements in the similarity matrix constructed from trial-averaged population vectors. Data were analyzed by two-sided *t*-test (sound-responsive cohort on day 7: *P* = 7.90 × 10⁻^4^; *P < 0.05 and ***P < 0.001) and permutation test (bottom asterisks; *P < 0.01). **c**, Fraction of sound-responsive neurons across imaging days. Data were analyzed by one-way ANOVA across days (*P* ≥ 0.20 for all the cohorts). **d**, Baseline-normalized best response amplitude per day. Data were analyzed by two-sided *t*-test with an FDR correction (top asterisks; sound-responsive cohort: *P* = 0.98, 4.5 × 10^−3^, 1.8 × 10^−5^ and 3.7 × 10^−7^ for days 7, 9, 11 and 15, respectively; *P < 0.05, **P < 0.01 and ***P < 0.001) and permutation test (bottom asterisks; *P < 0.01). **e**, Normalized tuning curve of responsive neurons. Stimulus responses were sorted, normalized and averaged across neurons within one of four categories according to their best response amplitude. Top: color-coded amplitude changes on day 7 from baseline. Bottom: normalized tuning curves for neurons with the largest best response category over days (left, sound-responsive ablation; middle, non-sound-responsive ablation; right, control). **f**, Change in normalized response amplitude at the 15th largest stimulus index across days. Data were analyzed by two-sided *t*-test with an FDR correction (top; sound-responsive cohort: *P* = 1.9 × 10⁻^3^, 0.049, 0.026 and 5.7 × 10⁻^5^ for days 7, 9, 11 and 15, respectively; *P < 0.05, **P < 0.01 and ***P < 0.001) and permutation test (bottom; *P < 0.05). **g**, Average baseline-normalized nondiagonal correlations from similarity matrices constructed from population vectors shuffled across FOVs. Shaded areas indicate the 95% confidence interval. Data were analyzed by permutation test (*P* < 6.0 × 10⁻^3^ for all postablation days). Comparisons were made between sound-responsive and control and non-sound-responsive and control cohorts (bottom; *P < 0.01). **h**, Change in the fraction of highly correlated (>0.7) neuron pairs after baseline subtraction, categorized by best response amplitudes (averaged over the two neurons; sound-responsive ablation: data were analyzed by two-way ANOVA, *P* = 2.8 × 10⁻^3^ (days), *P* = 2.6 × 10⁻^5^ (amplitude bins); non-sound-responsive ablation and control: *P* > 0.08). **i**, Change in fraction of high-correlation neuron pairs in the highest response category (**h**). Data were analyzed by two-sided *t*-test with an FDR correction (sound-responsive cohort: *P* = 0.29, 0.017, 1.7 × 10⁻^3^ and 1.1 × 10⁻^4^ for days 7, 9, 11 and 15, respectively; *P < 0.05, **P < 0.01 and ***P < 0.001) and permutation test (bottom; *P < 0.05). Note that the maximal change corresponds to a threefold increase to baseline. Data are presented as mean ± s.e.m. across mice (except for **g** and **h**); *n* = 10 (sound-responsive ablation), *n* = 10 (non-sound-responsive ablation) and *n* = 9 (control). See Supplementary Table 1 for detailed statistics. Yellow vertical lines indicate ablation on day 6.

The representational map emerges from the collective statistics of the tuning properties of individual neurons^2,34^. To explore the link between neuronal activity and representational geometry, we analyzed changes in the responses of individual neurons. A transient drop in the diagonal entries of similarity matrices constructed from single-trial population response vectors suggested a reduced reliability of sound-evoked responses after microablation. Measuring response reliability in individual neurons using tuning curve correlations from subsampled trials (see Methods), we observed a temporary decrease in the sound-responsive ablation cohort, whereas stability was maintained in the other cohorts (Fig. 3a).

To isolate the effects of reduced response reliability from other factors contributing to the temporary disturbance in similarity matrices, we first averaged population vectors across trials for each stimulus and then constructed similarity matrices using Pearson correlations (Extended Data Fig. 5h–j). In this case diagonal elements were equal to 1 and remained unaffected by construction. Nondiagonal correlations showed a significant drop on day 7, followed by recovery, although day 15 correlations remained slightly below baseline but matched the control cohort (Fig. 3b and Extended Data Fig. 5h–j). This suggests that, beyond response reliability, changes in neuronal tuning also contribute to representational map remodeling.

To identify what changes in tuning of individual neurons may be involved, we next investigated several major single-neuron response properties. When categorizing neurons in sound-responsive or non-sound-responsive categories, based on a significant response to at least one stimulus (Methods), we did not observe a significant change in the overall fraction of sound-responsive neurons (Fig. 3c).

The average response amplitude elicited by the stimulus giving rise to the maximal response (best response amplitude) increased in the cohort where sound-responsive neurons were microablated (Fig. 3d). Notably, this effect emerged only days later, suggesting that it contributed to the recovery of the representational map rather than its initial disturbance (Fig. 3d).

Given that broader or narrower tuning in individual neurons can increase or decrease the correlation between two population responses to different stimuli, we examined changes in tuning width in responsive neurons. In the sound-responsive ablation cohort, but not the others, tuning width decreased the day after microablation, especially in neurons with large response amplitudes (Fig. 3e,f). Artificially narrowing baseline tuning curves to a similar degree reproduced the reduction in nondiagonal correlations in similarity matrices (Extended Data Fig. 5k). Thus, reduced tuning width, along with decreased response reliability, likely contributed to the transient disturbance of the representational map. However, as tuning remained narrow in later days, additional single-neuron changes may underlie the map’s recovery.

To assess whether changes in second-order response statistics contribute to recovery, we shuffled data from a given day by permuting neurons across FOVs and mice, preserving single-neuron properties while disrupting local tuning correlations. Constructed similarity matrices from shuffled, trial-averaged data showed a general decline in normalized correlations across all cohorts (Fig. 3g). In the sound-responsive ablation cohort, correlations were lower on day 7, aligning with previous findings. However, unlike the original data, no recovery was observed in shuffled data after microablation (Fig. 3g and Extended Data Fig. 5l–n). This suggests that second-order correlations in local response patterns may play a role in representational map recovery.

As a simple readout of local second-order response statistics, we quantified the fraction of responsive neuron pairs in the spared network with high signal correlations (>0.7) per day. In the sound-responsive ablation cohort, this fraction increased over time, particularly among neurons with large best response amplitudes (Fig. 3h, left), while remaining unchanged in the other cohorts (Fig. 3h, right). Compared to the control cohort, the delayed increase was specific to the sound-responsive ablation cohort (Fig. 3i), suggesting that tuning in the spared network became more locally homogeneous.

**Extended Data Fig. 6:**
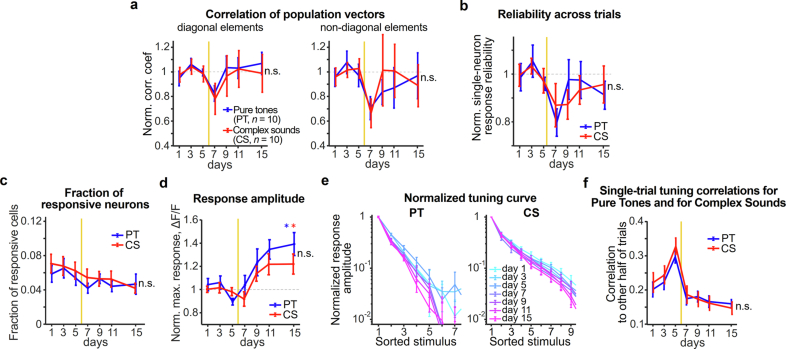
Pure tone and complex sound responses of layer 2/3 neurons are similarly affected by the microablation of sound responsive neurons. To assess how far pure tone and complex sound responses in the spared network may be differentially affected by the removal of sound responsive neurons, we run analogous analyses as shown in the corresponding main figures, however, split the data into the two categories of stimulus responses. It should be noted that this reduced the size of the dataset, introducing increased variability. Nevertheless, we observed largely similar effects for both categories of stimuli. **a**. Analogous to Fig. 2e, baseline-normalized Pearson correlations of population vectors averaged across diagonal elements (left) and non-diagonal elements (right), split into pure tones (blue) and complex sounds (red), for sound responsive ablation cohort. Two-way ANOVA, *p* ≥ 0.18 both between PT and CS stimuli and across days for both diagonal elements and non-diagonal elements;. Two-sided *t*-test between PT and CS stimuli with FDR correction across post-ablation days, *p* > 0.57 for both diagonal and non-diagonal elements. **b**. Analogous to Fig. 3a, normalized response reliability across trials for PT and CS stimuli. Two-way ANOVA, *p* = 0.91 between PT and CS tunings (See Supplementary Table 2.). Two-sided *t*-test of reliability between PT response vs. CS response with FDR correction across post ablation days (day 7, 9, 11, 15): *p* > 0.50. **c**. Analogous to Fig. 3c, fraction of responsiveness for PT and CS stimuli. Two-way ANOVA, *p* = 0.24 between PT and CS stimuli; *p* = 0.092 across days. Two-sided *t*-test of response fraction between PT response vs. CS response with FDR correction across post ablation days: *p* > 0.92. **d**. Analogous to Fig. 3d, maximum response amplitude for PT and CS stimuli in sound responsive neurons. Two-way ANOVA, *p* = 0.054 between PT and CS tunings; *p* = 4.69 × 10^−8^ across days. Two-sided *t*-test of maximum amplitude between PT response vs. CS response with FDR correction across post ablation days: *p* > 0.42. **e**. Analogous to Fig. 3e, normalized tuning curves for pure tones (left) and complex sounds (right) overlaid across days. Two-sided *t*-test with FDR correction between PT tuning vs CS tuning at 15th stimulus index on post-ablation days: *p* > 0.27. **f**. Stability of single-trial responses between PT and CS stimuli analyzed analogous to a previous study^43^. Two-way ANOVA, *p* = 0.45 between PT and CS tunings; *p* = 2.13 × 10^−12^ across days. Two-sided *t*-test of single-trial stability between PT and CS stimuli, on each day: *p* > 0.32. Data presented as mean ± s.e.m. across mice. *n* = 10 mice for sound responsive ablation for both types of sounds (pure tones, blue and complex sounds, red) for all panels.

In summary, our analysis suggests that the transient drop in response reliability and tuning width primarily disrupts the representational map, whereas the delayed increase in local signal correlations helps to restore it. Responses to PTs and CSs in spared layer 2/3 neurons were similarly affected by microablation (Extended Data Fig. 6).

### Neurons gaining sound responsiveness support map recovery

**Fig. 4:**
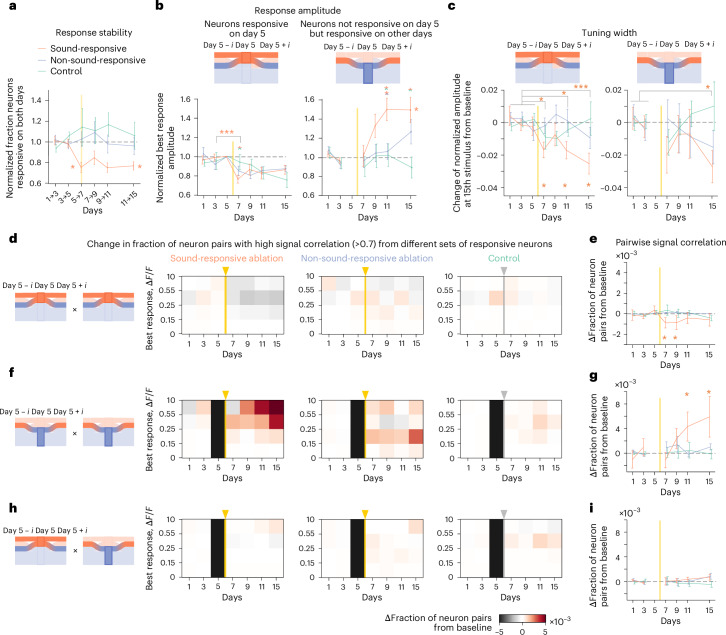
Major changes in single-neuron response properties are primarily mediated by neurons gaining responsiveness after microablation. **a**, Response stability quantified as the fraction of neurons classified as sound responsive on two consecutive imaging days. Data were analyzed by one-way ANOVA across days (right asterisk; *P < 0.01) and two-sided *t*-test between baseline change and change from days 5 to 7 (bottom asterisk; *P < 0.01). **b**, Top: schematic illustrating the categorization of neurons based on responsiveness on day 5 before microablation. One group (left) included neurons responsive on day 5, tracking their responses on all other days regardless of continued responsiveness. The other group (right) included neurons unresponsive on day 5 but classified responsive on other days. Bottom: normalized best response amplitude across cohorts. Left: data were analyzed by *t*-test (day 3 versus day 7, sound-responsive cohort: *P* = 4.54 × 10⁻^4^; ***P < 0.001; group comparison on day 7, one-sided *t*-test, FDR correction, *P < 0.05). Right: data were analyzed by one-way ANOVA across days (right asterisk; *P < 0.01). **c**, Change in tuning width for neurons responsive on day 5 (left) and for neurons unresponsive on day 5 but responsive on another day (right), measured as normalized response amplitudes of the 15th stimulus in the sorted tuning curve (same as Fig. 3f). Data were analyzed by two-sided *t*-test (sound-responsive cohort: *P* = 0.011, 0.25, 0.011 and 1.0 × 10^−^^3^ for days 7, 9, 11 and 15; *P < 0.05 and ***P < 0.001) and permutation test across groups (bottom asterisks; *P < 0.05). **d**, Change in the fraction of neuron pairs with high signal correlation, both responsive on day 5. Color maps show the difference in fraction normalized to baseline for four categories of neuron pairs sorted by their mean best response amplitude (left: sound responsive; middle: non-sound responsive; right: control). For the sound-responsive cohort, data were analyzed by two-way ANOVA (*P* = 9.5 × 10^−5^ across days, *P* = 0.0010 across amplitude bins) and *t*-test (days 9–15, *P* = 8.7 × 10^−6^). For the non-sound-responsive and control cohorts, *P* > 0.37, except the control cohort (*P* = 0.039 across days). **e**, Change in fraction of neuron pairs with high signal correlation in the largest best response category (**d**). Data were analyzed by permutation test (sound-responsive cohort: *P* = 0.010, 0.035, 0.13 and 0.21 for days 7, 9, 11 and 15, respectively); *P < 0.05. **f**,**g**, Same as **d** and **e** but for pairs of neurons categorized as unresponsive on day 5 but responsive on the other day (sound-responsive cohort: two-way ANOVA (**f**; left: *P* = 0.012 across days, *P* = 0.028 across amplitude bins) and *t*-test (days 9–15, *P* = 0.034); non-sound-responsive and control cohorts: (**f**; middle and right) *P* > 0.22, except non-sound-responsive (*P* = 0.044 across amplitude bins); permutation test (**g**; sound-responsive cohort: *P* = 0.91, 0.21, 0.040 and 0.010 for days 7, 9, 11 and 15, respectively); *P < 0.05). **h**,**i**, Same as **d** and **e** but for mixed pairs (one responsive on day 5 and one categorized as unresponsive on day 5 but responsive on the other day). Note the strong increase in pairs with high signal correlation that were categorized as unresponsive on day 5 (sound-responsive cohort: two-way ANOVA (**h**; left: *P* = 0.89 across days, *P* = 0.038 across amplitude bins); non-sound-responsive and control cohorts: (**h**; middle and right) *P* > 0.059; permutation test (**i**; all postablation days: *P* > 0.11 for all cohorts)). Data are presented as mean ± s.e.m. across mice (except for **d**, **f** and **h**); *n* = 10 (sound-responsive ablation), *n* = 10 (non-sound-responsive ablation) and *n* = 9 (control). Yellow vertical lines indicate the time point of ablation procedure. See Supplementary Table 1 for detailed statistics.

The previous analyses focused on single-neuron responses per imaging day, without considering their history of sound-evoked responses in earlier sessions. However, our initial analyses (Fig. 1) showed that, under basal conditions, individual neurons continuously change their responsiveness over days. We next examined how targeted neuron loss affects this drift by quantifying the overlap of neurons showing significant sound responses on two subsequent imaging days. After ablating sound-responsive neurons, the stability of sound responsiveness significantly decreased and remained low throughout the experiment. By contrast, responsiveness in other cohorts remained at baseline levels (Fig. 4a), indicating that microablation induces a longer-lasting increase in the drift rate of sound responsiveness.

We next tested how microablation-induced changes in response properties relate to the history of responsiveness of individual neurons. We considered two categories of neurons: neurons displaying significant sound responses on day 5 before the microablation and neurons with no significant sound responses on day 5 but that were responsive on another imaging day.

**Extended Data Fig. 7:**
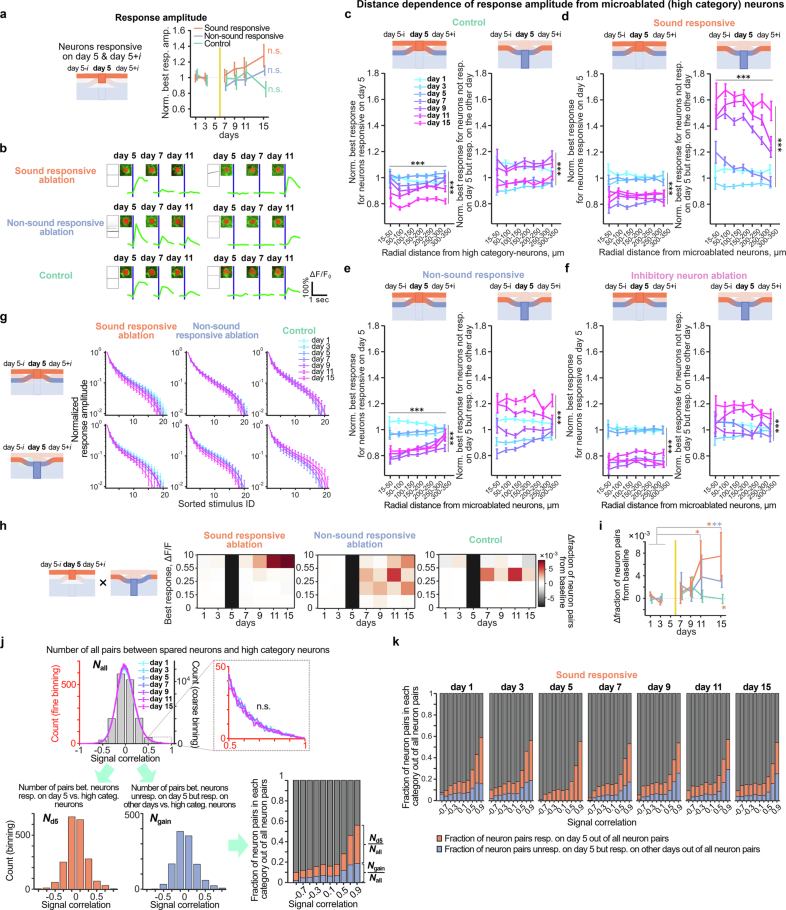
Additional analyses of the microablation-induced effects on single-neuron response dynamics and pairwise signal correlation. **a**. Best response amplitude of neurons responsive on both day 5 and day 5±i normalized by the average best response during early baseline days (1 and 3). One-way ANOVA test (mean ± s.e.m. across mice), *p* > 0.26 for all the three experimental cohorts. **b**. Exemplary sound-evoked Ca^2+^ transients of neurons which responded to sounds on day 5 and reduced the amplitudes in these responses in following days after ablation in sound responsive ablation (top left), non-sound responsive ablation (middle left), and control (bottom left), respectively. Right: Exemplary sound-evoked Ca^2+^ transients of neurons unresponsive on day 5, but becoming responsive in the following days after ablation. **c**. Assessing the three-dimensional spatial extent of the microablation-induced effect on normalized best response amplitudes in the spared neurons. In the control cohort, the high category neurons with strong sound-evoked responses that were excluded from the further analysis were considered as reference (see Methods). Spared neurons were split into neurons responsive on day 5 (Left) and neurons gaining responsiveness (Right). In a given mouse, the three-dimensional distance of a spared neuron to each of the 30–40 microablated neurons (or high category neurons in the control cohort) was calculated, that is contributed multiple distance measurements associated with the same effect on the best response amplitude. All distance combinations were categorized into distance bins ranging between 15 and 350 μm. For each distance bin, the corresponding best response amplitude in each neuron was normalized to the average baseline amplitude for each mouse, the normalized best response was averaged across neurons. Data presented as mean ± s.e.m. across neurons (the detail n numbers of neurons in each split group across days for different experimental cohorts were described in Supplementary Table 3.). In the control cohort, normalized best responses of neurons responsive on day 5 gradually decreased over days with generally constant amplitude across distance, but exhibited modest reduction of normalized amplitude on later (day 11 and 15) days (Two-sided *t*-test between short and long distance, day 1 to day 15: see detail statistics below). Normalized best responses of newly responsive neurons remained rather flat across distance and did not show any systematic change during and after baseline days (Two-sided *t*-test between short and long distance). **d**-**f**. Same as **c**, but from microablated neurons for sound responsive ablation, for non-responsive ablation, and for inhibitory neuron ablation, respectively. In sound responsive ablation, normalized best responses of neurons responsive on day 5 decreased over days, but with constant amplitude across distance (Two-way ANOVA across distance bins or across days; *** *p* < 0.001; see Supplementary Table 2.). On the other hand, normalized best responses of newly responsive neurons strongly increased the amplitudes later days (Two-way ANOVA across distance bins or across days; *** *p* < 0.001). Three days after microablation, the normalized amplitude with nearby distance exhibited larger amplitude than the amplitude distant from ablated neurons (Two-sided *t*-test between short (< 100 μm) and long distance (250–350 μm), *p* = 0.49, 0.42, 1.0 × 10^−4^, 1.0 × 10^−5^, 2.0 × 10^−4^, 0.12 for day 1,3,7,9,11,15). For non-sound responsive ablation, normalized best responses of neurons responsive on day 5 decreased over days and the reduction was more prominent at shorter distance from ablated neurons (Two-way ANOVA across distance bins or across days; *** *p* < 0.001; Two-sided *t*-test between short and long distance, see details below). On the other hand, normalized best responses of newly responsive neurons did not change over distance, but showed slight increase in amplitude later days after ablation (Two-way ANOVA across distance bins or across days; *** *p* < 0.001). The normalized amplitude on day 7 was suppressed at shorter distance, but conversely increased later days (Two-sided *t*-test between short and long distance: see details below). **g**. Same as Fig. 3e bottom, but normalized tuning curves of day 5 responsive neurons (top) and of neurons unresponsive on day 5 but responsive on the other day (bottom), for sound responsive cohort (left), non-sound responsive cohort (middle) and control cohort (right), respectively (mean ± s.e.m. across mice). **h**. Same as Fig. 4c, but the colormaps displaying the change in the fraction of high-signal-correlation neuron pairs, one of which was from neurons responsive on both day 5 and any other day, and the other of which was from neurons unresponsive on day 5 but responsive on any other day. **i**. Change in the fraction of neuron pairs with high signal correlation from baseline at the largest response amplitude bin in the corresponding colormaps in **i**. Two-sided *t*-test between baseline days and days after ablation with FDR correction (mean ± s.e.m. across mice; Sound responsive cohort: *p* = 0.24, 0.067, 0.015, 0.018 for day 7, 9, 11, 15). * *p* < 0.05. Permutation test for group comparison: *p* > 0.05 from day 7–11 for all the three cohorts, but *p* < 0.05 on day 15 for sound responsive cohort. **j**. Schematic of the procedure to analyze the change in pairwise signal correlations shown in Fig. 5. First, the distributions of all pairwise signal correlations between high category neurons and spared neurons are calculated on a given day (*N*_all_). Note, each spared neuron contributes 30–40 pairwise correlations and vast majority of the spared neurons is categorized as non-responsive on a given day. Thus, the distribution is largely centered around 0 and highly stable over days, as can be seen with small bin sizes (lines, red axis on the left; One-way ANOVA test of cumulative distribution of signal correlation > 0.6 over days; *p* = 0.82) and coarser bin sizes used for later analysis steps (grey bars, black axis on the right). The inset shows the tail of high pairwise correlations. Second, from this distribution only the neuron pairs between high category neurons and the neurons responsive on day 5 (*N*_d5_, orange histogram) and the neuron pairs between high category neurons and the neurons not responsive on day 5, but responsive on another day (*N*_gain_, blue histogram) are considered and their fraction in the respective bins is calculated (bar stacks on the bottom right shown for day 3). Note, as expected, the pairwise signal correlations for responsive spared neurons are enriched for high signal correlation values. **k**. Bar stacks of fraction of neuron pairs for all the signal correlation bins over days in sound responsive cohort in Fig. 5a. On day 5, by construction there is no contribution of *N*_gain_ neuron pairs. Note, the fractions of *N*_gain_ (blue) and *N*_d5_ (orange) neuron pairs shift after microablation towards *N*_gain_ neuron pairs. The color maps in Fig. 5 show these changes in fraction over time after normalization to baseline days 1 and 3 by subtraction of the average fraction, as shown in the two leftmost bar stacks. The bars on the left side of the color maps in Fig. 5 show this average baseline value for the respective bins. Data presented as mean ± s.e.m. across mice for **a**, **g**, **h**-**i** (*n* = 10 mice for sound responsive ablation, *n* = 10 mice for non-sound responsive ablation, *n* = 9 mice for control).

We re-examined the increase in best response amplitudes after ablating sound-responsive neurons. In previously responsive neurons of the first category, best response amplitudes on day 7 were significantly lower in the sound-responsive ablation cohort and less reduced in the non-sound-responsive ablation cohort (Fig. 4b). However, in neurons that remained responsive after microablation, amplitudes stayed comparable to baseline (Extended Data Fig. 7a), suggesting that the overall reduction was mainly due to a fraction of neurons losing all responsiveness.

In neurons of the second category that were nonresponsive on day 5 but showed responses on other days before or after microablation^32^ (Extended Data Fig. 7b), best response amplitudes increased substantially but with a delay after ablating sound-responsive neurons (Fig. 4b). This indicated that the overall increase in best response amplitudes (Fig. 3d) is mainly driven by neurons that gained responsiveness after microablation.

We examined the spatial spread of microablation-induced effects on best response amplitude (Extended Data Fig. 7c–f). In the sound-responsive ablation cohort, where changes were observed, these effects were mainly confined to neurons within ~200 μm of the ablated neurons.

Reanalyzing the narrowing of tuning width (Fig. 3f), we found a more pronounced effect in neurons that were responsive before microablation in the sound-responsive ablation cohort (Fig. 4c, left). By contrast, tuning width remained stable in the other cohorts. Neurons that were unresponsive on day 5 but responsive on other days showed a smaller reduction in tuning width across all cohorts (Fig. 4c, right, and Extended Data Fig. 7g), suggesting that the narrowing primarily stems from neurons that were responsive before microablation.

We revisited the increase in neuron pairs with high signal correlations (Fig. 3h,i) by again categorizing neurons by their responsiveness before microablation. In the sound-responsive ablation cohort, neuron pairs that were both responsive before microablation showed a slight decrease in high signal correlations, with no change in other cohorts (Fig. 4d,e). However, pairs of neurons unresponsive on day 5 but responsive on other days showed a marked increase in high correlations 3–9 days after sound-responsive ablation, particularly in pairs with the largest best responses, whereas no change occurred in other cohorts (Fig. 4f,g). Pairs where one neuron was responsive and the other was unresponsive before microablation showed no overall change (Fig. 4h,i). A subset, namely pairs between neurons that maintained responsiveness and previously unresponsive neurons, exhibited a strong increase in high correlations after sound-responsive ablation, absent in other cohorts (Extended Data Fig. 7h,i).

Together, our analysis of the increase in highly correlated neuron pairs, considering their response history, revealed that neurons gaining responsiveness after microablation play a disproportionately large role.

Until now, we examined how microablation affects general neuronal response features, including reliability, responsiveness, amplitude and tuning width. Next, we investigated its impact on specific neuronal tuning in reference to the preablation population response represented by high-category neurons on day 5 (high-category neurons, Fig. 2c and Methods).

For each mouse, we calculated pairwise signal correlations between all spared neurons on a given day, regardless of their responsiveness, and the high-category neurons from day 5. This produced a distribution with a mode near 0, as most neurons were not sound responsive (Extended Data Fig. 7j). We then grouped neurons based on whether they were responsive on day 5 or became responsive on another day. The pairwise correlations in both groups were skewed toward higher signal correlations, reflecting a regional tuning bias. Finally, we binned the signal correlation distributions and normalized to baseline (days 1 and 3).

**Fig. 5:**
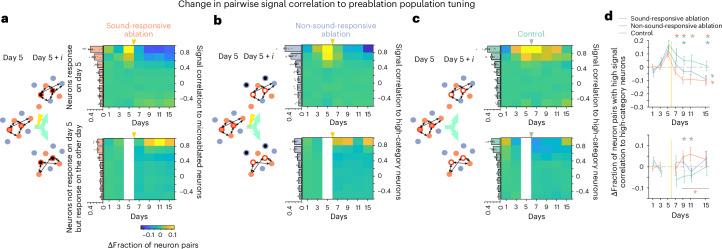
Contrasting shifts in tuning of spared neurons relative to population tuning before microablation. **a**, Tuning shifts in the sound-responsive ablation cohort. Left: scheme of signal correlation analysis with reference to the tuning of highly responsive neurons (high category) on day 5 before microablation (red, responsive neurons; dark red outline, high-category neurons on day 5; blue, unresponsive neurons; black, ablated neurons). Note, signal correlations of spared neurons for a given day were calculated using the tuning of high-category neurons on day 5 (empty circle with dark red outline). Right: color map showing the baseline-subtracted fraction of neurons within each bin of signal correlations for spared neurons responsive on day 5 (top) and neurons not responsive on day 5 but responsive on the other day (bottom). Bar plots next to the color map are fractions of neurons for given signal correlation bins averaged across baseline days (days 1 and 3) in each split group (data are shown as mean ± s.e.m. across mice). **b**, Same as **a** but for the non-sound-responsive cohort. **c**, Same as **a** but for the control cohort. **d**, Baseline-normalized change in fraction of spared neurons with high signal correlation (>0.6) toward high-category neurons (data are shown as mean ± s.e.m. across mice). Top: spared neurons responsive on day 5; data were analyzed by one-way ANOVA over days (asterisk on the right; *P < 0.01; see Supplementary Table 1) and two-sided *t*-test with FDR correction (two-colored asterisks on top; *P < 0.05) between each two of the three experimental cohorts on postablation days. Bottom: spared neurons unresponsive on day 5 but that gained responsiveness on a given day. Data were analyzed by one-sided *t*-test with an FDR correction (two-colored asterisks on top) between each combination of the three experimental cohorts on postablation days (*P < 0.05) and by one-sided *t*-test with FDR correction (asterisk at bottom) between baseline days (days 1 and 3) and late postablation days (days 9, 11 and 15) for a given cohort (*P < 0.05); *n* = 10 mice for sound-responsive ablation, *n* = 10 mice for non-sound-responsive ablation, and *n* = 9 mice for control. Yellow vertical lines indicate the time point of ablation procedure.

In the sound-responsive ablation cohort, we found that fewer neurons that were responsive before ablation retained tuning similar to the ablated high-category neurons, whereas a larger fraction of newly responsive neurons after ablation showed similar tuning (Fig. 5a,d and Extended Data Fig. 7k). The non-sound-responsive ablation cohort showed a weaker effect (Fig. 5b,d). In the control cohort, consistent with an ongoing, basal reformatting of tuning properties, we observed a decline in high signal correlation pairs for previously responsive neurons and a slight increase for newly responsive neurons (Fig. 5c,d).

These analyses suggest that neurons responsive before microablation lose signal correlations to the preablation tuning of high-category neurons, likely due to increased responsiveness loss. By contrast, newly responsive neurons are more likely to develop high signal correlations with the preablation tuning of high-category neurons.

### Ablation induces different effects in excitatory and inhibitory neurons

**Fig. 6:**
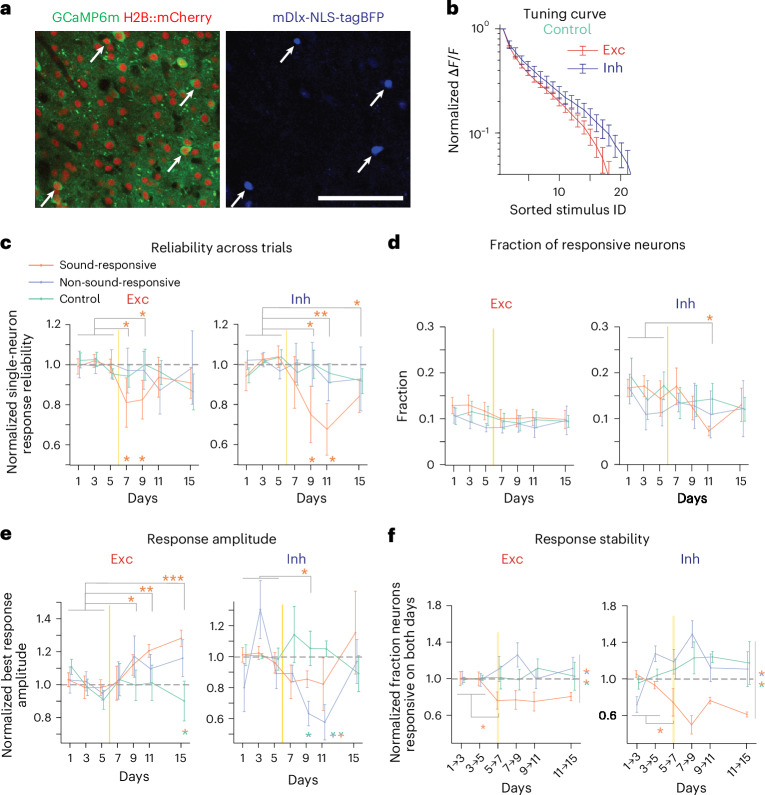
Microablation differentially affects population responses of excitatory and inhibitory neurons. **a**, An FOV in mouse auditory cortex showing broad expression of GCaMP6m and H2B::mCherry (left) and selective expression of nuclear-localized tagBFP in interneurons (right); scale bar, 100 μm. White arrows indicate neurons with mDLx-driven tagBFP expression. **b**, Averaged tuning curves of sound-responsive excitatory (Exc; dark red) and inhibitory (Inh; dark blue) neurons normalized to maximal response during a baseline day in the control cohort (*n* = 7 mice). **c**, Same as Fig. 3a but normalized response reliability across trials for all excitatory (left) and all inhibitory (right) neurons across days in the three experimental cohorts. Data were analyzed by two-sided *t*-test between baseline and postablation days with an FDR correction (top asterisks; *P < 0.05) and permutation test across groups (bottom asterisks; *P < 0.05). **d**, Fraction of sound-responsive excitatory (left) and inhibitory (right) neurons across cohorts. Data were analyzed by two-sided *t*-test with an FDR correction (postablation excitatory neurons: *P* > 0.30 for all cohorts; inhibitory neurons in the sound-responsive cohort: *P < 0.05). **e**, Same as Fig. 3d but normalized best response amplitude of responsive excitatory (left) and inhibitory (right) neurons. Data were analyzed by one-sided *t*-test with an FDR correction (sound-responsive cohort for days 7, 9, 11 and 15: excitatory neurons, *P* = 0.45, 0.045, 1.7 × 10^−3^ and 3.0 × 10^−4^; inhibitory neurons, *P* = 0.053, 0.043, 0.078 and 0.17; top asterisks: *P < 0.05, **P < 0.01, ***P < 0.001) and permutation test comparing ablation cohorts to the control cohort (bottom asterisks; *P < 0.05). **f**, Same as Fig. 4a but normalized fraction of neurons responsive on two consecutive days for excitatory (left) and inhibitory (right) neurons across cohorts. Data were analyzed by two-sided *t*-test comparing overlap during baseline (days 1 → 3 and 3 → 5) versus postablation (days 5 → 7, 7 → 9, 9 → 11 and 11 → 15; *P < 0.05) and by two-sided *t*-test with an FDR correction for group comparisons in the postablation period (right side, two-colored asterisks; *P < 0.01). Data are presented as mean ± s.e.m. across mice for **b**–**f**; sound-responsive ablation (*n* = 5), non-sound-responsive ablation (*n* = 5) and control (*n* = 7), except for permutation tests. Vertical yellow lines indicate the time point of ablation procedure. See Supplementary Table 1 for detailed statistics.

**Extended Data Fig. 8:**
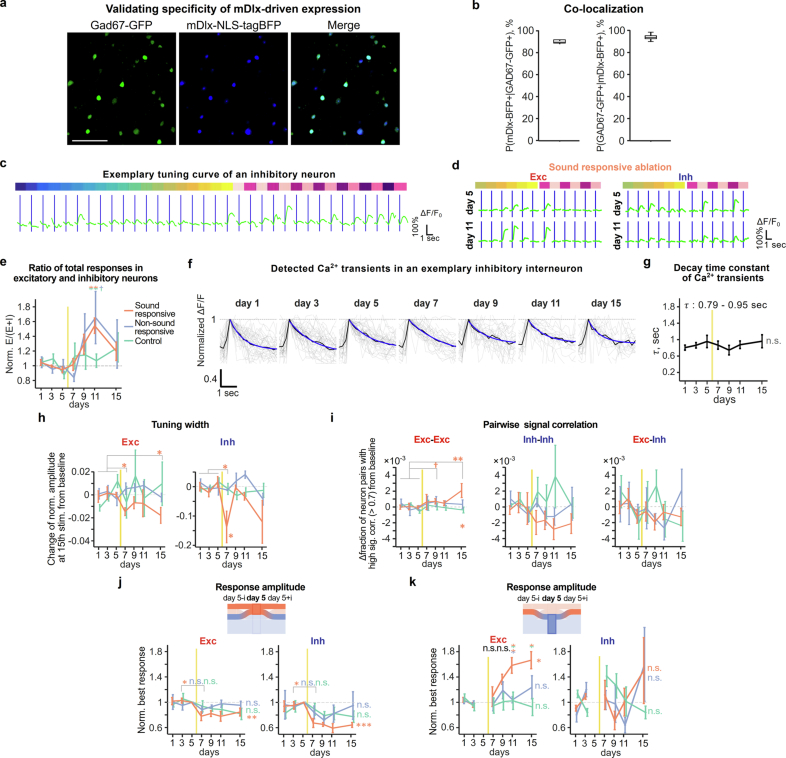
Validation of the viral construct labeling inhibitory neurons and additional analyses of the microablation-induced effects on excitatory and inhibitory neurons. **a**. Representative example of colocalization between mDlx enhancer-driven tagBFP expression and Gad67. Scale: 100 μm. **b**. Quantification of colocalization. *n* = 7 cortical slices from two mice. The middle line, the bottom and top edges of the box plots indicate median, 25th and 75th percentiles, respectively, and the whiskers are the most extreme data points. The fraction of interneurons labeled with the blue fluorescent protein marker out of the entire quality qualified neurons was around 10% for each cohort (10.5 ± 0.5% (mean ± s.e.m. across mice) in sound responsive ablation, 9.8 ± 0.6% in non-sound responsive ablation, and 7.8 ± 0.4% in control, respectively), in line with previous works with auditory cortex in mature adult mice^82^. When we revisited the profile of cell types in microablated neurons, nearly all the ablated neurons were excitatory neurons (92.3 ± 2.0% for sound-responsive ablated neurons, 98.4 ± 0.9% for non-sound responsive ablated neurons), the fraction of which is higher than the average fraction of excitatory neurons in the population. **c**. Exemplary tuning curve of inhibitory neuron. **d**. Representative excitatory (left) and inhibitory (right) neurons, which change response amplitude to sound stimuli from day 5 to day 11, in sound responsive ablation. **e**. Baseline-normalized ratio of the total sound-evoked activity in excitatory and inhibitory neurons (see Methods). Two-sided *t*-test with FDR adjusted *p*-values between experimental cohorts on post-ablation days, * *p* < 0.05.; the normalized ratio of best amplitudes on day 15, 1.09 ± 0.088 for sound responsive ablation, 1.10 ± 0.11 for non-sound responsive ablation. **f**. Traces of detected calcium transients of an inhibitory neuron over days. We verified that the changes of response amplitude in inhibitory neurons after microablation (Fig. 5e) were not due to another feature like Ca^2+^ kinetics in somas of inhibitory neurons characterized by the decay time constant^83^. **g**. Decay time constant of calcium transients of all identified interneurons over days across mice (*n* = 5 for sound responsive ablation cohort). One-way ANOVA test, *p* = 0.82. **h**. Same as Fig. 3f, but change of tuning width by using normalized response amplitudes at 15th largest stimulus index in the tuning curve over days for excitatory (left) and inhibitory (right) neurons. Two-sided *t*-test of Δnormalized amplitude for excitatory neurons (left) and for inhibitory neurons (right) between baseline days vs. days after ablation with FDR correction for each experimental cohort (* *p* < 0.05). **i**. Change in fraction of neuron pairs with high signal correlation and with large response amplitude from baseline among excitatory-excitatory neurons (left), inhibitory-inhibitory neurons (middle), and excitatory-inhibitory neurons (right). Two-sided *t*-test of Δfraction of excitatory-excitatory neuron pairs with FDR correction in sound responsive cohort on post-ablation days (Sound responsive cohort: *p* = 0.15, 0.051, 0.11, 0.0034 for day 7, 9, 11, 15). Asterisks on top: † *p* < 0.055, * *p* < 0.05, ** *p* < 0.01. Permutation test for group comparison (asterisk at bottom; * *p* < 0.05). For inhibitory-inhibitory neuron pairs, sound responsive cohort: *p* > 0.15 for all post-ablation days; non-sound responsive cohort: *p* > 0.28 for all post-ablation days; control: *p* > 0.16 for all post-ablation days. For excitatory-inhibitory neuron pairs, sound responsive cohort: *p* > 0.25 for all post-ablation days; non-sound responsive cohort: *p* > 0.30 for all post-ablation days; control: *p* > 0.26 for all post-ablation days. **j**. Baseline-normalized best response amplitude of neurons responsive on day 5 in excitatory (left) and inhibitory (right) neurons for the three experimental cohorts. When splitting excitatory and inhibitory neurons based on the responsiveness on day 5, as observed in Fig. 4b, the normalized best response amplitude of neurons responsive on day 5 significantly decreased on day 7 for both the excitatory and inhibitory neurons in the sound responsive ablation cohort, but not in the other cohorts (One-way ANOVA across days for excitatory neurons (left) and for inhibitory neurons (right) in each experimental cohort (asterisks at the right side; ** *p* < 0.01, *** *p* < 0.001); Two-sided paired *t*-test of the normalized best amplitude of excitatory neurons (left: Sound responsive ablation, *p* = 0.023) and inhibitory neurons (right: Sound responsive ablation, *p* = 0.049) between day 3 vs. day 7 for each experimental cohort. Asterisks on top; * *p* < 0.05. **k**. Same as **j**, but for newly responsive neurons. For the neurons unresponsive on day 5 but being responsive on the other day, the normalized best response amplitude of excitatory neurons exhibited a delayed but substantial increase after microablation in the sound responsive ablation (One-way ANOVA across days, Sound responsive ablation: *F*(5, 24) = 7.97, *p* = 1.5 × 10^−4^; asterisk at the right side, * *p* < 0.01) like Fig. 4b right, but inhibitory neurons did not (*p* = 0.43). For the non-responsive ablation and the control cohorts, both the excitatory and inhibitory neurons did not change the normalized best response amplitude over days (*p* ≥ 0.24). Group comparison revealed that the normalized amplitude of the newly responsive excitatory neurons in sound responsive ablation was significantly larger than those in non-responsive ablation and in control late after microablation (Two-sided *t*-test with FDR correction on a given post ablation day between each two of the three cohorts; two-colored asterisks on top; * *p* < 0.05). Data presented as mean ± s.e.m. across mice for **e**, **g**-**k**. *n* = 5 mice for sound responsive ablation cohort, *n* = 5 mice for non-sound responsive ablation cohort, *n* = 7 for control for **e**, **h**-**k**. See Supplementary Table 2. for detailed statistics.

The balance of activities in excitatory and inhibitory neurons is known to adjust during neuronal network reorganization^27,35,36^. To assess this in our experiments, we co-injected an AAV vector labeling inhibitory neurons (AAV-mDlx-NLS::tagBFP) alongside AAVs for GCaMP6m and H2B::mCherry in a subset of mice (*n* = 5 sound-responsive ablation, *n* = 5 non-sound-responsive ablation, *n* = 7 control; Fig. 6a). This resulted in ~10% of mCherry-labeled nuclei also expressing blue fluorescent protein (tagBFP). Control experiments in mice expressing GAD67–green fluorescent protein (GAD67–GFP) confirmed highly specific tagBFP expression in GFP^+^ neurons (Extended Data Fig. 8a,b).

As seen in previous studies^37,38^, inhibitory neurons had moderately broader tuning than excitatory neurons during baseline (Fig. 6b and Extended Data Fig. 8c). We then examined whether microablation affected excitatory and inhibitory neurons differently. Similar to Fig. 3a, excitatory neurons showed reduced response reliability across trials after sound-responsive neuron ablation, recovering in later days (Fig. 6c, left). Inhibitory neurons also exhibited reduced reliability, but the effect was delayed and more pronounced (Fig. 6c, right). This suggests that microablation transiently disrupts response reliability in both neuron types, with a stronger impact on inhibitory neurons.

The fraction of sound-responsive neurons was similar between excitatory and inhibitory neurons, with inhibitory neurons showing a slightly higher fraction during baseline (Fig. 6d). Microablation had little effect on the fraction of neurons contributing to the population response, except for a reduction in inhibitory neurons on day 11 (Fig. 6d).

We next examined how microablation affected best response amplitudes in excitatory and inhibitory neurons. In the sound-responsive ablation cohort, excitatory neurons showed an increase, whereas inhibitory neurons exhibited a transient decrease until day 9, normalizing by day 15 (Fig. 6e and Extended Data Fig. 8d). In the non-sound-responsive ablation cohort, inhibitory neurons showed a similar decrease with later recovery, whereas excitatory neurons remained largely unchanged. No significant changes were observed in the control cohort. Analyzing total sound-evoked activity in both neuron types by calculating the product of the fraction of responsive neurons and the average best response amplitude (Methods), we found a temporary shift toward excitatory neurons in both ablation cohorts, which returned to baseline by day 15 (Extended Data Fig. 8e). Calcium transient decay times remained stable, suggesting changes in neuronal activity rather than altered calcium-binding protein expression (Extended Data Fig. 8f,g).

Revisiting microablation-induced changes in tuning width, signal correlations and best response amplitudes (Extended Data Fig. 8h–k), we found no significant differences between excitatory and inhibitory neurons, likely due to the smaller sample size of inhibitory neurons, reducing analysis sensitivity.

We examined the stability of excitatory and inhibitory neuron responsiveness, quantifying again the overlap of sound-responsive neurons on consecutive days. Following microablation of sound-responsive neurons, stability decreased in both cell types, with a more pronounced reduction in inhibitory neurons (Fig. 6f).

Together, our analysis revealed similar microablation-induced effects on response reliability, responsiveness and tuning width in both excitatory and inhibitory neurons, suggesting a general degree of co-tuning. However, network reorganization after microablation led to a shift in sound-evoked responses toward excitatory neurons.

### Prolonged recovery after ablation of inhibitory neurons

**Fig. 7:**
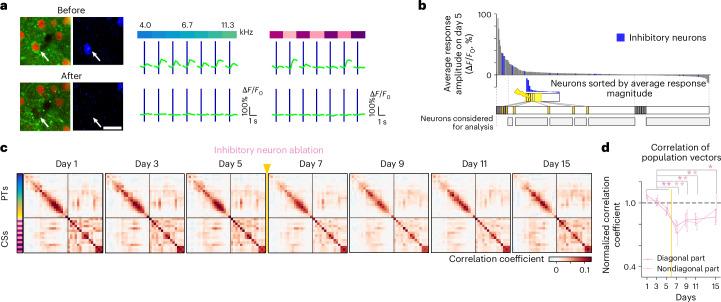
Targeted microablation of inhibitory neurons causes a longer-lasting disturbance of the representational map. **a**, Representative example of microablation of a targeted inhibitory neuron with PT and CS tuning. Left: interneuron targeted for microablation (white arrow) showing expression of GCaMP6m (green), H2BB::mCherry (red) and mDlx-driven tagBFP (blue); scale bar, 20 μm. Middle: calcium responses to various pure tones before and after microablation. Right: calcium responses to various complex sounds before and after microablation. **b**, Target neurons for inhibitory neuron microablation. Top: sorted sound-evoked amplitudes before microablation in an FOV. Interneurons are labeled as blue bars, and excitatory neurons are labeled as gray bars. Middle: sorted amplitudes of the same population above but only from interneurons. Four to eight highly responsive interneurons were targeted per FOV. Bottom: experimentally targeted neurons and neurons corresponding to target groups in responsive and nonresponsive cohorts were excluded from analysis. Analysis was conducted on the remaining spared neurons (gray bars) **c**, Similarity matrix of population vectors for all stimuli averaged across all FOVs for each day in the inhibitory neuron ablation cohort (*n* = 42 FOVs). **d**, Baseline-normalized correlations averaged across diagonal elements (dark pink) and nondiagonal elements (light pink) in the similarity matrices in **c** (data are shown as mean ± s.e.m. across six mice). Data were analyzed by two-sided *t*-test of normalized average correlation between baseline days and days after ablation with an FDR correction (*P < 0.05 and **P < 0.001) and by one-way ANOVA of normalized average correlation across days in diagonal elements (*P* = 3.3 × 10^−3^) and nondiagonal elements (*P* = 0.068; see Supplementary Table 1). Yellow vertical lines indicate the time point of ablation procedure. For comparison, the analogous effect of sound-responsive ablation of all neurons (mostly excitatory) is depicted in Fig. 2. Source data

Interneurons are crucial for cortical network stability and plasticity^35,36,39^. To assess the impact of selectively removing inhibitory neurons, we conducted an experiment again identifying these cells using the mDlx-driven blue fluorescent reporter (*n* = 6 mice; Fig. 7a). Unlike previous cohorts, where mostly excitatory neurons were ablated, we focused on sound-responsive inhibitory neurons. Due to their lower density (~10% of the local population; Extended Data Fig. 8a), we also ablated some nonresponsive inhibitory neurons to match the number removed in other cohorts (Fig. 7b, Extended Data Fig. 4a–e and Methods).

**Extended Data Fig. 9:**
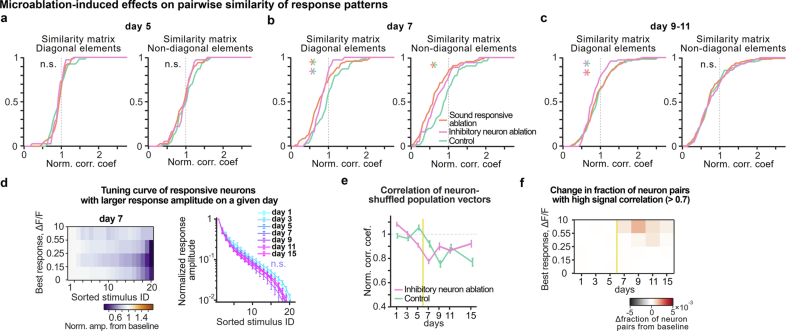
Additional analyses of the effects induced by selective microablation of inhibitory neurons on the representational map and response characteristics of individual neurons. **a**. Cumulative distribution of baseline normalized correlation coefficient on day 5 in the representational map for each experimental cohort (*n* = 42 and 59 FOVs for inhibitory neuron ablation and control cohorts, respectively). In inhibitory neuron ablation experiment, 91.7 ± 1.5% was successfully ablated out of the targeted neurons, based on the criteria we already described in Methods. Same as Extended Data Fig. 5a, to compare the effect of ablation between inhibitory neuron ablation and sound responsive ablation cohorts on the day after microabtaion, difference in these cumulative distributions was tested by Dunn’s test as well as two-sided Mann-Whitney *U* test with FDR correction. These cumulative distributions on day 5 were not statistically different by definition between the groups (Dunn’s test for all group combinations in both diagonal and non-diagonal elements, Q < Qcritical; two-sided Mann-Whitney *U* test, adjusted *p* values > 0.5). **b**. Same as **a**, but the cumulative distributions of normalized correlation on day 7. Especially for diagonal elements, the distributions in both sound responsive ablation and inhibitory neuron ablation cohort shifted toward lower correlation than control (Two-sided Mann-Whitney *U* test, adjusted *p*-values between experimental cohorts; two-colored asterisks, Diagonal elements: Sound responsive ablation vs. control: *p* = 0.016; Inhibitory vs. control: *p* = 0.020; Sound responsive ablation vs. inhibitory: *p* = 0.51; * *p* < 0.05; Dunn’s test, sound responsive ablation vs. control, Q = 3.00 (> Qcritical); inhibitory neuron ablation vs. control, Q < Qcritical). For non-diagonal elements, the distributions in sound responsive ablation cohort was lower than that in control (Two-sided Mann-Whitney *U* test, with adjusted *p* values between experimental cohorts; two-colored asterisks, * *p* < 0.05; Dunn’s test, sound responsive ablation vs. control, Q = 3.48 (> Qcritical); Q < Qcritical for the other group comparison). **c**. Same as **a**, but during day 9–11. On the other hand, late after microablation (day 9–11), while the distribution in sound responsive ablation went back to baseline level, the distribution in inhibitory neuron ablation, especially the diagonal elements maintained lower correlation than the other cohorts (Dunn’s test, inhibitory neuron ablation vs control, Q = 2.44 (> Qcritical); sound responsive ablation vs. inhibitory neuron ablation, Q = 2.43 (> Qcritical); two-sided Mann-Whitney *U* test, adjusted *p*-values between experimental cohorts, *p* ≥ 0.056). **d**. Same as Fig. 3e, but normalized tuning curve of responsive neurons in inhibitory neuron ablation. Left: the colormap of the baseline normalized tuning curve on day 7 according to the best response amplitude bins. Right: normalized tuning curves overlaid across days (mean ± s.e.m. across mice). Two-sided *t*-test of average of normalized amplitudes across the stimulus with 2nd largest amplitude to the last stimulus between baseline days vs. day 7: *p* = 0.20. **e**. Same as Fig. 3g, but normalized non-diagonal correlations in the similarity matrix constructed from the population vectors shuffled across FOVs (Methods) in inhibitory neuron ablation (mean ± s.e.m. across mice). Two-sided *t*-test of normalized correlation during all post-ablation days between inhibitory neuron ablation and control. *p* = 0.33. **f**. Same as Fig. 3h, but the colormap of the change in fraction of responsive neuron pairs with high signal correlation from baseline according to the best response amplitude in inhibitory neuron ablation cohort (*n* = 6 mice). Two-way ANOVA, *p* = 0.062 across days; *p* = 0.0022 across amplitude bins. *n* = 6 mice for inhibitory neuron ablation cohort for **d**-**f**. See Supplementary Table 2. for detailed statistics.

Removing inhibitory neurons disrupted the representational map, similar to the sound-responsive ablation cohort (Fig. 7c). However, while recovery occurred within 3 days in the latter, it was notably delayed in the inhibitory neuron ablation cohort (Fig. 7c,d and Extended Data Fig. 9a–c).

**Fig. 8:**
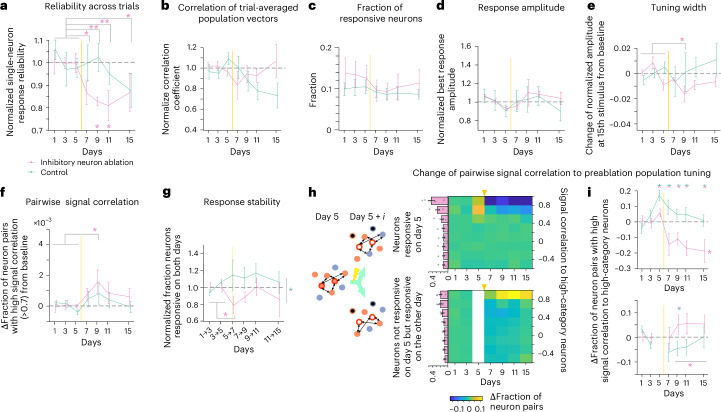
Single-neuron properties following inhibitory neuron ablation. **a**, Baseline-normalized response reliability across trials of single neurons, averaged across all neurons per day (pink, inhibitory neuron ablation cohort; green, control cohort). Data were analyzed by one-way ANOVA (*P* = 0.027), two-sided *t*-test between baseline and postablation days with an FDR correction (top asterisks; *P < 0.05 and **P < 0.01) and permutation test for group comparisons (bottom asterisks; *P < 0.05). **b**, Baseline-normalized correlations averaged across nondiagonals in the similarity matrix constructed from trial-averaged population vectors. Data were analyzed by one-way ANOVA (*P* = 0.66), two-sided *t*-test with an FDR correction (*P* > 0.26) for all postablation days and permutation test (*P* > 0.21) for all postablation days. **c**, Fraction of sound-responsive neurons over days (one-way ANOVA, *P* = 0.90). **d**, Baseline-normalized best response amplitude of neurons responsive for each day. Data were analyzed by one-way ANOVA (*P* = 0.47), two-sided *t*-test (*P* > 0.13 for all postablation days) and permutation test (*P* > 0.13 for all postablation days). **e**, Change in normalized response amplitudes from baseline at the 15th largest stimulus index in the tuning curve (Extended Data Fig. 9d). Data were analyzed by two-sided *t*-test with an FDR correction (*P* = 0.34, 0.043, 0.17 and 0.34 for days 7, 9, 11 and 15; *P < 0.05) and permutation test (*P* > 0.085 for all postablation days). **f**, Change in fraction of neuron pairs with high signal correlation in the largest best response category (Extended Data Fig. 9f). Data were analyzed by two-sided *t*-test with an FDR correction (*P* = 0.079, 0.017, 0.10 and 0.15 for days 7, 9, 11 and 15; *P < 0.05) and permutation test (*P* > 0.17 for all postablation days). **g**, Response stability as the fraction of neurons categorized as sound responsive on two consecutive days. Data were analyzed by two-sided *t*-test: baseline (1 → 3, 3 → 5) versus postablation (5 → 7), *P* = 0.026; inhibitory neuron ablation versus control during 7 → 9 and 9 → 11, *P* = 0.048. *P < 0.05. **h**, Tuning shifts in the inhibitory ablation cohort relative to preablation population tuning. Left: same scheme as Fig. 5 but inhibitory neurons were ablated (black). Right: color map of baseline-subtracted fractions of spared neurons within each bin of signal correlations with day 5 high-category neurons (top: day 5 responsive spared neurons; bottom: spared neurons unresponsive on day 5 but responsive on other days). The bar plots next to the color map are baseline-averaged fractions of spared neurons in each split group (see Fig. 5). **i**, Baseline-subtracted change in fraction of spared neurons with high signal correlation (>0.6) toward high-category neurons in inhibitory neuron ablation and control cohorts. Top: day 5 responsive spared neurons. Bottom: spared neurons unresponsive on day 5 but responsive on other days. Data were analyzed by two-sided *t*-test: ablation versus control (top: *P* = 0.025, 6.1 × 10⁻^3^, 5.3 × 10⁻^3^, 2.8 × 10⁻^3^ and 0.018 for days 5, 7, 9, 11 and 15; bottom: *P* = 0.35, 0.028, 0.072 and 0.24 for days 7, 9, 11 and 15, respectively). Top asterisks, *P < 0.05. Bottom, data were analyzed by *t*-test between baseline and days 9–15 (bottom asterisk; *P < 0.05). Control cohort data were replotted for comparison; refer to Figs. 3a–f, 4g and 5h–i. Data are presented as mean ± s.e.m. across mice; inhibitory neuron ablation *n* = 6 and control *n* = 9, except for permutation tests. See Supplementary Table 1 for full statistics.

Next, we analyzed the single-neuron response properties associated with the removal of inhibitory neurons. Removing inhibitory neurons led to a sustained reduction in response reliability, unlike the transient drop seen in the sound-responsive ablation cohort (Fig. 8a). However, tuning properties remained largely unchanged, as indicated by stable nondiagonal correlations in the similarity matrix constructed from trial-averaged population responses (Fig. 8b). Thus, inhibitory neuron ablation primarily affects response reliability rather than tuning properties.

Consistently, repeating our analyses in the inhibitory neuron ablation cohort revealed no tuning property changes comparable to the sound-responsive ablation cohort (Fig. 8c–f (fraction of responsive neurons, best response amplitudes, width of tuning and fraction of pairs of neurons with high signal correlations, respectively) and Extended Data Fig. 9d–f). However, microablation of inhibitory neurons led to a reduced overlap of responsive neurons across days, indicating an increased drift in responsiveness (Fig. 8g).

We next again analyzed signal correlations of spared neurons on a given day to those neurons central in forming the population response before microablation, that is, the high-category neurons. Responsive neurons on day 5 showed a marked drop in high signal correlations (Fig. 8h,i, top), whereas neurons gaining responsiveness had an increased likelihood of high signal correlations (Fig. 8h,i, bottom).

Together, we observed that the targeted ablation of different neuron classes from the network caused specific changes in single-neuron properties at multiple timescales that underlie a transient impairment of the representational map, but also its subsequent recovery.

## Discussion

We used the representational map, formed by the population responses to a set of auditory stimuli, as a readout of auditory cortical function^1,2^. Despite stable environmental and behavioral conditions, individual neurons exhibited substantial volatility in their tuning properties, consistent with previous findings in the auditory cortex^6,32^ and other brain regions^4,7,10–12^. Notably, the relative distances between stimuli in the neuronal coding space remained unchanged, preserving the overall structure of the representational map^4,40–42^. Because the total responsiveness summed over all neurons remained stable, these changes likely reflect rotational shifts in the representation’s geometry^34^.

**Extended Data Fig. 10:**
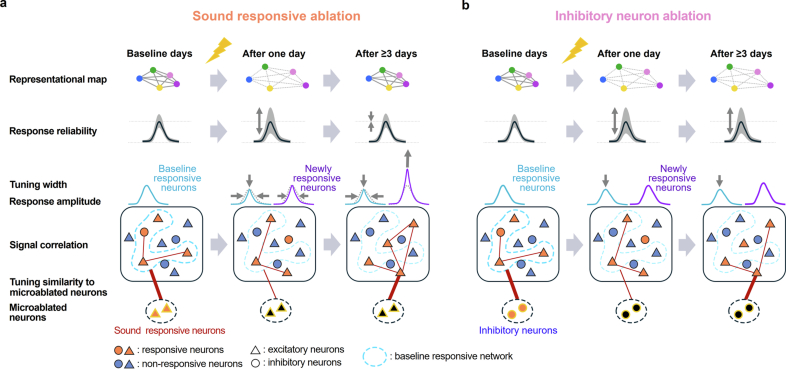
Schematic summary of homeostasis in the representational map and the underlying single-neuron mechanisms. **a**. Schematics in ablation of sound responsive neurons. After microablation of sound responsive neurons, the representational map undergoes temporal disturbance and recovery in 3–5 subsequent days. Here the representational map is depicted as the colored dots corresponding to different sound identities in representational space from population activities (top row). Longer distance between dots in this space indicates lower correlation that is, dissimilar relationship of population activities between the sounds. Response reliability across trials in individual neurons is reduced on the day after ablation shown as the example tuning curve with a shaded gray band (the 2^nd^ row). The broader band indicates a larger variability in responses across trials. The width of tuning curve in the individual neurons is reduced on the day after ablation and remains narrowed over several days (the 3^rd^ row). The narrowing of the width is more pronounced in baseline responsive neurons (the 3^rd^ row, light blue curve). The average best response amplitude for the baseline responsive neurons keeps reduced after microablation (the 3^rd^ row, light blue curve), while the best amplitude for the newly responsive neurons, which are unresponsive on the day before ablation but become responsive on any other day, increased a few days after microablation (the 3^rd^ row, purple curve). The rate of response turnover, where some responsive neurons lose their responsiveness and other unresponsive neurons gain their responsiveness, is accelerated after microablation. The more neurons in the baseline responsive network (rounded squares in the 4^th^ row, light blue dotted circle) lose their responsiveness, the more neurons outside the baseline network gain responsiveness. The fraction of neuron pairs with high signal correlation, that is, highly similar tunings increase later days after microablation (the number of red edges in the population inside the rounded squares in the 4^th^ row). The increase of fraction is specifically driven by the newly responsive neurons, which are located outside of the baseline responsive network. Similarity in tuning between the microablated neurons and the spared neurons increased later days after microablation, especially between the microablated neurons and the newly responsive neurons (bottom, shown as the thickness of red edges between these neurons). **b**. Same as **a**, but in ablation of inhibitory neurons. Different from microablation of sound responsive neurons, microablation of inhibitory neurons induces long-lasting disturbance of the representational map, which is depicted as the more widely distributed color dots in representational space (top row). The single-neuron response reliability across trials maintains a lower level than the baseline level (the 2^nd^ row) with the broader shaded gray band. Since the reduction of similarity matrix is mostly due to the destabilization of response across trials, the tuning width (the 3^rd^ row), best response amplitude (the 3^rd^ row) and tuning similarity between neurons (rounded squares in the 4^th^ row) do not change after microablation. Interestingly, the tuning similarity between the microablated neurons (bottom, dotted circle) and the newly responsive neurons increases also for inhibitory neuron ablation.

To examine the impact of neuron loss on network function, we selectively removed 30–40 sound-responsive neurons. We found that this manipulation was sufficient to transiently disrupt the correlational structure of the population responses underlying the representational map. Linking this global change in sensory representation to properties of individual neurons^2,34^, we delineated the contributors to the disruption as well as the homeostatic recovery of the map. The removal of excitatory neurons reduced response reliability and led to long-lasting tuning narrowing, but recovery occurred within 3 days, driven by newly responsive neurons with highly correlated tuning (Extended Data Fig. 10a). By contrast, inhibitory neuron removal caused a more prolonged disturbance, primarily linked to a sustained decline in response reliability, delaying recovery beyond 5 days (Extended Data Fig. 10b)^43,44^.

In recent years, the role of interneurons in shaping sensory responses has been explored through acute optogenetic manipulation of specific subclasses^45–47^. However, direct comparisons to our study are challenging, as we used the mDlx enhancer to broadly label interneurons for microablation and assessed effects only after ~24 h (ref. ^48^). Notably, studies on genetic mouse models with developmental interneuron loss, similar to our findings, did not show general disinhibition but rather mildly narrowed tuning and decreased excitatory responses^49,50^.

Analyzing signal correlations between spared neurons and neurons that strongly contributed to the population response (high-category neurons) before microablation revealed contrasting effects in preablation responsive neurons and those gaining responsiveness. In particular, the removal of highly responsive neurons or the removal of inhibitory neurons led to a reduction of preablation responsive neurons with high signal correlations, likely due to increased rates of losing responsiveness to sounds. Meanwhile, neurons gaining responsiveness were more likely to develop tuning similar to high-category neurons before ablation.

These observations suggest two possible processes. First, the loss of signal correlation in preablation responsive neurons may indicate destabilization, as their tuning relies on recurrent interactions within the subset. The loss of members may disrupt this network similar to reports in the somatosensory cortex after touch neuron ablation^21^. Second, the increase in signal correlations in neurons gaining responsiveness may reflect the latent potential of some unresponsive neurons to adopt tuning similar to highly responsive neurons. These neurons might be suppressed by inhibitory circuits and only become sound responsive when this inhibition is lifted. The slow recovery of the representational map over several days suggests that this disinhibition involves slower plasticity mechanisms beyond acute alterations in synaptic inputs.

The high sensitivity of the similarity matrix to the removal of a small number of neurons suggests that the apparent stability of the representational map under basal conditions does not stem from a redundant or intrinsically stable network. Instead, it relies on plasticity mechanisms continuously maintaining functionality^29,51–54^. Although these mechanisms effectively manage ongoing tuning changes under normal conditions, they can be temporarily challenged by the rapid loss of specific neurons. Similar selective changes in circuit function and behavior due to subtle neuronal manipulations have been observed in various contexts^21–24,33^.

As outlined above, theoretically, three major scenarios were conceivable regarding the long-term dynamics of the map after its disruption: (1) an irreversible impairment due to the permanent loss of neurons (no compensation), (2) gradual mitigation through ongoing tuning volatility (passive compensation) or (3) engagement of homeostatic processes to restore the map (active homeostasis). Our findings, showing map recovery alongside specific changes in single-neuron properties, are inconsistent with scenarios 1 and 2. Instead, the delayed increase in signal correlations (Fig. 3h) and the shift in excitatory/inhibitory response amplitudes (Fig. 6e) align with scenario 3. Similar changes in inhibition have been observed in other scenarios when cortical circuits adapt to activity silencing or sensory perturbations^36,55–58^. However, because tuning changes from day to day occurred at similar rates after excitatory and inhibitory neuron ablation but were associated with different recovery times, their exact role in homeostatic recovery remains unclear.

Within the homeostasis framework, the dynamic network reorganization following neuron removal suggests that the correlational structure of the representational map may itself be a controlled variable being returned to its set point^30^. Here, single-cell changes act as the effectors shaping the representational map. The finding that the ablation of selected neurons, a permanent challenge to the representational map, induces changes in the effectors that outlast the map’s recovery is expected, as they typically do not operate in parallel with the controlled variable^59^. Considering much longer periods after the ablation beyond our observation period, however, ongoing neuronal tuning changes would likely restore single-cell properties to baseline. Future investigations of homeostatic mechanisms preserving higher-order brain functions will be of interest^28^, as classic homeostatic models based on firing rate fail to explain regulation across network scales^60,61^. Furthermore, the nature of a putative control center eventually computing a deviation from the set point to activate effectors is still elusive.

We propose that the ability to compensate for the loss of microablated neurons by the selective recruitment of previously unresponsive neurons at the microcircuit level may also serve as a mechanism by which brain function may be upheld for extended periods of neuronal loss during aging and prodromal stages of neurodegenerative disease^17,18^.

## Methods

All animal experiments were performed in accordance with the German Laboratory Animal Law guidelines for animal research and were approved by the Landesuntersuchungsamt Rheinland Pfalz (approval 23 177-07/G 17-1-051).

### Animals

Experimental subjects were male 4- to 6-week-old C57BL/6JRj mice obtained from Janvier Laboratories. A male and a female mouse from the transgenic strain expressing GAD67–GFP were obtained from a breeding in-house. Before surgical procedures, mice were kept in groups of four and housed in 530 cm^2^ cages on a 12-h light/12-h dark cycle (temperature of 22 ± 2 °C and relative humidity of 55 ± 10%) with unlimited access to dry food and water. Experiments were performed during the light period.

### rAAV cloning and production

For the rAAV genome encoding GCaMP6m^62^ under the human *SYN1* promoter and the rAAV encoding H2B–mCherry, two plasmids were generated as described before^6^. To elaborate the plasmid containing a gene encoding tagBFP, preceded by three nuclear localization signals under the mDlx enhancer sequence (pAAV-mDlx-NLS-tagBFP), we started from the commercially available plasmid AAV-mDlx-NLS-mRuby2 (Addgene plasmid 99130) and a plasmid for tagBFP in our lab stock (TRE-NLS-tagBFP, which is modified from Addgene plasmid 92202). The NLS-tagBFP sequence was PCR amplified from plasmid pAAV-TRE-NLS-tagBFP. The resulting PCR product was digested with the respective enzymes and purified. From AAV-mDlx-NLS-mRuby2, NLS-mRuby2 was excised with the restriction enzyme, and the NLS-BFP sequence was ligated into the cut and purified. The integrity of the final plasmid pAAV-mDlx-NLS-tagBFP was confirmed by Sanger sequencing. All plasmids described above were packaged into AAV8 capsid as previously described^63^. Constructs used in this study are available upon request from the authors.

### Stereotaxic injections and cranial window implantation

We followed the procedures as previously described in detail^6^. Mice were anesthetized using isoflurane (maintained at 1.2–1.5%) and mounted on a stereotaxic device. Injections were performed perpendicular to the surface of the skull. The virus solution consisted of a mixture of two different rAAVs (rAAV8-hSyn-GCaMP6m-WPRE-hGHpolyA; titer: 1.35 × 10^11^ viral genomes (vg) per ml; rAAV8-hSyn-H2B–mCherry-hGHpolyA; titer: 2 × 10^13^ vg per ml) in PBS. For another subset of experiments, rAAV8-mDlx-NLS-tagBFP-WPRE-hGHpolyA (titer: 2.16 × 10^11^ vg per ml) was additionally mixed with the foregoing rAAVs. In total, 175 nl of the virus mixture loaded into a glass pipette was injected in five locations (20 nl min^–1^, total volume of 875 nl) along the anterior–posterior axis with coordinates 4.4, −2.3/−2.6/−2.9/−3.2/−3.5, 2.5 (in mm, lateral, caudal, and ventral to bregma) to cover the right auditory cortex. Several days after the injection, mice were again anesthetized using isoflurane (maintained at 1.2–1.5%) and mounted on a stereotaxic device with a custom-made v-shaped head holder. After the parietal and temporal bones were exposed, the skull above the auditory cortex (2 × 3 mm) was gently drilled, and the bone was carefully lifted. The craniotomy was covered with a small cover glass fixed with dental cement. To position the window plane perpendicular to the objective under the microscope, a custom-made titanium head post was mounted on the frontal bone and embedded with dental cement. After the surgical procedure, animals were single housed and recovered for at least 1 week before further handling.

### Habituation to awake chronic two-photon imaging

We habituated mice to handling at the two-photon microscope. Mice were fixated under the objective in a custom-made acrylic glass tube using a custom-made head post. The mouse head was laterally tilted such that the surface of the auditory cortex aligned with the horizontal plane. Habituation was repeated for at least 7 days, the duration of which increased from around 10 min on the first day to around 2 h on the last day, comparable to the duration of two-photon imaging in 1 day. Habituation ended when mice were accommodated to the head fixation apparatus. Because the sound stimulus set for longitudinal two-photon imaging was presented dozens of times each day, animals repeatedly experienced all sound stimuli before data acquisition.

### Intrinsic imaging

We identified the functional organization of auditory fields to set the location of calcium imaging within the fields via intrinsic signal imaging. We followed a previously reported protocol^32^. Mesoscopic optical imaging was conducted using a CCD camera (Vosskuehler, CCD1200QD; frame rate of 25 Hz) with macroscope objectives. Intrinsic signals were recorded at 400 μm below the brain surface from near-infrared (780 nm) LEDs under isoflurane anesthesia (concentration of 1.0–1.2%), while presenting 18 PT pips (80-ms-long individual pips with 20-ms smooth gaps; 1.8 s of the total stimulus duration) with different tone frequencies (1, 2, 4, 8, 16, 32 and 64 kHz) and white noise bursts in 30 randomized trials. The sound intensity was set at a sound pressure level of 70 dB. The change in light reflectance between pre- and poststimulus images was computed and averaged across trials.

### Sound presentation

All sounds were delivered free field by a ribbon loudspeaker a distance of 25 cm from the mouse head in a soundproof booth. The stimulus set consisted of 34 sound stimuli (19 PT pips (50 ms; 2–45 kHz separated by a quarter octave) and 15 CSs (70 ms)) separated by a 1-s interval, as described in the previous study^6^. The CSs were generated from arbitrary samples of music pieces or animal calls replayed at fourfold speed. In a subset of mice across all experimental cohorts, a set of nine conspecific vocalizations (<80 ms) was presented during habituation and during imaging but was not included for further analyses in this study. All stimulus onsets and offsets were smoothened by a cosine ramp function with a duration of 10 ms, respectively. The stimulus set was presented with ten repetitions per stimulus in a pseudorandom order for each FOV at a sound pressure level of 70 dB.

### Two-photon imaging

The imaging configuration and procedure have been described in detail previously^6^. Two-photon calcium imaging was performed with a commercial microscope (Ultima IV, Bruker Corporation) with a ×20/0.95-NA objective (XLUMPlan Fl, Olympus) and a tunable pulsed laser (Chameleon Ultra, Coherent) in a soundproof chamber. Both GCaMP6m and mCherry were coexcited at a wavelength of 940 nm and separated by emission using a filter cube (U-MSWG2, Olympus). tagBFP was excited at 810 nm and separated by another filter cube (U-MWIB3, Olympus). Time series imaging was performed using an FOV of 367 × 367 μm (pixel size: 256 × 128) at a frame rate of 5 Hz. In a daily session, imaging was performed not only with presentation of the sound set but also without any sound presentation to record spontaneous neuronal activities for 144 s in a given FOV. In the longitudinal imaging experiments, six to eight FOVs were sequentially imaged per mouse along the layer 2/3 column from deeper to shallower depths (a depth of ~110–325 μm from the cortical surface). FOVs with reliable sound responses were repeatedly imaged at a 2-day interval, except for the last imaging day (4-day interval between the sixth and seventh time points). Imaging days were counted according to the first day of longitudinal data acquisition in both laser ablation and control experiments. *Z*-stack images of the layer 2/3 column were also acquired at different time points (before starting the experiment, just before and after microablation and after finishing the imaging experiment).

### Protocol, quantification and validation of microablation

Targeted neurons were identified from a full-frame scan. We switched to point scan mode with a dwelling time of 8 μs, and scan position was set at the center of the nuclei of the neurons. Microablation was performed with a wavelength of 760 nm and femtosecond laser pulses (140 fs) delivered through a ×20/0.95-NA objective to mice that were lightly anesthetized with isoflurane (0.8–1.3%). The laser power was set between 100 and 220 mW according to the quality of fluorescence signal in the target neuron. We applied the pulsed laser and immediately terminated on the detection of the abrupt elevation of calcium fluorescence. Otherwise, we kept the laser application for up to 30 s. If we observed clear elevation of Ca^2+^ signal within 1 min after laser exposure, we determined the procedure effective. We took an iterative approach for ablation to increase the success rate and prevent off-target damage to the surrounding region at the same time (Extended Data Fig. 3a). If we did not observe such a calcium elevation, we repeated the procedure with a slight increase in laser power at most three times. If the targeted neuron still did not show any observable increase of calcium fluorescence, we waited ~20–30 min and again applied the laser once more. When the last application did not induce an elevated calcium signal, we categorized the neuron as procedure ineffective. As longer exposure times tended to produce off-target effects^33^, whenever we observed abnormal calcium increases or strong bleaching in the surrounding region (~50 μm in radius) during the iteration, we halted the laser application and categorized the neuron as procedure ineffective. If accumulated laser ablation caused massive damage to the surrounding regions (~>100 μm in radius) of the target neurons, we terminated the experiment and categorized it as a failure. Given that prolonged application of laser light increased the chance of nonspecific photodamage in the tissue and our procedure involved individual targeting and manual ablation of each neuron, typically taking several minutes, in practice, a maximum number of 30–40 neurons per mouse could be microablated in a single session.

We conducted in vivo and ex vivo experiments to evaluate the microablation protocol. To keep tracking neurons across days in vivo, we took a single image of each FOV with a size of 367 × 367 μm (pixel size: 512 × 512) for each imaging session. We evaluated the effect of microablation by taking *z*-stack images of the layer 2/3 column, including the targeted neurons, and compared images of the nucleus and somatic fluorescence between the time points just before and after microablation, after 2 and 4 days, and quantified the loss of nuclear and calcium signal in target neurons as well as in the neurons included in a cubic volume of 60 × 60 × 60 μm around the targeted neurons to quantify off-target effects. The criterion for successful ablation of target neurons was (1) a complete loss of nuclear fluorescence and abnormality (complete loss or excessive filling) of calcium somatic fluorescence or (2) a partial loss of nuclear signal with excessively filled calcium fluorescence in the soma. On the other hand, we classified a neuron as noneffectively ablated when the loss of nuclear fluorescence was unclear or when the loss was partially observed but with intact calcium somatic fluorescence.

After 4 days of in vivo *z*-stack imaging, mice were anesthetized, the cranial windows were carefully removed, and Evans blue (Sigma-Aldrich, E2129-10G) was injected at the corners of the imaged region on the cortical surface so that the blue dye served as a landmark of the imaged region after tissue extraction. Mice were perfused with a PBS/heparin solution and subsequently with a 4% paraformaldehyde solution following standard procedures. To keep the *z*-stack imaged volume intact as much as possible, the bulk brain section over 600 μm thickness was cut roughly parallel to the two-photon imaging plane on a vibratome (VT-1000, Leica Biosystems). To enable confocal imaging of the bulk section, the section was cleared using the CUBIC protocol^64^, washed with PBS and incubated in a DAPI solution (10 μg ml^–1^) for 24 h. Images were then acquired on a confocal microscope (DMi6000B CS TCS SP5, Leica) using a ×20/0.7-NA dry objective (HCX PL APO CS) with a frame size of 553.6 × 553.6 μm (pixel size: 1,024 × 1,024) as a *z* stack with a resolution of 0.5 μm. The region for confocal imaging was estimated by the blue dye landmarks. By slightly scaling and rotating roll, pitch and yaw of the post hoc three-dimensional confocal image, the in vivo-imaged population and microablated neurons were identified in the post hoc image data.

### Validation of AAV vector labeling of inhibitory interneurons in vivo

rAAV8-mDlx-NLS-tagBFP was injected into mice expressing GAD67–GFP. Three weeks after injection, the mice were deeply anesthetized with ketamine/medetomidine (2.5 mg /0.02 mg per 25 g body weight), perfused with 10 ml PBS/heparin and switched to 10 ml of 4% paraformaldehyde solution using the standard protocol. Brains were extracted and sliced on a Vibratome (VT-1000) to generate 100-μm coronal sections, and sections were mounted on cover slips. Confocal images were acquired (DMi6000B CS TCS SP5) using a ×20 dry objective (HCX PL APO CS). From the images, colocalization of GFP and tagBFP signal was evaluated by custom-written MATLAB scripts.

### Data analysis

#### Image processing pipeline for chronic two-photon data

Before applying an individual neuron tracking algorithm, global *x–y* image displacement induced by movement was corrected by a cross-correlation-based method^65^. Regions of interest (ROIs) were semiautomatically selected by a custom-made MATLAB program, which were manually corrected later by a human expert and described by a set of several hundred points marking the centers of neuronal nuclei. To track individual ROIs across days, we followed a procedure described in previous studies^6,66^. Generally, two-dimensional affine transformation was optimally applied to register ROIs in each frame of the time series from the same FOV across several days. Optimization of the transformation was achieved using a Nelder–Mead–Simplex algorithm with a MATLAB function (fminsearch) and by iterating it in two different spatial scales, which first covered the entire frame and second focused on the four equally split image segments to adjust local movements during two-photon scanning. Last, to adjust to further local distortion, individual ROIs were allowed to move up to 2 pixels (2.87 μm) and find a better match with the image.

#### ROI inclusion criteria and calculation of Δ*F*/*F*_0_ deconvolution

To include only neurons in the analysis that had a reliably present nuclear signal in the H2B:mCherry channel, we applied four quality criteria described in our previous study^6^: nearest neighbor distance, normalized soma signal intensity, soma signal-to-noise ratio and objective function value, which were applied on a frame-by-frame basis so that a neuron was either reliably present or excluded at each time point. To correct fluctuating background fluorescence from somatic calcium signal, the out-of-focus neuropil signal was calculated as the average fluorescence value of an area surrounding each individual ROI and was subtracted from the time series after multiplication with a contamination ratio as described previously^67,68^. We calculated a contamination ratio independently for each imaging plane in each experiment^67^. To calculate Δ*F*/*F*, the baseline *F*_0_ value used to compute Δ*F*/*F*_0_ was defined as a moving rank order filter, the 30th percentile of the 200 surrounding frames (100 before and 100 after). To estimate the neuronal firing rate, this Δ*F*/*F*_0_ value was then deconvolved using the conventional algorithm^69^.

#### Stimulus-evoked sound responsiveness of single neurons

To classify single neurons as sound responsive or not, all ten trials from a given stimulus were compared in a signed-rank test against ten prestimulus spontaneous activities. A neuron was classified as significantly responsive if the *P* value was below 0.05 after a Benjamini–Hochberg (or FDR) correction for multiple comparisons against number of stimuli (34) for at least 1 stimulus^6^. The amplitude of sound-evoked calcium transients was calculated by subtracting the average prestimulus baseline Δ*F*/*F* value (two frames corresponding to –200 to 0 ms) from the average poststimulus peak Δ*F*/*F* value (two frames corresponding to 200–400 ms) of all trials^67^. We defined best response amplitude as the average response amplitude across trials elicited by the stimulus giving rise to the significant maximal response among the 34 stimuli.

#### FOV inclusion criteria

We included FOVs in our analysis that satisfied the following criteria: (1) FOVs needed to contain at least 100 ROIs (that is, neurons) that fulfilled the quality criteria described above, and (2) FOVs needed to contain more than ten significantly sound-responsive neurons during baseline days.

#### Selection of sound-responsive, non-sound-responsive and inhibitory neurons for microablation

During the two baseline days before microablation (days 3 and 5), we identified sound-responsive neurons, which showed significant sound-evoked responses for at least one stimulus in each FOV. For each sound-responsive neuron, we calculated the average amplitude across all the stimulus conditions that elicited significant responses. We derived the amplitude distribution of responsive neurons sorted in descending order for each day (days 3 and 5). We then created a single distribution after merging and sorting the two days’ distributions based on their amplitudes. In case the identity of a neuron was duplicated because the neuron was responsive on both days, the higher-rank neuron was selected to have unique neuron identities across the merged distribution. Thus, we prepared the sorted sound-responsive distribution merged for days 3 and 5 in each FOV or ‘the distribution of candidate responsive neurons’. On the day of microablation, we targeted neurons from the top of the distribution of candidate responsive neurons.

We identified non-sound-responsive neurons for days 3 and 5, respectively, which did not show a significant response to any of the sounds in each FOV, and derived the amplitude distributions of these neurons sorted by absolute amplitude in ascending order. We selected the neurons responsive on neither of the days, which was equal to the neurons existing in both distributions. We calculated the average rankings of the neurons from both the distributions and again sorted the neurons based on the average ranking in ascending order to prepare ‘the distribution of candidate nonresponsive neurons’. On the day of microablation, we targeted neurons from the highest rank (that is, smallest absolute amplitude) of candidate unresponsive neurons.

For the inhibitory neuron ablation cohort, we included sound-responsive inhibitory interneurons based on the same criteria for the sound-responsive ablation. To achieve comparable numbers of neurons to be microablated in the other cohorts in the ablation experiment, we also included interneurons with nonsignificant but large transient amplitudes after sound presentation (apparent sound-responsive neurons), in case the number of sound-responsive neurons did not reach the prerequisite number for each FOV. These apparent sound-responsive neurons were also sorted based on their amplitude averaged across days 3 and 5. We then selected neurons from the highest amplitudes. Practically, in the microablation experiment in sound-responsive, nonresponsive or inhibitory responsive neurons, we had to avoid neurons for microablation when the neurons showed unclear nuclear fluorescence due to optical occlusion by blood vessels, weak expression or out-of-focus position. Ultimately, we were able to target four to eight neurons for microablation in each FOV. Because the targeted neurons in these three experimental cohorts were determined based on either sound responsiveness and/or cell type, the spatial distribution of these neurons was generally random in each FOV.

In the control cohort, on the day when the animals would have undergone microablation in ablation experiments, mice (*n* = 7) were set in the microscope chamber under comparable levels of isoflurane anesthesia (~3 h) without any additional laser application except for monitoring calcium and nuclear fluorescence for ~30–50 min (sham procedure). Two other mice were set in the microscope chamber but only for short anesthetic exposure (from 30 min to 1 h) to acquire a few *z*-stack images of the FOVs. Our offline analysis confirmed that the control mice exposed to long isoflurane anesthesia on day 6 and the other control mice with short isoflurane exposure on day 6 did not show any distinct difference in average best response amplitude across neurons on day 7 (baseline-normalized amplitude for long anesthetic exposure: 1.072 ± 0.086; for short anesthetic exposure: 1.063 ± 0.047; two-sided *t*-test, *P* = 0.96) nor fraction of responsiveness on day 7 (baseline-normalized fraction for long anesthetic exposure: 0.915 ± 0.057; short anesthetic exposure: 1.114 ± 0.088; two-sided *t*-test, *P* = 0.085).

#### Exclusion of neurons with unsuccessful ablation and neurons nearby ablated neurons

Targeted neurons include all the neurons to which we applied the laser for ablation. For any microablation experiment, a small proportion of the targeted neurons showed an unclear effect of ablation, based on the criteria we described above. Because some unclearly ablated neurons also demonstrated abnormal calcium signals, such as lower frequency of calcium transients than before or seizure-like bursting behavior, we excluded all the targeted neurons and nearby neurons from further analysis. Following a previous study^21^, we excluded targeted neurons (regardless of whether microablation was successful or not), as well as neurons within a sphere centered on ablated neurons having a radius of 15 μm from any analysis.

#### Definition of high- and low-category neurons

In addition, to make a fair comparison between different ablation experiments, we digitally filtered out highly sound-responsive neurons and/or non-sound-responsive neurons from spared populations as a ‘filtered condition’ for further analysis. To mimic response properties and the number of ablated nonresponsive neurons in the nonresponsive ablation cohort, we defined five nonresponsive neurons with minimum absolute amplitudes per FOV in the sound-responsive ablation cohort, which we would have ablated in the nonresponsive ablation experiment (we call these nonresponsive neurons, which were supposed to be physically or digitally excluded, ‘low-category neurons’). Conversely, to mimic response properties and numbers of ablated sound-responsive neurons in the sound-responsive ablation cohort, we defined five responsive neurons with the highest amplitudes per FOV in the nonresponsive ablation cohort (we call these responsive neurons ‘high-category neurons’). We then digitally excluded low-category neurons from the spared population in the sound-responsive ablation cohort, high-category neurons in the nonresponsive ablation cohort and both low- and high-category neurons in the control cohort, respectively. For the inhibitory neuron ablation cohort, high-category neurons were defined as five responsive neurons with the highest amplitudes excluding the microablated sound-responsive inhibitory neurons in each FOV. We verified that the numbers of ablated sound-responsive neurons and other high-category neurons were comparable between cohorts (Extended Data Fig. 4e) and that the number of ablated nonresponsive neurons and other low-category neurons were also comparable (Extended Data Fig. 4f). We also confirmed that the best response amplitudes of high-category neurons showed no significant difference between cohorts (Extended Data Fig. 4h), the response amplitude of low-category neurons was not significantly different (Extended Data Fig. 4i), and the numbers of spared neurons in the filtered condition were comparable between the different ablation cohorts (Extended Data Fig. 4j).

#### Single-neuron tuning correlation and response reliability

For a given sound-responsive neuron, the trials were pseudorandomly split into two sets each containing half of the trials. Response amplitudes along the 34 stimuli, that is, tuning curve, were averaged across each half of the trials. Pearson correlation coefficients of the averaged responses were calculated between the first and second half set of trials. This procedure was repeated ten times, and we averaged these correlation values as single-neuron tuning correlations for each day. To evaluate the correlations to day 5, instead of calculating Pearson correlation coefficients of the averaged responses of half trials on the same day, we calculated correlations between the averaged response of half trials on day 5 and the averaged response of half trials on the other day. We used the same measure for all individual neurons to quantify the reproducibility of stimulus responses between different subsets of trials as single-neuron response reliability across trials^70,71^ (Figs. 3a, 5c and 6e).

#### Estimation of representational maps

For a given day and FOV, Pearson correlation coefficients of single-trial sound-evoked response vectors were calculated for all pairwise combinations of response vectors from the same sound and across pairs of sounds^5^. A similarity matrix was then constructed where each element in the matrix corresponded to averaged correlation coefficients for each combination of stimuli, excluding pairs with the same trials. Note that for each diagonal element, trial-averaged correlation to the same stimulus corresponds to the trial-to-trial reliability of population responses to each stimulus. The representational map was generated by averaging all the similarity matrices across all the FOVs in each cohort of mice for each day. A dimension-reduced representational map was also formed by applying classical multidimensional scaling (MDS), where a similarity matrix was mapped onto two-dimensional (first and second) MDS space. Each data point in the space represents the relative distance of the correlation profile in the similarity matrix corresponding to 1 of the 34 standard stimuli.

#### Decoding

A support vector machine with a linear kernel was trained to discriminate each pair of stimuli from the 34 stimuli as a binary classifier using a built-in function in MATLAB (fitcecoc). Whether training and testing were done on the same day or done on different days, cross-validation was performed by fivefold cross-validation. Training and testing the classifier were conducted for a population in each FOV. Decoding performance was defined as the percentage of correctly classified trials, which was averaged across FOVs per mouse and averaged across mice for each experimental cohort.

#### Categorization of neurons based on best response amplitude

Because various single-neuron response properties are considered to be dependent on the response amplitude or the best response amplitude for each neuron, we categorized sound-responsive neurons into four categories according to their best response amplitudes on a given day (0–15%, 15–25%, 25–55% and 55–1,000% in Δ*F*/*F*, respectively). Because the best response amplitude is largely log-normally distributed, the largest best amplitude category covers the extensive range in Δ*F*/*F* so that, in each category, roughly the same number of neurons are included for each experimental cohort (mean ± s.d., 25 ± 6.5%, 25 ± 7.4% and 25 ± 6.7% of neurons for each bin in the sound-responsive cohort, non-sound-responsive cohort and control cohort, respectively).

#### Quantification of tuning width

For a given responsive neuron, the trial-averaged response amplitude across the 34 stimuli, that is, tuning curve, was sorted by descending order (from the largest amplitude for the 1st stimulus to the smallest amplitude for the 34th stimulus) and normalized by the largest amplitude. After categorizing individual neurons into bins according to their best response amplitudes described above, the sorted and normalized tuning curves were averaged for each bin and for each day (Fig. 3e). The tuning width was estimated as the average normalized amplitude at the 15th stimulus on a given day (Fig. 3f).

#### Simulation for the effect of tuning width on the similarity matrix

We simulated how the change in tuning width could affect the similarity matrix to prove to what extent the disturbance of the similarity matrix on day 7 could be derived from the reduction of tuning width. The tuning curves of neurons in each best amplitude bin during baseline days were scaled by multiplying experimentally observed normalized amplitudes across sorted stimuli for each bin of the best amplitude. We again constructed the similarity matrix from neurons with artificially scaled tuning curves and compared the nondiagonal correlation between the simulated similarity matrix and the experimentally observed similarity matrix on day 7.

#### Signal correlation in the local population

To quantify similarity in tuning between neurons, we calculated signal correlation, that is, Pearson correlation of the trial-averaged tuning curves, between responsive neurons for each FOV on a given day. The distribution of signal correlation was again categorized into the best response amplitude bins described above according to the average best amplitude of a given neuron pair. For each best amplitude bin, we quantified the fraction of neuron pairs with high signal correlation beyond 0.7 out of all responsive neuron pairs on a given day.

#### Signal correlation between spared neurons and high-category neurons

Local population responses are largely dominated by a subset of responsive neurons with a high level of responsiveness. To investigate the impact of microablation on the tuning dynamics of spared neurons with respect to those high-category neurons serving as a reference of the preablation local population response, we calculated pairwise signal correlations between the tuning of high-category neurons and the spared neurons responsive on a given day. In the sound-responsive ablation cohort, high-category neurons were the actual target of microablation, whereas in the other cohorts, nonresponsive, inhibitory or no neurons were ablated. After the distribution of signal correlations was categorized into bins according to the correlation values (from –0.6 to 1.0 with a 0.2 bin size), we split the signal correlation distribution based on the responsiveness of spared neurons on the day before microablation: neurons responsive on day 5 and neurons unresponsive on day 5 but responsive on another day. We then calculated the fraction of each day 5 responsive or nonresponsive neurons, respectively, in each signal correlation bin. To characterize the microablation-induced change of the signal correlation after ablation, we normalized the fraction of neurons responsive on day 5 or neurons unresponsive on day 5 by subtracting the baseline fraction of these sets of neurons, respectively, which were averaged across days 1 and 3 for each signal correlation bin.

#### Balance of general excitation and inhibition levels in the local network

To investigate the balance of network-level activities between excitatory and inhibitory neurons, we calculated the product of the fraction of responsive neurons and the average best response amplitude in each mouse (Extended Data Fig. 8e) as a general estimate of the total amount of excitatory response and inhibitory response, that is, ‘total responses’ in the local network. The balance was quantified as the ratio of (total response in excitatory neurons) / (total response in excitatory neurons + total response in inhibitory neurons).

### Statistics and reproducibility

Data collection and analyses were not performed blind to the conditions of the experiments. No statistical tests were applied to determine sample sizes, but our sample sizes are similar to those reported in previous publications^21^. Data were excluded based on several criteria described above, such as ROI inclusion, FOV inclusion and the microablation protocol. Preliminary image analyses were performed using ImageJ/Fiji (ImageJ 1.53t, Java 1.8.0_172, 64 bit). All statistical analyses were performed using MATLAB (versions R2016b and R2022a, MathWorks). In all cases, except for Extended Data Fig. 7c–f, we treated not neurons but mice as independent observations. Sample sizes were largely comparable between conditions (number of mice between the different cohorts). Most statistical comparisons were performed using a two-sided *t*-test, comparing means within individual mice for two different conditions, such as baseline days and a postablation day, after FDR correction for multiple comparisons to assess changes following microablation in each experimental cohort. For comparisons between different experimental cohorts on a given day or over a set of days, a two-sided *t*-test was applied after FDR correction. When classifying individual neurons for sound-evoked responsiveness, because poststimulus activities across trials were highly non-normally distributed, a signed-rank test was performed after Benjamini–Hochberg correction for multiple comparisons against the number of stimuli. For the same reason, to compare the normalized correlation coefficient in the representational maps across the experimental cohorts (Extended Data Figs. 5 and 9), we used nonparametric pairwise multiple comparisons, Dunn’s test for the different pairs of cumulative distributions of the normalized correlation coefficients and a Mann–Whitney *U*-test after FDR correction to compare medians between the cohorts for each tested day. For comparisons across the experimental cohorts in the other analyses from Figs. 3–8, in general, we performed permutation tests on a given day after microablation by shuffling samples 200 times across the three cohorts and estimating *P* values for the experimentally observed data for each cohort. Regarding the change in similarity matrix constructed after shuffling neurons across FOVs (Fig. 3g), first, we applied a permutation test on a given day by shuffling neurons across the three experimental cohorts, randomly selecting the same number of neurons as that in the cohort to be compared and constructing the similarity matrix from the selected neurons. We repeated the procedure 500 times and estimated the distribution of the nondiagonal correlation from the cohort-permuted neurons by the 95% confidence interval (2.5% and 97.5% percentiles of the data as the bottom and top bounds, respectively). We then compared the nondiagonal correlation of the similarity matrices from population vectors shuffled across FOVs in each experimental cohort to the two-sided confidence interval from the corresponding cohort. Second, we further tested the change in normalized correlation in the sound-responsive cohort (or non-sound-responsive cohort) from the control cohort after ablation. We applied the same permutation procedure but with only two experimental cohorts for comparison (sound-responsive cohort and control or non-sound-responsive cohort and control). For the validation experiment of interneuron labeling (Extended Data Fig. 8b), multiple slices or cells measured from the same animal were treated as technical replicates.

### Reporting summary

Further information on research design is available in the Nature Portfolio Reporting Summary linked to this article.

## Online content

Any methods, additional references, Nature Portfolio reporting summaries, source data, extended data, supplementary information, acknowledgements, peer review information; details of author contributions and competing interests; and statements of data and code availability are available at 10.1038/s41593-025-01982-7.

## Supplementary information

Supplementary Information Supplementary Tables 1–3.

Reporting Summary

## Source data

Source Data Fig. 1 Similarity matrices over the course of imaging days in the control cohort (including high-category and low-category neurons) in Fig. 1h.

Source Data Fig. 2 Similarity matrices over the course of imaging days in the sound-responsive ablation cohort in Fig. 2d and in the non-sound-responsive ablation cohort in Fig. 2f (both excluding targeted neurons and high- or low-category neurons).

Source Data Fig. 7 Similarity matrices over the course of imaging days in the inhibitory neuron ablation cohort in Fig. 7c (excluding targeted neurons and high- and low-category neurons).

## Data Availability

Data will be made available upon reasonable request by the corresponding author. In addition, preprocessed calcium activity data extracted from raw imaging data, corresponding ROI coordinates and sound stimulation data have been deposited at G-Node (www.g-node.org) and are publicly available (10.12751/g-node.2gxcrx, ‘data’ directory). Source data are provided with this paper.

## Code availability

The primary analysis pipeline has also been deposited at G-Node and can be accessed there (10.12751/g-node.2gxcrx, ‘tutorials’ directory).
