# Supplementary material for: Homeostasis of a representational map in the neocortex
Source: Nat Neurosci. 2025 Jun 5;28(7):1533–45. doi: 10.1038/s41593-025-01982-7 (PMC12229895; doi:10.1038/s41593-025-01982-7)
Supplement: Supplementary file 1 — Supplementary Tables 1–3. [file 41593_2025_1982_MOESM1_ESM.pdf]

nature neuroscience

Supplementary information https://doi.org/10.1038/s41593-025-01982-7

Homeostasis of a representational map in the neocortex

In the format provided by the authors and unedited

Supplementary Table 1.

Detailed statistical results used in main Figures. In general, one-way ANOVA was applied to test change of parameter of interest over time points, two-sided t-test with False Discovery Rate correction was applied to test difference of a parameter of interest between baseline time points and a post-ablation day. Comparison among the three groups (sound responsive ablation, non-sound responsive ablation, and control) was done by permutation test.

**1) Figure panel**	**2) Data type, group**	**3) Test type**	**4) Statistical info., Degree of freedom, P values**
Fig. 1e	Signal correlation to day 5 in control cohort.	One-way ANOVA over time points.	$F(6, 56) = 7.77$, $p = 4.21\times10^{-6}$
		Two-sided paired *t*-test between day 3 and day 7, between day 1 and day 9.	day 3 vs day 7: $p = 0.69$, day 1 vs day 9: $p = 0.12$
Fig. 1g	Signal correlation for each day in control cohort.	One-way ANOVA over time points.	$F(6, 56) = 0.55$, $p = 0.77$
Fig. 1i	Normalized correlations of population response vectors in control cohort.	Two-sided *t*-test between baseline days vs. days after ablation with False Discovery Rate (FDR) correction.	Diagonal elements: $p = 0.83$, $p > 0.9$, $p = 0.059$, $p = 0.059$; Non-diagonal elements: $p = 0.84$, $p = 0.84$, $p = 0.029$, $p = 0.021$, for day 7, 9, 11, 15, respectively.
Fig. 2e	Normalized correlations of population response vectors in sound responsive ablation cohort.	Two-sided *t*-test of normalized average correlation between baseline days vs. days after ablation with FDR correction.	Diagonal elements: $p = 0.044$, $p = 0.96$, $p = 0.96$, $p = 0.96$ for day 7, 9, 11, 15, respectively. Non-diagonal elements: $p = 2.06\times10^{-5}$, $p = 0.24$, $p = 0.24$, $p = 0.014$ for day 7, 9, 11, 15, respectively.
		One-way ANOVA of normalized average correlation across days.	Diagonal elements: $F(6, 63) = 0.91$, $p = 0.49$ Non-diagonal elements: $F(6, 63) = 2.25$, $p = 0.049$.
Fig. 2g	Normalized correlations of population response vectors in non-sound responsive ablation cohort.	Two-sided *t*-test of normalized average correlation between baseline days vs. days after ablation.	Diagonal elements: $p > 0.72$ for all post-ablation days. Non-diagonal elements: $p > 0.83$ for all post-ablation days.
		One-way ANOVA of normalized average correlation across days.	Diagonal elements: $F(6, 63) = 0.68$, $p = 0.67$; Non-diagonal elements: $F(6, 63) = 0.58$, $p = 0.75$
Fig. 3a	Normalized response reliability over time points.	Two-sided *t*-test of normalized reliability between baseline days vs. days after ablation with FDR corrected *p*-values.	Sound responsive cohort: $p = 0.034$ on day 7, $p > 0.6$ for the other post-ablation days (day 9, 11, 15); Non-sound responsive cohort: $p = 0.50$, $p = 0.276$, $p = 0.28$, $p = 0.53$ for day 7, 9, 11, 15, respectively; Control: $p > 0.6$ for all post-ablation days.
	Normalized response reliability across three cohorts.	Permutation test across groups on day 7.	Sound responsive cohort: $p = 0.045$; Non-sound responsive cohort and Control: $p > 0.62$.
Fig. 3b	Normalized correlations from trial-averaged	Two-sided *t*-test of normalized correlation coefficient between baseline days vs. days	Sound responsive cohort: $p = 7.90\times10^{-4}$, $p = 0.081$, $p > 0.60$, $p = 0.040$ for day 7, 9, 11, 15, respectively;

	population response vectors.	after ablation (asterisks on top).	Non-sound responsive cohort: $p > 0.60$ for all post-ablation days;<br>Control: $p > 0.60$, $p = 0.48$, $p = 0.051$, $p = 0.051$ for day 7, 9, 11, 15, respectively.
		Permutation test across groups.	Sound responsive cohort: $p < 0.005$, $p = 0.15$, $p = 0.71$, $p = 0.305$ for day 7, 9, 11, 15;<br>Non-sound responsive cohort: $p > 0.78$ for all post-ablation days;<br>Control: $p = 0.95$, $p = 0.37$, $p = 0.10$, $p = 0.065$ for day 7, 9, 11, 15, respectively.
Fig. 3c	Fraction of sound-responsive neurons over the imaging days.	One-way ANOVA across days.	Sound responsive cohort: $F(6, 63) = 1.47$, $p = 0.20$;<br>Non-sound responsive cohort: $F(6, 63) = 0.80$, $p = 0.57$;<br>Control: $F(6, 56) = 0.26$, $p = 0.95$.
Fig. 3d	Normalized best response amplitude of neurons responsive for each day.	Two-sided *t*-test between baseline days and days after ablation with FDR correction.	Sound responsive cohort: $p = 0.98$, $p = 0.0045$, $p = 1.77\times10^{-5}$, $p = 3.71\times10^{-7}$ for day 7, 9, 11, 15;<br>Non-sound responsive cohort: $p = 0.835$, $p = 0.595$, $p = 0.595$, $p = 0.0191$ for day 7, 9, 11, 15, respectively;<br>Control: $p > 0.82$ for all post-ablation days.
		Permutation test across groups.	Sound responsive cohort: $p = 0.57$, $p = 0.11$, $p = 0.005$, $p = 0.005$ for day 7, 9, 11, 15;<br>Non-sound responsive cohort: $p = 0.49$, $p = 0.70$, $p = 0.925$, $p = 0.315$ for day 7, 9, 11, 15;<br>Control: $p = 0.475$, $p = 0.80$, $p = 0.82$, $p = 0.995$ for day 7, 9, 11, 15, respectively.
Fig. 3f	Change of normalized response amplitudes from baseline at 15th largest stimulus index.	Two-sided *t*-test between baseline days and days after ablation with FDR correction at 15th stimulus index.	Sound responsive cohort: day 7, $p = 0.0019$; day 9, $p = 0.049$; day 11, $p = 0.026$; day 15, $p = 5.66\times10^{-5}$;<br>Non-sound responsive cohort: $p > 0.89$ for day 7, 9, 11 respectively and $p = 0.033$ for day 15;<br>Control cohort: $p > 0.9$ for all post-ablation days.
		Permutation test across groups.	Sound responsive cohort: $p = 0.020$, $p = 0.11$, $p = 0.025$, $p = 0.005$ for day 7, 9, 11, 15, respectively;<br>Non-sound responsive cohort: $p > 0.39$ for all post ablation days;<br>Control: $p > 0.63$ for all post-ablation days.
Fig. 3g	Normalized non-diagonal correlations from population response vectors shuffled across FOVs.	Comprison between normalized correlation (r_n) of neuron-shuffled populations and the lower boundary (CI_L) of the shaded area.	Sound responsive cohort: day 7: $r_n = 0.62$, $CI_L = 0.71$; day 9: $r_n = 0.59$, $CI_L = 0.66$; day 11: $r_n = 0.69$, $CI_L = 0.70$; day 15: $r_n = 0.63$, $CI_L = 0.66$.
		Permutation test across groups by shuffling neurons.	Sound responsive cohort vs. Control cohort: $p < 0.006$ for all post-ablation days;<br>Non-sound responsive cohort vs. control cohort: $p = 0.010$, $p = 0.86$, $p = 0.19$, $p = 0.74$ for day 7, 9, 11, 15, respectively.
Fig. 3h	Color map displaying the change in the fraction of pairs	Two-way ANOVA across days and across amplitude bins.	Sound responsive cohort (left) : $F(6, 270) = 3.43$, $p = 0.0028$ across days, $F(3, 270) = 8.33$, $p = 2.55\times10^{-5}$ across amplitude bins (Average

	among responsive neurons showing a high signal correlation.		number of cell pairs during baseline days for each best response bin: 0-0.15 ΔF/F bin of best amplitude: 3.1±1.1; 0.15-0.25 bin: 28.6±10.2; 0.25-0.55 bin: 68.1±15.2; 0.55-10 bin: 54.2±15.5).<br><br>Non-sound responsive ablation (middle): F(6, 269) = 1.26, *p* = 0.27 across days, F(3, 269) = 0.894, *p* = 0.45 across amplitude bins (Average number of cell pairs: 0-0.15 bin: 4.8±1.0; 0.15-0.25 bin: 43.5±12.3; 0.25-0.55 bin: 105.9±24.9; 0.55-10 bin: 68.6±20.6).<br><br>Control (right): F(6, 241) = 1.91, *p* = 0.080 across days, F(3, 241) = 2.01, *p* = 0.11 across amplitude bins (Average number of cell pairs: 0-0.15 bin: 2.3±0.8; 0.15-0.25 bin: 13.5±4.4; 0.25-0.55 bin: 32.8±9.1; 0.55-10 bin: 33.4±9.9).
Fig. 3i	Change in fraction of neuron pairs with high signal correlation from baseline at the largest best response category.	Two-sided *t*-test between baseline days vs. days after ablation with FDR correction.	Sound responsive cohort: *p* = 0.29, *p* = 0.017, *p* = 0.0017, p = 1.05×10^{-4} for day 7, 9, 11, 15;<br>Non-sound responsive cohort: *p* = 0.062, *p* = 0.062, *p* = 0.42, p = 0.062 for day 7, 9, 11, 15, respectively;<br>Control: *p* > 0.14 for all post-ablation days;
		Permutation test across groups.	Sound responsive cohort: *p* = 0.65, *p* = 0.23, *p* = 0.035, *p* = 0.005 for day 7, 9, 11, 15, respectively;<br>Non-sound responsive cohort: *p* > 0.20 for all post-ablation days;<br>Control: *p* > 0.58 for all post-ablation days.
Fig. 4a	Response stability as the fraction of neurons categorized as sound responsive on two consecutive imaging days.	One-way ANOVA across days.	Sound responsive cohort: F(5, 54) = 6.03, *p* = 0.0002;<br>Non-responsive cohort, F(5, 54) = 0.39, *p* = 0.85;<br>Control cohort, F(5, 48) = 0.50, *p* = 0.78.
		Post-hoc two-sided *t*-test between change during baseline days vs. change from day 5 to day 7 in sound responsive cohort.	*p* = 9.47×10^{-5}
		Two-sided *t*-test with FDR correction on day 5 → 7.	Sound responsive cohort vs. control, *p* = 0.064;<br>Sound responsive cohort vs the non-responsive cohort, *p* = 0.017.
Fig. 4b	Average best response amplitude normalized to day 5 (left) in the three experimental cohorts.	Left; two-sided *t*-test between day 3 vs. day 7.	Sound responsive cohort: *p* = 4.54×10^{-4};<br>Non-sound responsive cohort, *p* = 0.33;<br>Control, *p* = 0.63.
		Group comparison by one-sided t-test with FRA correction on day 7.	Sound responsive cohort vs. control, *p* = 0.039;<br>Sound responsive cohort vs. Non-sound responsive cohort, *p* = 0.096.
Fig. 4b	Average best response amplitude normalized to days 1 and 3 (right) in the	Right: One-way ANOVA across days.	Sound responsive cohort: F(5, 54) = 6.19, *p* = 0.0001;<br>Non-sound responsive cohort: F(5, 54) = 2.14, *p* = 0.075;<br>Control: F(5, 48) = 0.45, *p* = 0.81;

	three experimental cohorts.	Two-sided *t*-test with FDR correction for group comparison.	Sound responsive cohort vs. control: $p = 0.74$, $p = 0.14$, $p = 0.017$, $p = 0.0096$ for day 7, 9, 11, 15, respectively;<br>Sound responsive cohort vs. Non-sound responsive cohort: $p = 0.74$, $p = 0.14$, $p = 0.017$, $p = 0.23$ for day 7, 9, 11, 15;<br>Non-sound responsive cohort vs. Control: $p = 0.74$, $p = 0.88$, $p = 0.82$, $p = 0.068$ for day 7, 9, 11, 15.
Fig. 4c	Change of tuning width for neurons responsive on day 5 (left).	Two-sided *t*-test between baseline days vs. days after ablation.	Sound responsive cohort: day 7: $p = 0.011$; day 9: $p = 0.25$; day 11: $p = 0.011$; day 15: $p = 0.0010$.<br>Non-sound responsive cohort: $p > 0.48$ for all post-ablation days;<br>Control: $p > 0.08$ for all post-ablation days.
		Permutation test of sound responsive ablation across groups.	$p = 0.040$, $p = 0.25$, $p < 0.005$, $p = 0.015$ for day 7, 9, 11, 15, respectively.
Fig. 4c	Change of tuning width for neurons not responsive on day 5 but responsive on another day (Right).	Two-sided *t*-test between baseline days vs. days after ablation.	Sound responsive cohort: $p = 0.14$, $p = 0.57$, $p = 0.57$, $p = 0.034$ for day 7, 9, 11, 15, respectively.<br>Non-sound responsive cohort: $p > 0.52$ for all post-ablation days.<br>Control: $p > 0.51$ for all post-ablation days.
		Permutation test of sound responsive ablation across groups.	$p = 0.17$, $p = 0.39$, $p = 0.41$, $p = 0.065$ for day 7, 9, 11, 15, respectively.
Fig. 4d	Change in fraction of neuron pairs with high signal correlation, categorized as responsive on day 5 for sound responsive cohort (left), non-sound responsive cohort (middle), and control cohort (right).	Two-way ANOVA across days and across amplitude bins.<br><br>Two-sided *t*-test between baseline days and days after ablation (day 9-15).	Sound responsive cohort (left): $F(6, 270) = 4.87$, $p = 9.52\times10^{-5}$ across days; $F(3, 270) = 5.57$, $p = 0.0010$ across amplitude bins.<br>*t*-test, $p = 8.68\times10^{-6}$.<br><br>Non-sound responsive cohort (middle): $F(6, 270) = 0.43$, $p = 0.86$ across days; $F(3, 270) = 1.035$, $p = 0.38$ across amplitude bins.<br>*t*-test, $p = 0.098$.<br><br>Control (right):<br>$F(6, 242) = 2.25$, $p = 0.039$ across days; $F(3, 242) = 0.025$, $p = 0.99$ across amplitude bins;<br>*t*-test, $p = 0.41$.
Fig. 4e	Change in fraction of neuron pairs with high signal correlations for neurons of the largest best response category.	Permutation test across groups to each experimental cohort.	Sound responsive cohort: $p = 0.010$, $p = 0.035$, $p = 0.13$, $p = 0.21$ for day 7, 9, 11, 15, respectively.<br>Non-sound responsive cohort: $p > 0.71$ for all post-ablation days.<br>Control: $p > 0.41$ for all post-ablation days.<br>(Average number of cell pairs responsive on day 5 for each best response bin, sound responsive ablation cohort: 0-0.15 ΔF/F bin of best amplitude: 1.4±0.5; 0.15-0.25 bin: 14.1±6.0; 0.25-0.55 bin: 36.0±9.9; 0.55-10 bin: 27.8±9.1; non-sound responsive ablation: 0-0.15 bin: 2.7±0.8; 0.15-0.25 bin: 20.3±6.4;

			0.25-0.55 bin: 56.7±15.6; 0.55-10 bin: 41.2±13.6; control: 0-0.15 bin: 0.9±0.3; 0.15-0.25 bin: 6.7±2.3; 0.25-0.55 bin: 16.7±4.8; 0.55-10 bin: 17.3±5.1)
Fig. 4f	Colormaps showing changes in fraction of neuron pairs categorized as not responsive on day 5 but responsive on the other day for sound responsive cohort (left), non-sound responsive cohort (middle), and control cohort (right).	Two-way ANOVA across days and across amplitude bins.<br><br>Two-sided *t*-test between baseline days and days after ablation (day 9-15).	Sound responsive cohort (left): F(5, 231) = 3.02, $p = 0.012$ across days; F(3, 231) = 3.09, $p = 0.028$ across amplitude bins; *t*-test, $p = 0.034$.<br><br>Non-sound responsive cohort (middle): F(5, 229) = 1.43, $p = 0.22$ across days; F(3,229) = 2.749, $p = 0.044$ across amplitude bins; *t*-test, $p = 0.22$.<br><br>Control cohort (right): F(5, 206) = 0.58, $p = 0.71$ across days; F(3, 206) = 0.51, $p = 0.68$ across amplitude bins; *t*-test, $p = 0.79$.
Fig. 4g	Changes in fraction of neuron pairs categorized as not responsive on day 5 but responsive on the other day.	Permutation test across groups to each experimental cohort.	Sound responsive cohort: $p = 0.91$, $p = 0.21$, $p = 0.040$, $p = 0.010$ for day 7 , 9, 11, 15, respectively.<br>Non-sound responsive cohort: $p > 0.11$ for all post-ablation days.<br>Control cohort: $p > 0.41$ for all post-ablation days.<br>(Average number of cell pairs unresponsive on day 5 but responsive on another day for each best response bin, sound responsive ablation cohort: 0-0.15 bin: 0.4±0.2; 0.15-0.25 bin: 4.0±1.0; 0.25-0.55 bin: 8.9±2.3; 0.55-10 bin: 13.4±7.0; non-sound responsive ablation: 0-0.15 bin: 0.5±0.2; 0.15-0.25 bin: 6.1±2.1; 0.25-0.55 bin: 14.2±4.1; 0.55-10 bin: 9.7±3.4; control: 0-0.15 bin: 0.4±0.1; 0.15-0.25 bin: 2.4±0.8; 0.25-0.55 bin: 4.3±1.1; 0.55-10 bin: 7.3±2.1)
Fig. 4h	Colormaps showing changes in fraction of neuron pairs of neurons in which one neuron was categorized responsive on day 5 and the other categorized not responsive on day 5 but responsive on another day.	Two-way ANOVA across days and across amplitude bins.	Sound responsive cohort (left): F(5, 217) = 0.33, $p = 0.89$ across days; F(3, 217) = 2.85, $p = 0.038$ across amplitude bins.<br><br>Non-sound responsive cohort (middle): F(5, 221) = 0.25, $p = 0.94$ across days; F(3, 221) = 0.070, $p = 0.98$ across amplitude bins.<br><br>.Control cohort (right): F(5, 195) = 0.42, $p = 0.83$ across days; F(3, 195) = 2.53, $p = 0.059$ across amplitude bins.
Fig. 4i	Changes in fraction of neuron pairs of neurons in which one neuron was categorized responsive on day 5 and the other categorized not	Permutation test across groups for all post-ablation days.	Sound responsive cohort : $p > 0.18$;<br>Non-sound responsive cohort: $p > 0.11$;<br>Control : $p > 0.43$.<br>(Average number of cell pairs responsive on day 5 vs. unresponsive on day 5 for each best response bin, sound responsive ablation cohort: 0-0.15 bin: 0.8±0.3; 0.15-0.25 bin: 9.3±3.4; 0.25-0.55 bin: 21.2±5.6; 0.55-10 bin:

	responsive on day 5 but responsive on another day.		16.8±4.8; non-sound responsive ablation: 0-0.15 bin: 1.0±0.3; 0.15-0.25 bin: 14.6±4.8; 0.25-0.55 bin: 33.8±8.4; 0.55-10 bin: 21.9±6.5; control: 0-0.15 bin: 0.7±0.3; 0.15-0.25 bin: 3.8±1.2; 0.25-0.55 bin: 10.3±3.1; 0.55-10 bin: 11.2±3.5)
Fig. 5d	Normalized change in fraction of spared neurons with high signal correlation (>0.6) between spared neurons responsive on day 5 and high category neurons.	One-way ANOVA over days.	Sound responsive ablation $F(5, 114) = 3.58$, $p = 0.0048$;<br>Non-responsive ablation: $F(5, 114) = 3.7$, $p = 0.0039$;<br>Control: $F(5, 101) = 1.44$, $p = 0.21$.
		Comparison across groups by two-sided *t*-test with FDR correction (two-colored asterisks on top)	Sound responsive ablation vs. control on day 7, $p = 0.042$; day 9, $p = 0.0051$; day 11, $p = 0.049$; day 15, $p = 0.061$;<br>Sound responsive ablation vs. non-sound responsive ablation on day 7, $p = 0.15$; day 9, $p = 0.057$; day 11, $p = 0.18$; day 15, $p = 0.85$;<br>Non-responsive ablation vs. control on day 7, $p = 0.16$; day 9, $p = 0.047$; day 11, $p = 0.18$; day 15, $p = 0.061$.
	Normalized change in fraction of spared neurons with high signal correlation (>0.6) between spared neurons non-responsive on day 5 but gaining responsiveness on a given day and high category neurons.	Comparison across groups by one-sided *t*-test with FDR correction.	Sound responsive ablation vs. Control on day 7, $p = 0.21$; day 9, $p = 0.05$; day 11, $p = 0.049$; day 15, $p = 0.43$;<br>Sound responsive ablation vs. Non-responsive ablation on day 7, $p = 0.21$; day 9, $p = 0.29$; day 11, $p = 0.054$; day 15, $p = 0.47$;<br>Non-responsive ablation vs. Control on day 7, $p = 0.15$; day 9, $p = 0.086$; day 11, $p = 0.35$; day 15, $p = 0.43$.<br>(two-colored asterisks on top)
		One-sided *t*-test with FDR correction between early baseline days (day 1 and 3) and late post ablation days (day 9, 11, 15) for each experimental cohort.	Sound responsive cohort: $p = 0.043$;<br>Non-sound responsive cohort: $p = 0.21$;<br>Control cohort: $p = 0.41$.<br>(asterisk at bottom)
Fig. 6c	Normalized response reliability across trials of single neurons averaged for all excitatory (left) on each given day.	Two-sided *t*-test of normalized reliability between baseline days vs. days after ablation with FDR correction. sound responsive cohort.	Sound responsive cohort (orange): $p = 0.041$, $p = 0.041$, $p = 0.33$, $p = 0.14$ for day 7, 9, 11, 15, respectively;<br>Non-sound responsive cohort (blue): $p > 0.60$ for all post-ablation days;<br>Control (green): $p > 0.14$ for all post-ablation days.
		Permutation test across groups (one-sided *p*-values).	Sound responsive cohort (orange): $p = 0.048$, $p = 0.035$, $p = 0.26$, $p = 0.27$ for day 7, 9, 11, 15, respectively;<br>Non-sound responsive cohort (blue): $p > 0.13$ for all post-ablation days;<br>Control (green): $p > 0.16$ for all post-ablation days.<br>(Average number of responsive excitatory neurons during baseline days for sound responsive cohort: 103.7±19.1 (mean ± s.e.m. across mice); non-sound responsive cohort:

			105.8±11.4; control cohort: 117.7±26.7, respectively)
Fig. 6c	Normalized response reliability across trials of single neurons averaged for all inhibitory (right) neurons on each given day.	Two-sided *t*-test of normalized reliability between baseline days vs. days after ablation with FDR correction.	Sound responsive cohort (orange): $p = 0.32$, $p = 0.033$, $p = 0.0074$, $p = 0.049$ for day 7, 9, 11, 15, respectively;<br>Non-sound responsive cohort (blue): $p > 0.66$ for all post-ablation days;<br>Control (green): $p > 0.36$ for all post-ablation days.
		One-sided *p*-values from permutation test across groups.	Sound responsive cohort: $p = 0.15$, $p = 0.013$, $p = 0.0025$, $p = 0.12$ for day 7, 9, 11, 15, respectively;<br>Non-sound responsive cohort: $p > 0.32$ for all post-ablation days;<br>Control cohort: $p > 0.27$ for all post-ablation days.<br>(Average number of responsive inhibitory neurons during baseline days for sound responsive cohort: 6.8±2.1 (mean ± s.e.m. across mice); non-sound responsive cohort: 7.3±2.1; control cohort: 8.0±1.9, respectively.)
Fig. 6d	Fraction of sound responsive neurons for excitatory (Left) and inhibitory (Right) neurons.	Two-sided *t*-test of fraction between baseline days vs. days after ablation with FDR correction.	Excitatory neurons<br>Sound responsive cohort for all post-ablation days: $p > 0.30$;<br>Non-sound responsive cohort for all post-ablation days: $p > 0.90$;<br>Control cohort: $p > 0.61$.<br><br>Inhibitory neurons<br>Sound responsive cohort: $p = 0.022$ on day 11, but $p > 0.47$ for the other post-ablation days;<br>Non-sound responsive cohort: $p > 0.97$;<br>Control: $p > 0.57$ for all post-ablation days.
Fig. 6e	Normalized best response amplitude of excitatory (left) neurons responsive for each day.	One-sided *t*-test of best response between baseline days vs. days after ablation in excitatory neurons with FDR correction.	Sound responsive cohort: $p = 0.45$, $p = 0.045$, $p = 0.0017$, $p = 0.0003$ for day 7, 9, 11, 15, respectively;<br>Non-sound responsive cohort:<br>$p = 0.38$, $p = 0.076$, $p = 0.15$, $p = 0.076$ for day 7, 9, 11, 15, respectively;<br>Control cohort: $p > 0.48$ for all post-ablation days; inhibitory neurons: $p > 0.25$ for all post-ablation days.
		Permutation test of each ablation cohort to control cohort for all post-ablation days.	Sound responsive ablation cohort: $p > 0.05$, for day 7, 9, 11, $p < 0.025$ for day 15, respectively;<br>Non-sound responsive ablation cohort: $p > 0.05$, for all post-ablation days.
Fig. 6e	Normalized best response amplitude of inhibitory (right) neurons responsive for each day.	One-sided *t*-test between baseline days vs. days after ablation in inhibitory neurons with FDR correction.	Sound responsive cohort: $p = 0.053$, $p = 0.043$, $p = 0.078$, p = 0.17 for day 7, 9, 11, 15, respectively;<br>Non-sound responsive cohort: $p = 0.37$, $p = 0.059$, $p = 0.059$, $p = 0.44$ for day 7, 9, 11, 15, respectively.
		Permutation test of each ablation cohort to control	Sound responsive ablation cohort: $p > 0.05$, for day 7, 9, 15, $p < 0.05$ for day 11, respectively;

		cohort for all post-ablation days.	baseline normalized amplitude on day 15: 1.15 ± 0.26.<br>Non-sound responsive ablation cohort to control cohort: $p > 0.05$, for day 7, 15, $p < 0.025$ for day 9, 11, respectively.
Fig. 6f	Normalized fraction of neurons responsive on both days for excitatory (left) neurons.	Two-sided *t*-test between the overlap during baseline days (1→3, 3→5) vs. the overlap during post-ablation days (5→7, 7→9, 9→11, 11→15) for excitatory neurons.	Sound responsive ablation, $p = 0.0024$;<br>Non-sound responsive ablation, $p = 0.21$;<br>Control, $p = 0.67$;
		Two-sided *t*-test with FDR adjustment for group comparison in the overlap during post-ablation days, in excitatory neurons.	Sound responsive ablation vs. Non-sound responsive ablation: $p = 9.11 \times 10^{-5}$;<br>Sound responsive ablation vs. Control: $p = 0.003$;<br>Non-sound responsive ablation vs. Control: $p = 0.34$;
Fig. 6f	Normalized fraction of neurons responsive on both days for inhibitory (right) neurons.	Two-sided *t*-test between the overlap during baseline days vs. the overlap during post-ablation days for inhibitory neurons.	Sound responsive ablation: $p = 7.49 \times 10^{-4}$;<br>Non-sound responsive ablation: $p = 0.15$;<br>Control: $p = 0.15$.
		Two-sided *t*-test with FDR adjustment for group comparison during post-ablation days, in inhibitory neurons.	Sound responsive ablation vs. non-sound responsive ablation: $p = 1.79 \times 10^{-4}$;<br>Sound responsive ablation vs. control: $p = 3.99 \times 10^{-4}$;<br>Non-sound responsive ablation vs. control, $p = 0.76$.
Fig. 7d	Normalized correlations of population response vectors averaged across diagonal elements and non-diagonal elements in the similarity matrices.	Two-sided *t*-test of normalized average correlation between baseline days vs. days after ablation with FDR correction.	Diagonal elements: $p = 4.84 \times 10^{-4}$, $p = 0.011$, $p = 0.011$, $p = 0.043$ for day 7, 9, 11, 15, respectively;<br>Non-diagonal elements: $p = 7.87 \times 10^{-4}$, $p = 0.039$, $p = 0.0047$, $p = 0.50$ for day 7, 9, 11, 15, respectively.
		One-way ANOVA of normalized average correlation across days.	Diagonal elements: $F(6, 34) = 4.11$, $p = 0.0033$;<br>Non-diagonal elements: $F(6, 34) = 2.19$, $p = 0.068$.
Fig. 8a	Normalized response reliability across trials of single neurons averaged across all neurons on each given day.	One-way ANOVA over the imaging days.	$F(6, 34) = 2.76$, $p = 0.027$.
		Two-sided t-test between baseline days vs. day 7, 9, 11, and 15, after FDR correction.	$p = 0.016$, 0.0014, 0.0036, 0.048 for day 7, 9, 11, and 15.
		Permutation test for group comparison between inhibitory neuron cohort and control.	$p = 0.10$, $p = 0.0050$, $p = 0.020$, $p = 0.48$ for day 7, 9, 11, 15, respectively.
Fig. 8b	Normalized correlations averaged across non-diagonal elements in the similarity matrix	One-way ANOVA over the imaging days.	$F(6, 34) = 0.68$, $p = 0.66$.
		Two-sided t-test of normalized correlation coefficient between	$p > 0.26$ for all post-ablation days.

	constructed from trial-averaged population response vectors.	baseline days vs. days after ablation with FDR correction.	
		Permutation test across inhibitory neuron ablation and control cohorts.	$p = 0.21$, $p = 0.69$, $p = 0.87$, $p = 0.93$ for day 7, 9, 11, 15, respectively.
Fig. 8c	Fraction of sound-responsive neurons over the imaging days.	One-way ANOVA over the imaging days.	F(6, 34) = 0.36, $p = 0.90$.
Fig. 8d	Normalized best response amplitude of neurons responsive for each day.	One-way ANOVA across days.	F(6, 34) = 0.95, $p = 0.47$.
		Two-sided t-test between baseline days vs. days after ablation.	$p = 0.43$, $p = 0.43$, $p = 0.13$, $p = 0.43$ for day 7, 9, 11, 15, respectively.
		Permutation test across groups.	$p = 0.68$, $p = 0.38$, $p = 0.36$, $p = 0.13$ for day 7, 9, 11, 15, respectively.
Fig. 8e	Change of normalized response amplitudes from baseline at 15th largest stimulus index in the tuning curve over days.	Two-sided t-test between baseline days vs. days after ablation with FDR correction.	$p = 0.34$, $p = 0.043$, $p = 0.17$, $p = 0.34$ for day 7, 9, 11, 15, respectively.
		Permutation test for comparison between inhibitory neuron ablation and control.	$p = 0.49$, $p = 0.18$, $p = 0.085$, $p = 0.16$ for day 7, 9, 11, 15, respectively.
Fig. 8f	Change in fraction of neuron pairs with high signal correlation from baseline at the largest best response category.	Two-sided t-test between baseline days vs. days after ablation with FDR correction.	$p = 0.079$, $p = 0.017$, $p = 0.10$, $p = 0.15$ for day 7, 9, 11, 15, respectively.
		Permutation test across inhibitory neuron ablation and control.	$p = 0.37$, $p = 0.25$, $p = 0.27$, $p = 0.17$ for day 7, 9, 11, 15, respectively.
Fig. 8g	Response stability as the fraction of neurons categorized as sound responsive on two consecutive imaging days.	Two-sided *t*-test of normalized fraction of stably responsive neurons during baseline days (1→3, 3→5) vs. day 5 → 7.	$p = 0.026$
		Two-sided *t*-test between inhibitory neuron ablation cohort vs. control cohort during post-ablation days (7→9, 9→11).	$p = 0.048$
Fig. 8i	Change in fraction of spared neurons with high signal correlation (>0.6) between spared neurons responsive on day 5 and high category neurons in inhibitory neuron ablation.	Comparison between inhibitory neuron ablation cohort vs. control cohort by two-sided *t*-test with FDR correction.	day 5: $p = 0.025$; day 7: $p = 0.0061$; day 9: $p = 0.0053$; day 11: $p = 0.0028$; day 15: $p = 0.018$.
	Change in fraction of spared neurons with high signal	Comparison between inhibitory neuron ablation	day 7: $p = 0.35$; day 9: $p = 0.028$; day 11: $p = 0.072$; day 15: $p = 0.24$.

	correlation (>0.6) between spared neurons newly responsive on the other day and high category neurons in inhibitory neuron ablation.	cohort vs. control cohort by two-sided *t*-test.	
		Two-sided *t*-test between baseline days vs. days during 9 – 15.	Inhibitory neuron ablation: $p = 0.032$; Control: $p = 0.31$.

Supplementary Table 2.

Same as Supplementary Table 1., but statistical results used in Extended Data Figures.

**1) Figure panel**	**2) Data type, group**	**3) Test type**	**4) Statistical info., Degree of freedom, P values**
Extended Data Fig. 4f	Number of microablated neurons per mouse across experimental groups.	One-way ANOVA test across groups.	$F(2, 23) = 0.47$, $p = 0.63$.
Extended Data Fig. 4g	Number of high category neurons per mouse.	One-way ANOVA test across groups.	$F(3, 30) = 0.56$, $p = 0.65$.
Extended Data Fig. 4h	Number of low category neurons per mouse.	One-way ANOVA test across groups.	$F(3, 30) = 0.12$, $p = 0.95$.
Extended Data Fig. 4i	Response amplitude of microablated neurons averaged during baseline days.	Two-sided *t*-test with FDR correction.	Sound responsive ablation vs. non-responsive ablation: $p = 2.68\times10^{-9}$;<br>Sound responsive ablation vs. inhibitory neuron ablation: $p = 2.99\times10^{-5}$;<br>Non-responsive ablation vs. inhibitory neuron ablation: $p = 3.90\times10^{-9}$.
Extended Data Fig. 4k	Response amplitude of low category neurons during baseline days.	One-way ANOVA test across groups.	$F(3, 30) = 1.98$, $p = 0.14$.
Extended Data Fig. 4l	Number of spared neurons per FOV considered for analysis.	One-way ANOVA test across groups.	$F(3, 31) = 0.25$, $p = 0.86$.
Extended Data Fig. 5a	Normalized correlations averaged across diagonal elements and non-diagonal elements.	Two-sided t-test between baseline days vs. days after ablation with FDR correction.	Average of diagonal elements: $p = 0.93$, $p = 0.93$, $p = 0.53$, $p = 0.084$, for day 7, 9, 11, 15, respectively;<br>Average of non-diagonal elements: $p = 0.88$, $p = 0.28$, $p = 0.0072$, $p = 0.0072$ for day 7, 9, 11, 15, respectively.
Extended Data Fig. 5e	Cumulative distribution of baseline normalized correlation coefficient.	Dunn's test.	$Q_{critical} = 2.39$<br>all Q values between groups for both diagonal and non-diagonal components in the similarity matrices, $Q < Q_{critical}$.
		Mann-Whitney *U* test between groups with FDR correction.	The diagonal and the non-diagonal components, all *p* values, $p > 0.5$.
Extended Data Fig. 5f	Cumulative distribution of baseline normalized correlation coefficient on day 7 in the representational map.	Dunn's test.	Diagonal components, $Q_{critical} = 2.39$:<br>Sound responsive ablation vs. Control: $Q = 2.93$;<br>Sound responsive ablation vs. Non-responsive ablation: $Q < Q_{critical}$;<br>Non-responsive ablation vs. Control: $Q < Q_{critical}$;<br><br>Non-diagonal components:<br>Sound responsive ablation vs. Control: $Q = 3.44$;<br>Sound responsive ablation vs. Non-responsive ablation: $Q = 2.57$;<br>Non-responsive ablation vs. Control: $Q < Q_{critical}$.
		Mann-Whitney *U* test with FDR	Diagonal components:

		correction between experimental cohorts.	Sound responsive ablation vs. Control: p = 0.016;<br>Sound responsive ablation vs. Non-responsive ablation: p = 0.077;<br>Non-responsive ablation vs. Control: p = 0.20;<br><br>Non-diagonal components:<br>Sound responsive ablation vs. Control: p = 0.0018;<br>Sound responsive ablation vs. Non-responsive ablation: p = 0.015;<br>Non-responsive ablation vs. Control: p = 0.33.
Extended Data Fig. 5k	Simulated non-diagonal correlation of similarity matrix.	Two-sided t-test between baseline correlation vs. simulated correlation after downscaling.	Sound responsive cohort: p = 0.0083;<br>Non-sound responsive cohort: p = 0.19;<br>Control: p = 0.27.
		Two-sided t-test between simulated correlation after downscaling vs. correlation on day 7.	Sound responsive: p = 0.42;<br>Non-sound responsive: p = 0.89;<br>Control cohort: p = 0.58.
Extended Data Fig. 6a	Baseline-normalized Pearson correlations of population vectors averaged across diagonal elements (left) and non-diagonal elements (right), split into pure tones (blue) and complex sounds (red), for sound responsive ablation cohort.	Two-way ANOVA between PT and CS stimuli and across days.	Diagonal elements: F(1, 132) = 0.31, p = 0.58 between PT and CS stimuli; F(6, 132) = 1.52, p = 0.18 across days.<br>Non-diagonal elements: F(1, 132) = 0.13, p = 0.72 between PT and CS stimuli; F(6, 132) = 1.32, p = 0.25 across days.
		Two-sided t-test between PT and CS stimuli with FDR correction across post-ablation days.	Diagonal elements: $p > 0.65$;<br>Non-diagonal elements: $p > 0.57$.
Extended Data Fig. 6b	Normalized response reliability across trials for PT and CS stimuli for sound responsive ablation cohort.	Two-way ANOVA between PT and CS tunings.	F(1, 132) = 0.014, p = 0.91 between PT and CS tunings.
		Two-sided t-test of reliability between PT response vs. CS response with FDR correction across post ablation days.	day 7, 9, 11, 15: $p > 0.50$;
Extended Data Fig. 6c	Fraction of responsiveness for PT and CS stimuli for sound responsive ablation cohort.	Two-way ANOVA between PT and CS stimuli and across days.	F(1, 132) = 1.41, p = 0.24 between PT and CS stimuli;<br>F(6, 132) = 1.86, p = 0.092 across days.
		Two-sided t-test of response fraction between PT response vs. CS response with FDR correction across post ablation days.	day 7, 9, 11, 15: $p > 0.92$.
	Maximum response amplitude for PT and	Two-way ANOVA between PT and CS	F(1, 132) = 3.79, p = 0.054 between PT and CS tunings;

Extended Data Fig. 6d	CS stimuli in sound responsive neurons.	tunings and across days.	F(6, 132) = 8.78, $p = 4.69\times10^{-8}$ across days.
		Two-sided *t*-test of maximum amplitude between PT response vs. CS response with FDR correction across post ablation days.	day 7, 9, 11, 15: $p > 0.42$.
Extended Data Fig. 6e	Normalized tuning curves for pure tones (left) and complex sounds (right) overlaid across days.	Two-sided *t*-test with FDR correction between PT tuning vs. CS tuning at 15th stimulus index.	day 7, 9, 11, 15: $p = 0.39$, $p = 0.39$, $p = 0.41$, $p = 0.27$.
Extended Data Fig. 6f	Stability of single-trial responses between PT and CS stimuli analyzed analogous to a previous study[43].	Two-way ANOVA between PT and CS tunings and across days.	F(1, 132) = 0.58, $p = 0.45$ between PT and CS tunings;<br>F(6, 132) = 14.15, $p = 2.13\times10^{-12}$ across days.
		Two-sided *t*-test of single-trial stability between PT and CS stimuli	day 1, 3, 5: $p > 0.32$;<br>day 7, 9, 11, 15: $p > 0.56$.
Extended Data Fig. 7a	Best response amplitude of neurons responsive on both day 5 and day 5±i normalized by the average best response during early baseline days (1 and 3).	One-way ANOVA test across days.	Sound responsive ablation: F(5, 54) = 1.29, $p = 0.28$;<br>Non-sound responsive ablation: F(5, 54) = 1.35, $p = 0.26$;<br>Control: F(5, 48) = 0.58, $p = 0.72$.
Extended Data Fig. 7c	Spatial extent of the microablation-induced effect on normalized best response amplitudes in the spared neurons. Spared neurons were split into neurons responsive on day 5 (Left).	In the control group, normalized best responses of neurons responsive on day 5, Two-sided *t*-test between short and long distance, across days.	day 1 to day 15: $p = 0.13$, $p = 0.82$, $p = 0.14$, $p = 0.049$, $p = 0.061$, $p = 0.0014$, $p = 0.0012$.
	Spatial extent of the microablation-induced effect on normalized best responses of spared neurons newly responsive neurons (Right) across distance during and after baseline days.	Two-sided *t*-test between short and long distance.	day 1, $p = 0.066$; day 3, $p = 0.014$; day 7, $p = 0.96$; day 9, $p = 0.13$; day 11, $p = 0.080$; day 15, $p = 0.19$.
Extended Data Fig. 7d	Normalized best responses of neurons responsive on day 5 in sound responsive ablation cohort (left).	Two-way ANOVA, across distance bins and across days.	Neurons responsive on day 5:<br>F(6, 244429) = 0.45, $p = 0.85$ across distance bins;<br>F(6, 244429) = 151.58, $p < 1.0\times10^{-10}$ across days.

	Normalized best responses of neurons not responsive on day 5 but newly responsive on another day (right).	Two-way ANOVA across distance bins and across days.	Neurons unresponsive on day 5 but newly responsive on another day:<br>F(6, 121590) = 10.9, $p = 3.56\times10^{-12}$ across distance bins;<br>F(5, 121590) = 543.19, $p < 1.0\times10^{-12}$ across days.
		Two-sided *t*-test between short (< 100 μm) and long distance (250-350 μm).	day 1: $p = 0.49$; day 3: $p = 0.42$; day 7: $p = 0.0001$; day 9: $p = 1.0\times10^{-5}$; day 11: $p = 0.0002$; day 15: $p = 0.12$.
Extended Data Fig. 7e	Normalized best responses of neurons responsive on day 5 for non-responsive ablation cohort (left).	Two-way ANOVA across distance bins and across days.	F(6, 284429) = 9.47, $p = 1.99\times10^{-10}$ across distance bins;<br>F(6, 284429) = 230.36, $p < 1.0\times10^{-10}$ across days;
		Two-sided *t*-test between short and long distance.	day 1, $p = 0.012$; day 3, $p = 0.50$; day 5, $p = 0.031$; day 7, $p < 1.0\times10^{-4}$; day 9, $p = 0.0005$; day 11, $p = 0.0003$; day 15, $p = 0.0004$.
	Normalized best responses of newly responsive neurons for non-responsive ablation cohort (right).	Two-way ANOVA across distance bins and across days.	F(6, 146974) = 1.85, $p = 0.085$ across distance bins;<br>F(5, 146974) = 274.42, $p = 1.0\times10^{-10}$ across days.
		Two-sided *t*-test between short and long distance.	day 1, $p = 0.26$; day 3, $p = 0.036$; day 7, $p < 1.0\times10^{-5}$; day 9, $p = 0.63$; day 11, $p = 0.012$; day 15, $p = 0.085$.
Extended Data Fig. 7f	Normalized best responses of neurons responsive on day 5 for inhibitory neuron ablation (left).	Two-way ANOVA across distance bins and across days.	F(6, 212481) = 1.08, $p = 0.37$ across distance bins;<br>F(6, 212481) = 348.61, $p < 1.0\times10^{-10}$ across days;
	Normalized best responses of newly responsive neurons for inhibitory neuron ablation (right).	Two-way ANOVA across distance bins and across days.	F(6, 96363) = 0.84, $p = 0.54$ across distance bins;<br>F(5, 96363) = 78.00, $p = 6.23\times10^{-82}$ across days.
Extended Data Fig. 7g	Normalized tuning curves of day 5 responsive neurons (top) for sound responsive cohort (left), non-sound responsive cohort (middle) and control cohort (right), respectively.	Two-sided *t*-test of normalized amplitude at 15th stimulus between baseline days and post ablation days with FDR correction.	Sound responsive cohort,<br>neurons responsive on day 5:<br>day 7: $p = 0.011$; day 9: $p = 0.25$; day 11: $p = 0.011$; day 15: $p = 0.0010$;<br><br>Non-sound responsive cohort,<br>neurons responsive on day 5:<br>day 7: $p = 0.85$; day 9: $p = 0.73$; day 11: $p = 0.85$; day 15: $p = 0.48$;<br><br>Control cohort,<br>neurons responsive on day 5<br>day 7: $p = 0.19$; day 9: $p = 0.080$; day 11: $p = 0.34$; day 15: $p = 0.72$;
	Normalized tuning curves of neurons unresponsive on day 5 but responsive on the other day (bottom), for sound responsive cohort (left), non-sound	Two-sided *t*-test of normalized amplitude at 15th stimulus between baseline days and post ablation days with FDR correction.	Sound responsive cohort,<br>neurons not responsive on day 5 but responsive on another day:<br>day 7: $p = 0.14$; day 9: $p = 0.57$; day 11: $p = 0.57$; day 15: $p = 0.034$;<br><br>Non-sound responsive cohort,

	responsive cohort (middle) and control cohort (right), respectively.		neurons not responsive on day 5 but responsive on another day:<br>day 7: $p = 0.82$; day 9: $p = 0.82$; day 11: $p = 0.82$; day 15: $p = 0.52$;<br><br>Control cohort,<br>neurons not responsive on day 5 but responsive on another day:<br>day 7: $p = 0.51$; day 9: $p = 0.65$; day 11: $p = 0.64$; day 15: $p = 0.64$;
Extended Data Fig. 7i	Change in the fraction of neuron pairs with high signal correlation from baseline at the largest response amplitude bin.	Two-sided *t*-test between baseline days and days after ablation with FDR correction.	Sound responsive cohort: $p = 0.243$, $p = 0.067$, $p = 0.015$, $p = 0.018$ for day 7, 9, 11, 15;<br>Non-sound responsive cohort: $p = 0.12$, $p = 0.19$, $p = 0.12$, $p = 0.0055$ for day 7, 9, 11, 15;<br>Control cohort: $p > 0.40$ for all post-sham ablation days.
		Permutation test for group comparison.	$p > 0.05$ from day 7 - 11 for all the three cohorts. $p < 0.05$ on day 15 for sound responsive cohort.
Extended Data Fig. 7j	Cumulative distribution of all pairwise signal correlations between high category neurons and spared neurons	One-way ANOVA test of signal correlation (> 0.6) over days	$F(6,63) = 0.48$, $p = 0.82$
Extended Data Fig. 8e	Baseline-normalized ratio of the total sound-evoked activity in excitatory and inhibitory neurons (sound responsive cohort: $n = 5$; non-sound responsive cohort: $n = 5$, control: $n = 7$).	Two-sided *t*-test with FDR adjusted p-values between sound responsive cohort vs. control, between non-sound responsive cohort vs. control, and between sound responsive cohort vs. non-sound responsive cohort.	day 7: $p > 0.5$ for all combinations;<br>day 9: $p = 0.039$, $p = 0.039$, $p = 0.16$;<br>day 11: $p = 0.045$, $p = 0.032$, $p = 0.27$;<br>day 15: $p > 0.5$ for all combinations, respectively;<br><br>(The normalized ratio of best amplitudes on day 15, 1.09 ± 0.088 for sound responsive ablation, 1.10 ± 0.11 for non-sound responsive ablation.)
Extended Data Fig. 8g	Traces of detected calcium transients of an inhibitory neuron over days.<br>Decay time constant of calcium transients of all identified interneurons over days across mice.	One-way ANOVA test over the imaging days.	$F(6, 28) = 0.48$; $p = 0.82$.
Extended Data Fig. 8h	Change of tuning width by using normalized response amplitudes at 15th largest stimulus index in the tuning curve over days for excitatory (left) neurons.	Two-sided *t*-test of Δnormalized amplitude for excitatory neurons between baseline days vs. days after ablation with FDR correction.	Sound responsive cohort: $p = 0.011$, $p = 0.25$, $p = 0.35$, $p = 0.011$, for day 7, 9, 11, 15, respectively;<br>Non-sound responsive cohort: $p > 0.47$ for all post-ablation days;<br>Control: $p > 0.67$ for all post-ablation days.
	Change of tuning width by using normalized response amplitudes at 15th largest stimulus index in the tuning	Two-sided *t*-test of Δnormalized amplitude for inhibitory neurons.	Sound responsive cohort: $p = 0.21$, $p = 0.88$, $p = 0.33$, $p = 0.072$ for day 7, 9, 11, 15, respectively;<br>Non-sound responsive cohort: $p > 0.20$ for all post-ablation days;

	curve over days for inhibitory (right) neurons.		Control: $p > 0.24$ for all post-ablation days.
Extended Data Fig. 8i	Change in fraction of neuron pairs with high signal correlation and with large response amplitude from baseline among excitatory-excitatory neurons (left).	Two-sided *t*-test of Δfraction of excitatory-excitatory neuron pairs with FDR correction.	Sound responsive cohort: $p = 0.15$, $p = 0.051$, $p = 0.11$, $p = 0.0034$ for day 7, 9, 11, 15; Non-sound responsive cohort: $p > 0.29$ for all post-ablation days; Control: $p > 0.43$ for all post-ablation days.
		Permutation test for group comparison.	Sound responsive cohort: $p > 0.05$ for day 7, 9, 11, $p < 0.025$ on day 15; Both non-sound responsive and control cohorts: $p > 0.05$ for all post-ablation days.
	Change in fraction of neuron pairs with high signal correlation and with large response amplitude from baseline among inhibitory-inhibitory neurons (middle).	Two-sided *t*-test of Δfraction of inhibitory- inhibitory neuron pairs with FDR correction.	Sound responsive cohort: $p > 0.15$ for all post-ablation days; Non-sound responsive cohort: $p > 0.28$ for all post-ablation days; Control: $p > 0.16$ for all post-ablation days.
	Change in fraction of neuron pairs with high signal correlation and with large response amplitude from baseline among excitatory-inhibitory neurons (right).	Two-sided *t*-test of Δfraction of excitatory-inhibitory neuron pairs with FDR correction.	sound responsive cohort: $p > 0.25$ for all post-ablation days; Non-sound responsive cohort: $p > 0.30$ for all post-ablation days; Control: $p > 0.26$ for all post-ablation days.
Extended Data Fig. 8j	Baseline-normalized best response amplitude of neurons responsive on day 5 in excitatory (left) neurons for the three experimental groups.	One-way ANOVA across days, for excitatory neurons.	sound responsive ablation, $F(6, 28) = 4.21$, $p = 0.0038$; Non-responsive ablation, $F(6, 28) = 1.01$, $p = 0.43$; Control, $F(6, 42) = 1.36$, $p = 0.25$;
		Two-sided paired *t*-test of the normalized best amplitude of excitatory neurons between day 3 vs. day 7.	Sound responsive ablation, $p = 0.023$; Non-responsive ablation, $p = 0.34$; Control, $p = 0.38$.
	Baseline-normalized best response amplitude of neurons responsive on day 5 in inhibitory (right) neurons for the three experimental groups.	One-way ANOVA across days, for inhibitory neurons.	Sound responsive ablation: $F(6, 21) = 7.44$, $p = 2.3\times10^{-4}$; Non-responsive ablation: $F(6, 28) = 0.51$, $p = 0.80$; Control: $F(6, 42) = 1.02$, $p = 0.42$;
		Two-sided paired *t*-test of the normalized best amplitude of inhibitory neurons between day 3 vs. day 7.	Sound responsive ablation: $p = 0.049$; Non-responsive ablation: $p = 0.38$; Control: $p = 0.59$.

Extended Data Fig. 8k	Baseline-normalized best response amplitude of the excitatory neurons (left) unresponsive on day 5 but being responsive on the other day.	One-way ANOVA across days, in excitatory neurons.	Sound responsive ablation: $F(5, 24) = 7.97$, $p = 1.5\times10^{-4}$.<br>Non-responsive ablation: $F(5, 24) = 1.12$, $p = 0.37$;<br>Control: $F(5, 36) = 0.28$, $p = 0.92$;
		Group comparison by two-sided *t*-test with FDR correction.	Sound responsive ablation vs. non-responsive ablation; Sound responsive ablation vs. control; Non-responsive ablation vs. control; day 7 and day 9: $p > 0.2$ for all combinations; day 11: $p = 0.011$; $p = 0.024$; $p = 0.93$; day 15: $p = 0.14$; $p = 0.011$; $p = 0.19$, respectively.
	Baseline-normalized best response amplitude of the inhibitory neurons (right) unresponsive on day 5 but being responsive on the other day.	One-way ANOVA across days.	Inhibitory neurons in<br>Sound responsive ablation: $F(5, 24) = 1.01$, $p = 0.43$;<br>Non-responsive ablation: $F(5, 23) = 1.18$, $p = 0.35$;<br>Control: $F(5, 25) = 1.44$, $p = 0.24$.
Extended Data Fig. 9a	Cumulative distributions of normalized correlation on day 5 along diagonal elements and non-diagonal elements for sound responsive, inhibitory neuron ablation and control cohorts.	Dunn's test for all group combinations in both diagonal and non-diagonal elements.	Q < Qcritical.
		Mann-Whitney *U* test between groups with adjusted p-values by FDR correction.	p values > 0.5 for all combinations.
Extended Data Fig. 9b	Cumulative distributions of normalized correlation on day 7 along diagonal elements and non-diagonal elements for sound responsive, inhibitory neuron ablation and control cohorts.	Dunn's test for group comparison.	Diagonal elements:<br>Sound responsive ablation vs. control, Q = 3.00 (> Qcritical);<br>Inhibitory neuron ablation vs. control, Q < Qcritical.<br><br>Non-diagonal elements:<br>sound responsive ablation vs. control, Q = 3.48 (> Qcritical);<br>Q < Qcritical for the other group comparison.
		Mann-Whitney *U* test between groups, with adjusted p-values by FDR correction, along diagonal and non-diagonal elements.	Diagonal elements:<br>Sound responsive ablation vs. control: $p = 0.016$; Inhibitory vs. control: $p = 0.020$;<br>Sound responsive ablation vs. inhibitory: $p = 0.51$.<br><br>Non-diagonal elements:<br>Sound responsive ablation vs. control: $p = 0.0018$;<br>Inhibitory neuron ablation vs. control: $p = 0.06$; Sound responsive ablation vs. inhibitory neuron ablation: $p = 0.29$.
Extended Data Fig. 9c	Cumulative distributions of normalized correlation during late after	Dunn's test along diagonal elements.	Inhibitory neuron ablation vs control: Q = 2.44 (> Qcritical);<br>Sound responsive ablation vs. inhibitory neuron ablation: Q = 2.43 (> Qcritical).

	microablation, day 9-11.	Mann-Whitney *U* test, adjusted *p*-values.	Sound responsive ablation vs. control, p = 0.87; Inhibitory neuron ablation vs. control, p = 0.056; Sound responsive ablation vs. inhibitory neuron ablation, p = 0.056.
Extended Data Fig. 9d	Normalized tuning curve of responsive neurons overlaid across days in inhibitory neuron ablation.	Two-sided *t*-test of average of normalized amplitudes across the stimulus with 2nd largest amplitude to the last stimulus between baseline days vs. day 7.	p = 0.20.
Extended Data Fig. 9e	Normalized non-diagonal correlations in the similarity matrix constructed from the population vectors shuffled across FOVs in inhibitory neuron ablation.	Two-sided *t*-test of normalized correlation during all post-ablation days between inhibitory neuron ablation and control.	p = 0.33.
Extended Data Fig. 9f	The colormap of the change in fraction of responsive neuron pairs with high signal correlation from baseline according to the best response amplitude in inhibitory neuron ablation cohort.	Two-way ANOVA across days and across amplitude bins.	$F(6, 154) = 2.05$, p = 0.062 across days; $F(3, 154) = 5.10$, p = 0.0022 across amplitude bins.

Supplementary Table 3.

n number in the analysis for distance dependence of response amplitude in Extended Data Fig. 7**c**-**f**.

**Sound responsive ablation**								
▪ Neurons responsive on day 5								
	Distance, μm	15-50	50-100	100-150	150-200	200-250	250-300	300-350
	day 5	811	4118	7540	8440	6943	4923	2293
▪ Neurons unresponsive on day 5 but responsive on other days								
	Distance, μm	15-50	50-100	100-150	150-200	200-250	250-300	300-350
days	day 1	560	2780	4551	5389	4727	3359	1489
	day 3	500	2562	4379	5108	4606	3349	1641
	day 5	0	0	0	0	0	0	0
	day 7	404	2103	3789	4334	3704	2549	1166
	day 9	499	2466	4579	5125	4362	2911	1341
	day 11	454	2337	4101	4578	4001	2734	1155
	day 15	446	2282	3981	4147	3511	2408	1135
**Non-sound responsive ablation**								
▪ Neurons responsive on day 5								
	Distance, μm	15-50	50-100	100-150	150-200	200-250	250-300	300-350
	day 5	1112	5253	9003	10052	8242	5903	2820
▪ Neurons unresponsive on day 5 but responsive on other days								
	Distance, μm	15-50	50-100	100-150	150-200	200-250	250-300	300-350
days	day 1	752	3726	6609	7240	6256	4506	2065
	day 3	675	3491	5858	6623	5608	4048	2085
	day 5	0	0	0	0	0	0	0
	day 7	541	2500	4493	4859	4357	3111	1631
	day 9	592	2749	4862	5322	4667	3212	1552
	day 11	602	2942	5362	5744	4716	3248	1458
	day 15	638	3076	5586	5999	4827	3208	1424
**Control**								
▪ Neurons responsive on day 5								
	Distance, μm	15-50	50-100	100-150	150-200	200-250	250-300	300-350
	day 5	802	4040	7020	7789	6503	4476	2143
▪ Neurons unresponsive on day 5 but responsive on other days								
	Distance, μm	15-50	50-100	100-150	150-200	200-250	250-300	300-350
days	day 1	533	2592	4603	4989	4173	2826	1351
	day 3	465	2366	4175	4917	4006	2898	1394
	day 5	0	0	0	0	0	0	0
	day 7	417	2096	3708	4217	3468	2508	1111
	day 9	403	1971	3381	3892	3352	2395	1124
	day 11	390	2057	3719	4248	3592	2568	1254
	day 15	414	2186	3746	4526	3821	2686	1189
**Inhibitory neuron ablation**								
▪ Neurons responsive on day 5								
	Distance, μm	15-50	50-100	100-150	150-200	200-250	250-300	300-350
	day 5	646	3125	5762	6728	5945	4208	2019
▪ Neurons unresponsive on day 5 but responsive on other days								

	Distance, µm	15-50	50-100	100-150	150-200	200-250	250-300	300-350
days	day 1	414	1881	3566	4175	3689	2653	1279
	day 3	391	1926	3532	3971	3610	2603	1308
	day 5	0	0	0	0	0	0	0
	day 7	261	1267	2323	2777	2466	1730	883
	day 9	268	1359	2471	2974	2630	1909	982
	day 11	301	1479	2846	3287	2928	2202	1191
	day 15	303	1470	2806	3153	2795	2109	1197
