# Supplementary material for: Homeostasis of a representational map in the neocortex
Source: Nat Neurosci. 2025 Jun 5;28(7):1533–45. doi: 10.1038/s41593-025-01982-7 (PMC12229895; doi:10.1038/s41593-025-01982-7)
Supplement: Supplementary file 2 — Reporting Summary [file 41593_2025_1982_MOESM2_ESM.pdf]

Corresponding author(s): Simon Rumpel
Last updated by author(s): 2025-3-18

Reporting Summary

Nature Portfolio wishes to improve the reproducibility of the work that we publish. This form provides structure for consistency and transparency in reporting. For further information on Nature Portfolio policies, see our Editorial Policies and the Editorial Policy Checklist.

Statistics

For all statistical analyses, confirm that the following items are present in the figure legend, table legend, main text, or Methods section.

n/a	Confirmed	
☐	☒	The exact sample size (*n*) for each experimental group/condition, given as a discrete number and unit of measurement
☐	☒	A statement on whether measurements were taken from distinct samples or whether the same sample was measured repeatedly
☐	☒	The statistical test(s) used AND whether they are one- or two-sided<br>*Only common tests should be described solely by name; describe more complex techniques in the Methods section.*
☒	☐	A description of all covariates tested
☐	☒	A description of any assumptions or corrections, such as tests of normality and adjustment for multiple comparisons
☐	☒	A full description of the statistical parameters including central tendency (e.g. means) or other basic estimates (e.g. regression coefficient) AND variation (e.g. standard deviation) or associated estimates of uncertainty (e.g. confidence intervals)
☐	☒	For null hypothesis testing, the test statistic (e.g. *F*, *t*, *r*) with confidence intervals, effect sizes, degrees of freedom and *P* value noted<br>*Give P values as exact values whenever suitable.*
☒	☐	For Bayesian analysis, information on the choice of priors and Markov chain Monte Carlo settings
☐	☒	For hierarchical and complex designs, identification of the appropriate level for tests and full reporting of outcomes
☒	☐	Estimates of effect sizes (e.g. Cohen's *d*, Pearson's *r*), indicating how they were calculated

Our web collection on statistics for biologists contains articles on many of the points above.

Software and code

Policy information about availability of computer code

Data collection	Two-photon imaging: Prairie View Imaging ver4.2, Intrinsic signal imaging: a custom-written code in MATLAB 2019b; Confocal imaging: Leica Application Suite Advanced Fluorescence (LAS AF).
Data analysis	Custom written codes in MATLAB versions R2016b and R2022a (Mathworks), Image J/Fiji (ImageJ 1.53t, Java 1.8.0_172 (64bit), NIH, http://imagej.net/Welcome). Optimal two-dimensional affine transformation for ROI registration was applied by using Nelder-Mead-Simplex algorithm with MATLAB function, fminsearch in MATLAB R2016b. Primary analysis pipelines also are publicly available at G-Node (G-Node: https://doi.org/10.12751/g-node.2gxcrx, 'tutorials' directory).

For manuscripts utilizing custom algorithms or software that are central to the research but not yet described in published literature, software must be made available to editors and reviewers. We strongly encourage code deposition in a community repository (e.g. GitHub). See the Nature Portfolio guidelines for submitting code & software for further information.

Data

Policy information about availability of data

All manuscripts must include a data availability statement. This statement should provide the following information, where applicable:

- Accession codes, unique identifiers, or web links for publicly available datasets
- A description of any restrictions on data availability
- For clinical datasets or third party data, please ensure that the statement adheres to our policy

The data of calcium activities, region of interest (roi) information of neurons, auditory stimulations are publicly available at G-Node (G-Node: https://doi.org/10.12751/g-node.2gxcrx, 'data' directory).

Research involving human participants, their data, or biological material

Policy information about studies with human participants or human data. See also policy information about sex, gender (identity/presentation), and sexual orientation and race, ethnicity and racism.

Reporting on sex and gender	N/A
Reporting on race, ethnicity, or other socially relevant groupings	N/A
Population characteristics	N/A
Recruitment	N/A
Ethics oversight	N/A

Note that full information on the approval of the study protocol must also be provided in the manuscript.

Field-specific reporting

Please select the one below that is the best fit for your research. If you are not sure, read the appropriate sections before making your selection.

☒ Life sciences ☐ Behavioural & social sciences ☐ Ecological, evolutionary & environmental sciences

For a reference copy of the document with all sections, see nature.com/documents/nr-reporting-summary-flat.pdf

Life sciences study design

All studies must disclose on these points even when the disclosure is negative.

Sample size	For each experiment, the sample size was determined based on literature in the field and prior experience with the applied analyses (e.g. Bathellier et al., Neuron 2011; Aschauer et al., Cell. Rep 2022). The precise number of animals used are given in the results, figures and figure legends.
Data exclusions	As noted in the manuscript, we excluded neurons which did not exhibit reliably present nucleus signal defined by the following 4 criteria: Neighbor Distance, Normalized Soma Signal Intensity, Soma Signal to Noise ratio and Objective Function Value (see Aschauer et al., Cell. Rep 2022). We excluded individual FOVs where number of reliably detected neurons based on the 4 criteria was less than 100 (see Methods for details) .
Replication	Experiments were repeated in individual mice so that our data are based on multiple independent repeats with similar results. As given in the methods and results, the precise numbers of independent repeats for the four experimental cohorts of mice are as follows: n = 10 for sound responsive ablation cohort; n = 10 for non-sound responsive ablation cohort; n = 9 for control cohort; n = 6 for inhibitory neuron ablation cohort.
Randomization	Research animals were temporally psudo-randomly allocated in the four experimental cohorts in this study.
Blinding	The investigators were not blinded to group allocation.

Reporting for specific materials, systems and methods

We require information from authors about some types of materials, experimental systems and methods used in many studies. Here, indicate whether each material, system or method listed is relevant to your study. If you are not sure if a list item applies to your research, read the appropriate section before selecting a response.

Materials & experimental systems

n/a	Involved in the study
☒	☐ Antibodies
☐	☒ Eukaryotic cell lines
☒	☐ Palaeontology and archaeology
☐	☒ Animals and other organisms
☒	☐ Clinical data
☒	☐ Dual use research of concern
☒	☐ Plants

Methods

n/a	Involved in the study
☒	☐ ChIP-seq
☒	☐ Flow cytometry
☒	☐ MRI-based neuroimaging

Eukaryotic cell lines

Policy information about cell lines and Sex and Gender in Research

Cell line source(s)	HEK 293 cells to produce rAAV vectors by using a helper virus free, two-plasmid based production method (Grimm et al., 2003).
Authentication	N/A
Mycoplasma contamination	N/A
Commonly misidentified lines (See ICLAC register)	N/A

Animals and other research organisms

Policy information about studies involving animals; ARRIVE guidelines recommended for reporting animal research, and Sex and Gender in Research

Laboratory animals	4-6 weeks of age in C57BL/6JRj mice, 16 weeks of age in GAD67-GFP mice. Mice were housed and acclimated under standard conditions (12 h light/ dark cycle, temperature 22 ± 2 ∘C, relative humidity 55 ± 10 %).
Wild animals	No wild animals were used in the study.
Reporting on sex	M
Field-collected samples	No field collected samples were used in the study.
Ethics oversight	All animal experiments were performed in accordance with the animal law guidelines for animal research and had been approved by the Landesuntersuchungsamt Rheinland Pfalz (Approval 23 177-07/G 17-1-051).

Note that full information on the approval of the study protocol must also be provided in the manuscript.

Plants

Seed stocks	N/A
Novel plant genotypes	N/A
Authentication	N/A
